# Ultrasound May Suppress Tumor Growth, Inhibit Inflammation, and Establish Tolerogenesis by Remodeling Innatome via Pathways of ROS, Immune Checkpoints, Cytokines, and Trained Immunity/Tolerance

**DOI:** 10.1155/2021/6664453

**Published:** 2021-02-09

**Authors:** Qian Yang, Ruijing Zhang, Peng Tang, Yu Sun, Candice Johnson, Jason Saredy, Susu Wu, Jiwei Wang, Yifan Lu, Fatma Saaoud, Ying Shao, Charles Drummer, Keman Xu, Daohai Yu, Rongshan Li, Shuping Ge, Xiaohua Jiang, Hong Wang, Xiaofeng Yang

**Affiliations:** ^1^Centers for Cardiovascular Research and Inflammation, Translational, & Clinical Lung Research, Lewis Katz School of Medicine at Temple University, Philadelphia, PA 19140, USA; ^2^Department of Ultrasonic Diagnosis and Treatment Center, XiAn International Medical Center Hospital, XiAn, China; ^3^Heart Center, St. Christopher's Hospital for Children, Drexel University College of Medicine, Philadelphia, PA, USA; ^4^Department of Nephrology, Second Hospital of Shanxi Medical University, Shanxi Provincial People's Hospital, Taiyuan, Shanxi, China; ^5^Department of Orthopedics, Beijing Charity Hospital of China Rehabilitation Research Center, Beijing, China; ^6^Metabolic Disease Research & Thrombosis Research, Departments of Pharmacology, Microbiology and Immunology, Lewis Katz School of Medicine at Temple University, Philadelphia, PA 19140, USA; ^7^Department of Clinical Sciences, Lewis Katz School of Medicine at Temple University, Philadelphia, PA 19140, USA

## Abstract

**Background:**

The immune mechanisms underlying low-intensity ultrasound- (LIUS-) mediated suppression of inflammation and tumorigenesis remain poorly determined.

**Methods:**

We used microarray datasets from the NCBI GEO DataSet repository and conducted comprehensive data-mining analyses, where we examined the gene expression of 1376 innate immune regulators (innatome genes (IGs) in cells treated with LIUS.

**Results:**

We made the following findings: (1) LIUS upregulates proinflammatory IGs and downregulates metastasis genes in cancer cells, and LIUS upregulates adaptive immunity pathways but inhibits danger-sensing and inflammation pathways and promote tolerogenic differentiation in bone marrow (BM) cells. (2) LIUS upregulates IGs encoded for proteins localized in the cytoplasm, extracellular space, and others, but downregulates IG proteins localized in nuclear and plasma membranes, and LIUS downregulates phosphatases. (3) LIUS-modulated IGs act partially via several important pathways of reactive oxygen species (ROS), reverse signaling of immune checkpoint receptors B7-H4 and BTNL2, inflammatory cytokines, and static or oscillatory shear stress and heat generation, among which ROS is a dominant mechanism. (4) LIUS upregulates trained immunity enzymes in lymphoma cells and downregulates trained immunity enzymes and presumably establishes trained tolerance in BM cells. (5) LIUS modulates chromatin long-range interactions to differentially regulate IGs expression in cancer cells and noncancer cells.

**Conclusions:**

Our analysis suggests novel molecular mechanisms that are utilized by LIUS to induce tumor suppression and inflammation inhibition. Our findings may lead to development of new treatment protocols for cancers and chronic inflammation.

## 1. Introduction

Ultrasound, alone or combined with contrast agent microbubbles, has numerous applications, which range from being well-established diagnostic tools [1, 2] to methods of drug delivery [3]. The application of microbubbles and ultrasound to deliver nanoparticle carriers for drug and gene delivery is a research area that has significantly expanded in recent years. Recent reports showed that utilization of ultrasound contrast microbubbles causes the so-called “sonoporation” effect [4, 5], which has been recognized to cause transient disruption of cellular membranes [6], allowing more accessible transport of extracellular compounds into the cytoplasm of viable cells [7]. Ultrasound therapy is now widely used in clinical practice in the treatment of various human malignancies and inflammatory diseases and in promoting tissue repair in leukemia, lymphoma, melanoma, breast cancer, pancreatic neuroendocrine tumors [8], hepatic cancer, nasopharyngeal cancers, colon cancer, gastric cancer, glioma, ovarian cancer, [9], sarcoma [10–12], stroke [13], prostatic hyperplasia, renal masses [14], abdominal subcutaneous adipose tissue [15], bone repair [16], osteoarthritis [17], and carpal tunnel syndrome [18]. So far, several therapeutic ultrasound methods have been developed including high-intensity focused ultrasound [10] and low-intensity pulsed ultrasound [19]. Recently, several clinical trials and experimental reports have confirmed the capacity of ultrasound to elicit anti-inflammatory and tissue repair/regeneration responses [20, 21], suggesting the potential of using ultrasound as a novel therapeutic method [6, 22–25].

It is now recognized that inflammation induced by pathogen-associated molecular patterns (PAMPs) [26] and danger/conditional danger-associated molecular patterns (conditional DAMPs) [27] is an essential mechanism of innate immune response [28]. Classical danger/damage-associated molecular patterns (DAMPs) bind to various innate immune pattern receptors such as Toll-like receptors and NOD-like receptors [29–31], whereas pathologically elevated endogenous metabolite-derived DAMPs that bind to their own receptors are termed as conditional DAMPs as we proposed in 2016 [27, 32]. We recently proposed that vascular endothelial cells are innate immune cells [30, 33]. Recent reports from our and others' laboratories report several novel concepts: (1) cardiovascular tissues have an inflammation privilege that requires chronic upregulation of innate immune sensors for cardiovascular disease risk factor-related DAMPs/conditional DAMPs [34]; (2) aortic endothelial cells [35], endothelial progenitor cells [36], and vascular smooth muscle cells [37] are equipped with innate immune sensors, such as the caspase-1/inflammasome pathways for hyperlipidemia-related DAMPs [38]; (3) there are groups of homeostasis-associated molecular patterns (HAMPs) [27] that initiate signals counteracting innate immune/inflammatory signaling triggered by DAMPs/conditional DAMPs [39]; (4) as conditional antigen-presenting cells that upregulate costimulation receptors for T cell activation [33], activated endothelial cells can also act as immune tolerogenic cells and inflammation-suppressing cells by upregulating coinhibition receptors to impede T cell activation [40, 41]; (5) activated endothelial cells upregulate a set of circular RNAs for homeostasis [42, 43]; (6) upregulation of trained immunity (innate immune memory) pathways [44, 45] in aortic endothelial cells are new qualification markers for chronic disease risk factors and conditional DAMPs, which lead to enhanced innate immune responses in subsequently encountered risk factors [46]; and (7) in addition to inflammation initiation, we recently reported that suppression of inflammation and innate immunity was mediated by CD4^+^Foxp^3+^ regulatory T cells (Treg) [47–49] and the novel anti-inflammatory/immunosuppressive cytokine interleukin-35 (IL-35) [50–53].

Low-intensity ultrasound (LIUS) inhibits inflammation and innate immunity of noncancer immune cells and other cells [25, 54–56]. For example, ultrasound promotes vasodilation, enhances blood flow, promotes fibroblast and osteoblast proliferation, and increases other cellular components leading to wound healing [21]. Moreover, LIUS was reported to suppress synovial cell proliferation [57], affect mesenchymal stem cell migration [58], enhance the regeneration of myofibers [59], reduce the expression of inflammatory mediators [25], promote skin fibroblast proliferation [60], and promote chondrocyte and osteoblast proliferation [61]. By comparison, focused ultrasound induces thermal ablation, blood-brain barrier opening [62], and sterile inflammation [63]. To determine molecular mechanisms underlying LIUS suppression of inflammation and immunity, we recently reported that LIUS induces immunosuppression, which is mediated by several new mechanisms including gene induction, immunosuppressor cell promotion, and enhancement of exosome biogenesis and docking [2]. Moreover, we also reported that LIUS differentially upregulates cell death regulatome in cancer cells and downregulates inflammatory pathways in noncancer cells [64]. However, the detailed mechanisms underlying how LIUS regulates the expression of the complete innatome (all the regulators of innate immune immunity) [65] in cancer cells and noncancer cells remain poorly characterized. It also remains unclear how LIUS can distinguish cancer cells from noncancer cells and induce differential innate immune responses. Further, molecular mechanisms underlying LIUS-mediated inflammation inhibition and cancer-suppressing effects are not understood.

Coinhibition receptor pairs for T cells serve as immune checkpoints and are expressed by immune cells that play critical roles in maintaining immune homeostasis. Immune checkpoint inhibitors (ICPIs) are new cancer drugs that target self-tolerance pathways exploited by tumors to escape immune destruction, such as cytotoxic T-lymphocyte antigen 4 (CTLA-4) and programmed cell death 1 (PD-1) or its ligand (PD-L1). Of note, ICPI use can result in the development of many different inflammatory side effects, which are defined as immune-related adverse effects (irAEs) [66]. Our recent report determined the expression of 28 cosignaling receptors in 32 human tissues in physiological/pathological conditions and found that potential forward signaling for T cell inhibition and reverse signaling for antigen-presenting cell inhibition of 50% coinhibition receptors are upregulated in endothelial cells during inflammation [40]. These signs of progress suggest that in addition to ICPIs' T cell enhancement, other immune cells including endothelial cells as we proposed [33] activated by ICPIs including antigen-presenting cells may also significantly contribute to irARs via reverse signaling as we reported [40, 67]. However, an important question remains whether LIUS inhibition of inflammation at least partially is realized by suppression of costimulation receptors' signaling and enhancement of coinhibition receptor functions.

The adaptive immune system can develop antigen-specific memory T cells and B cells that have previously encountered and responded to their cognate antigens [46]. It was newly discovered that innate immune cells are also capable of developing an immune memory when exposed to certain inflammatory stimuli, and this type of memory, termed innate immune memory (trained immunity) [68], allows the development of enhanced responses when reencountering certain inflammatory stimuli. Three metabolic pathways (trained immunity pathways (TIP)) including the glycolysis pathway, the mevalonate pathway, and acetyl coenzyme A (acetyl-CoA) generation are responsible for initiating innate immune memory formation. These metabolic changes lead to the activation of the innate immune cells, altering their epigenetics, which serves as the sustained memory links between rewiring of cell metabolism and transcriptomic changes. These transcriptomic changes mimic those we recently reported in human aortic endothelial cells [46, 69]. However, another critical question remains whether LIUS inhibition of inflammation is partially contributed by its suppression of trained immunity (innate immune memory) pathways.

In order to broaden our understanding of LIUS-mediated immune modulation in the cellular context, we hypothesized that LIUS might induce differential innate immune gene expression patterns in cancer cells and noncancer cells. Therefore, in this study, we analyzed the expression patterns of a comprehensive list of 1376 innate immunity (innatomic) genes (IGs) [65] in LIUS-treated cancer cells and noncancer cells. We found that LIUS upregulates proinflammatory IGs and downregulates cancer metastasis genes in cancer cells. Also, LIUS has differential effects in suppressing danger signal sensing and inflammation initiation in bone marrow (BM) cells, and in enhancing IG expressions for adaptive immune responses in BM cells. Moreover, LIUS upregulates trained immunity enzymes in lymphoma cells but downregulates trained immunity enzymes in BM cells. Furthermore, coinhibition/immune checkpoint receptor (CI/ICR) B7-H4 overexpression promotes LIUS-upregulated IGs in lymphoma cells and LIUS-downregulated IGs in BM cells, while CI/ICR BTNL2 overexpression inhibits LIUS-upregulated IGs. Finally, we observed that the IGs modulated by LIUS in cancer cells and noncancer cells have unique chromatin long-range interaction (CLRI) sites. Chromatin looping enables CLRIs, which allows gene promoters to interact with distal regulatory elements [70]. The rapid development of technologies such as chromosome conformation capture-sequencing (3C-seq) [71], circularized chromosome conformation capture-sequencing (4C-seq) [72, 73], and chromosome conformation capture carbon copy-sequencing (5C-seq) [74] that capture chromosome conformation allows determination of interactions between the target genes and CLRI sites. The CLRI may enhance and modulate the expression of genes of interest. Differences in CLRI patterns between genes have previously been hypothesized to influence alternative splicing [75] and the transcription of inflammatory genes, such as cytokine [76], cytokine receptor [77], and cardiovascular disease-causative genes [78]. Therefore, we suggest that the unique CLRI seen in genes modulated by LIUS in cancer cells and noncancer cells may play a role in producing a differential response.

## 2. Materials and Methods

### 2.1. Expression Profile of Cell Death Genes in Ultrasound-Treated, Mild Hyperthermia-Treated, and Oscillatory Shear Stress-Treated Cells

Microarray datasets were collected from the National Institutes of Health- (NIH-) National Center for Biotechnology Information (NCBI) GEO DataSet (https://www.ncbi.nlm.nih.gov/gds/) database and analyzed with GEO2R (https://www.ncbi.nlm.nih.gov/geo/geo2r/). In the first part of GEO DataSets are the microarray data sets from three LIUS-treated, two mild hyperthermia-treated, one oscillatory shear stress-treated cells as follows: GSE10212, GSE45487, GSE70662, GSE10043, GSE39178, and GSE60152. The detailed information of these GEO DataSets appears in Table 1(a). GEO DataSet IDs in other parts of the manuscripts are listed in their respective tables and figures.

In this report, 1376 innatomic genes (IGs)∗ were studied, which is collected from the InnateDB [65]. As shown in Table 2, the updated gene list at http://www.innatedb.com provides details of the IGs, which have been annotated by either InnateDB or Gene Ontology as having a role in the innate immune response, and is updated weekly.

### 2.2. Statistical Analysis of Microarray Data

As we reported [46, 79], we applied a similar statistical method utilizing the expression of four housekeeping genes (CHMP2A, PSMB4, ACTB, and GAPDH) [80]. Briefly, variation of housekeeping gene expressions between treatment and control groups varied narrowly from -1.27 to 1.28, providing confidence in the quality of the chosen GEO DataSets used here. The target genes with expression that changed more than 1.5-fold were defined as the upregulated genes, while genes that decreased more than 1.5-fold were defined as downregulated genes.

### 2.3. Ingenuity Pathway Analyses and Venn Diagram Analyses

We utilized Ingenuity Pathway Analysis (IPA, Ingenuity Systems, https://www.qiagenbioinformatics.com/products/ingenuity-pathway-analysis/) to characterize the clinical relevance and molecular and cellular functions related to the identified genes in our microarray analysis. The differentially expressed genes were identified and uploaded into IPA for analysis. The Core and Pathway Analysis was used to identify molecular and cellular pathways as we have previously reported [79, 81]. In addition, Venn diagram analyses (http://bioinformatics.psb.ugent.be/webtools/Venn/) were used to determine both shared genes/signaling pathways and cell-specific genes/pathways.

### 2.4. Chromatin Long-Range Interaction Analysis

The chromatin long-range interaction data were collected from the Hi-C data deposited in the 4D Genome database (https://4dgenome.research.chop.edu) as a tabulated text file [82]. Interacting gene and CLRI sites relating to LIUS-regulated cell death genes were filtered. The distance between interaction sites and LIUS-modulated gene promotors was then calculated [42, 46]. In brief, the filtered data was imported into Microsoft Excel, raw distance between interaction sites was calculated according to the gene start site, and an AWK script was written to tabulate whether the interacting site is upstream (+) or downstream (-) of the affected gene as we reported [42]. Distance distributions for all upregulated and all downregulated LIUS-modulated genes were compared by groups.

## 3. Results

### 3.1. LIUS Upregulates Proinflammatory Innatomic Genes (IGs) and Downregulates Cancer Metastasis Genes in Cancer Cells

As outlined in the Introduction, low-intensity ultrasound (LIUS) inhibits inflammation and innate immunity by various cellular mechanisms in noncancer immune cells and other cells [25, 54–56]. As we pointed out in our recent LIUS papers [2, 64], many publications reported that LIUS induces cell death pathways in cancer cells. In contrast, LIUS exerts other therapeutic effects in noncancer cells such as modulation of cell proliferation, regulation of cell migration, and enhancement of regeneration. However, an important question remained whether cancer cells and noncancer cells produce differential innate immune responses to LIUS [83]. We hypothesized that LIUS induces differential innate immune responses in cancer cells and noncancer cells by modulating the expressions of a comprehensive list of innate immune regulators (innatome genes (IGs)) [84]. A list of these IGs are reported in Table 2. In addition, we found three microarray datasets deposited in the NIH-NCBI GEO DataSet repository, which depicted human lymphoma cells and noncancer mouse MC3T3-E1 preosteoblast cells and rat BM cells that were treated with LIUS (Table 1).

**Figure 1:**
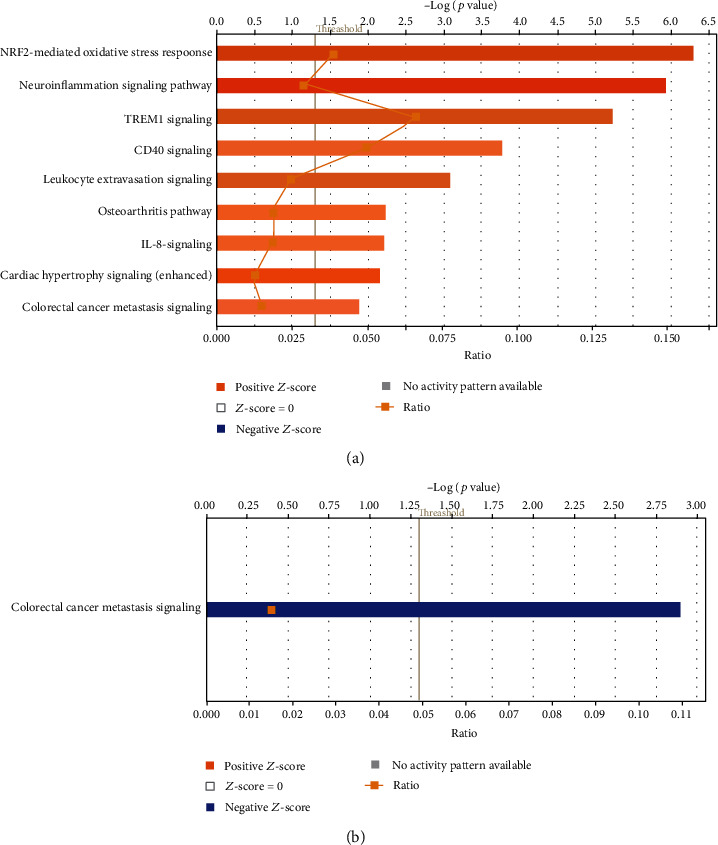
(a) Low-intensity ultrasound (LIUS) upregulated 77 out of 1376 (5.6%) innatomic genes and downregulated 39 out of 1376 (2.8%) innatomic genes (IIGs) in human lymphoma U937 cells (GSE10212), suggesting that (1) LIUS increases innatomic gene expressions more than it decreases them in cancer cells in human lymphoma cells, and (2) upregulation of innatomic genes in lymphoma cells serves as a novel immune mechanism underlying antitumor effects of LIUS (see supplemental Table 1 for the detailed gene list. Of note, due to the big data we generated, we have to summarize and present the key findings in this type unconventional format as we previous reported; PMID: 29434588). LIUS upregulated 77 genes that were significantly involved in nine signaling pathways in human lymphoma cells, which included NRF2-mediated oxidative stress response, neuroinflammation signaling, TREM1 signaling, CD40 signaling, leukocyte extravasation signaling, osteoarthritis pathway, IL-8 signaling, cardiac hypertrophy signaling, and cancer metastasis signaling (PMID: 31315034). Of note, eight out of nine pathways were proinflammatory pathways except NRF2 (anti-inflammatory, PMID: 27825853). (b) LIUS downregulated 39 genes that were significantly involved in only one pathway (cancer metastasis signaling) in human lymphoma cells, suggesting that LIUS inhibits inflammation-driven cancer metastasis (PMID: 31315034).

As shown in Figure 1(a), among the 1376 genes analyzed, low-intensity ultrasound (LIUS) upregulated 77 IGs (5.6%) and downregulated 39 IGs (2.8%) in human lymphoma cells, suggesting that (1) LIUS increases IG expressions more than decreasing them in human lymphoma cells, and (2) upregulation of IGs in lymphoma cells serves as a novel immune mechanism underlying antitumor effects of LIUS. The Ingenuity Pathway Analysis (IPA) on upregulated and downregulated genes by LIUS treatment in cancer cells (Figures 1(a) and 1(b)) revealed that 77 LIUS-upregulated genes were significantly involved in nine signaling pathways in human lymphoma cells, which included nuclear factor erythroid 2-related factor 2- (NRF2-) mediated oxidative stress response, neuroinflammation signaling, triggering receptor expressed on myeloid cells 1 (TREM1) signaling, CD40 signaling, leukocyte extravasation signaling, osteoarthritis pathway, interleukin-8 (IL-8) signaling, cardiac hypertrophy signaling, and cancer metastasis signaling [85]. Of note, eight out of the top nine pathways were proinflammatory pathways except NRF2 (anti-inflammatory) [86]. In addition, 39 LIUS-downregulated genes were significantly involved in only one pathway (cancer metastasis signaling) in human lymphoma cells, suggesting that LIUS inhibits inflammation-driven cancer metastasis [85]. Of note, cancer metastasis signaling was shown in both LIUS-upregulated pathways and LIUS-downregulated pathways. These results suggest that LIUS upregulates one subgroup of cancer metastasis genes and downregulates another subgroup of cancer metastasis genes. These results also suggest that LIUS upregulates proinflammatory IGs and downregulates cancer metastasis genes in cancer cells.

### 3.2. LIUS Has Differential Effects in Suppressing DAMP-Sensing and Inflammation Initiation Pathways in Bone Marrow (BM) Enhancing Innatome Support for Adaptive Immune Responses in BM

**Figure 2:**
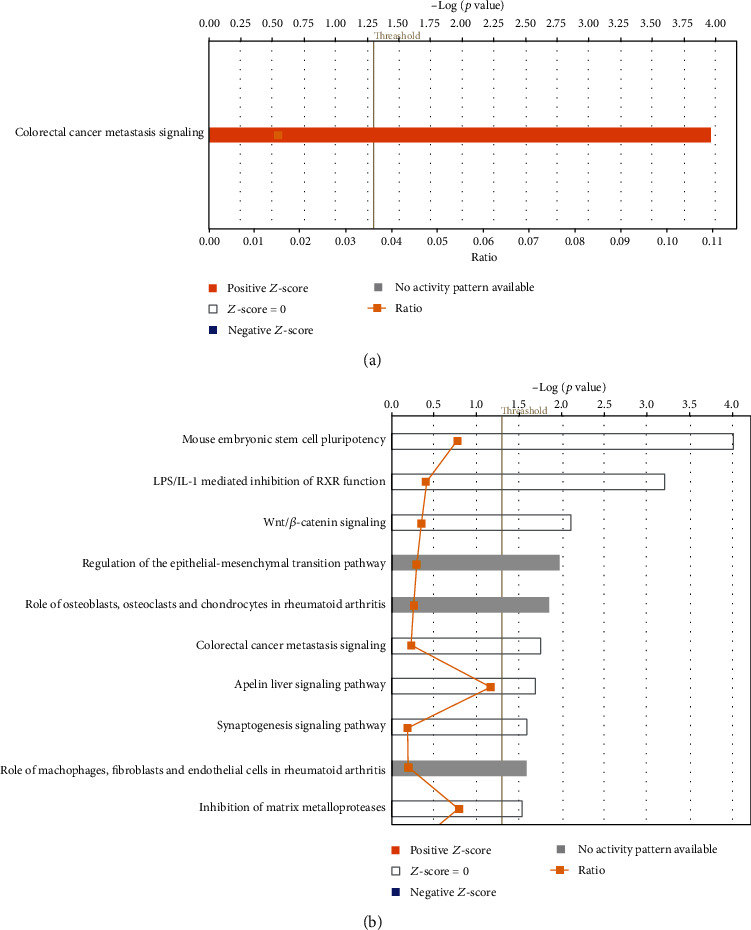
(a) Low-intensity ultrasound (LIUS) upregulates 21 out of 1376 (1.5%) innatomic genes and downregulates 17 out of 1376 (1.2%) innatomic genes in mouse preosteoblast cells (GSE45487), suggesting that LIUS increases innatomic gene expressions slightly more than decreasing them in mouse preosteoblast cells (see supplemental Table 2 for the detailed gene list). LIUS upregulated 21 innatomic genes that were significantly involved in one signaling pathway in mouse preosteoblasts, which is inflammation-driven cancer metastasis signaling. (b) LIUS downregulated 17 innatomic genes that were not significantly involved in any signaling pathway in mouse preosteoblast cells, suggesting that LIUS inhibits innatome in preosteoblasts in multipathways in a nonsignificant manner.

We hypothesized that LIUS inhibits inflammation-promoting IGs in noncancer cells. As shown in Figure 2(a), LIUS upregulated 21 out of 1376 (1.5%) IGs and downregulated 17 out of 1376 (1.2%) IGs in mouse preosteoblast cells (GSE45487), suggesting that LIUS increases IG expressions slightly more than decreasing them in mouse preosteoblast cells. Previous reports showed that patients, following organ transplantation and being treated with inhibitors of the calcineurin/NFATc1 pathway, such as cyclosporin A and FK506, often develop osteoporosis [87, 88], suggesting that immunosuppression and anti-inflammation therapies inhibit bone formation. When conducting IPA on upregulated and downregulated genes by LIUS treatment in mouse preosteoblast cells, it revealed that 21 LIUS-upregulated genes were significantly involved in one pathway, cancer metastasis signaling (Figure 2(a)). On the other hand, 17 LIUS-downregulated IGs were not significantly involved in any signaling pathways in mouse preosteoblast cells (Figure 2(b)).

**Figure 3:**
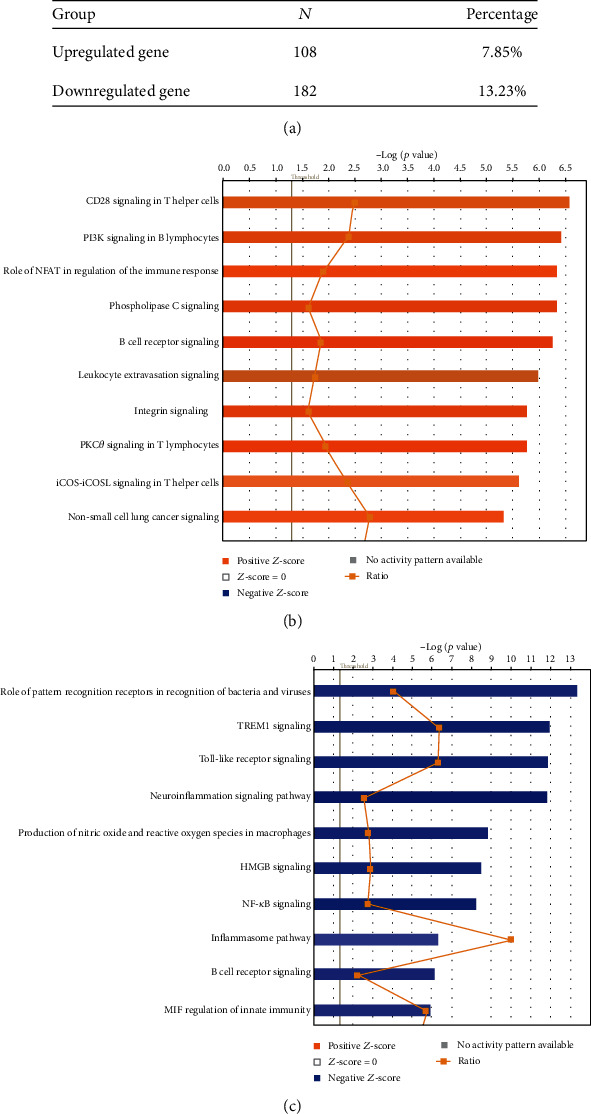
(a) Low-intensity ultrasound (LIUS) upregulated 108 out of 1376 (7.9%) innate immunome genes and downregulated 182 out of 1376 (13.2%) innate immunomic genes in rat bone marrow cells (GSE70662), suggesting that LIUS suppresses innate immunomic gene expressions more than it increases them in bone marrow cells. The LIUS-modulated genes are listed in Supplemental Table 3. (b) LIUS upregulated 108 genes that were significantly involved in 54 signaling pathways in bone marrow cells. The top ten pathways include CD28 signaling in T cells, PI3K signaling in B lymphocytes, the role of NFAT in regulation of immune responses, phospholipase C signaling, B cell receptor signaling, leukocyte extravasation signaling, integrin signaling, PKC zeta signaling in T lymphocytes, ICOS-ICOSL signaling in T helper cells, and non-small-cell lung cancer signaling. (c) LIUS downregulated 182 genes that were significantly involved in 70 signaling pathways in bone marrow cells. The top ten pathways include the role of pattern recognition receptors, TREM1 signaling, Toll-like receptor signaling, neuroinflammation signaling, production of nitric oxide and reactive oxygen species (ROS) in macrophages, HMGB1 signaling, NF-*κ*B signaling, inflammation pathway, B cell receptor signaling, and MIF regulation of innate immunity. These results suggest that LIUS inhibits numerous key innate immunity and inflammation pathways in bone marrow cells, which may be responsible for LIUS therapeutic effects of inflammation suppression since nine out of the top ten pathways are related to danger signal recognition and inflammation initiation.

A recent report showed that hematopoietic progenitor cells are integrative hubs for adaptation to and fine-tuning of inflammation [89]. The inflammation-induced adaptation of hematopoietic and myeloid progenitor cells toward enhanced myelopoiesis might also perpetuate inflammation in chronic inflammatory or cardiometabolic diseases by generating a feed-forward loop between inflammation-adapted hematopoietic progenitor cells and the inflammatory disorder [89]. We hypothesized that LIUS inhibits inflammation-promoting IGs in noncancer BM cells. As shown in Figure 3(a), LIUS upregulated 108 out of 1376 (7.9%) IGs, and downregulated 182 out of 1376 (13.2%) IGs in rat BM cells (GSE70662), suggesting that LIUS suppresses IG expression more than increasing them in BM cells; and the effects of LIUS on BM cells are the most significant responses among three cell types. Of note, future experiments will be needed to reexamine this issue with cell types from the same species. The LIUS-modulated genes were listed in Supplemental Table S3. When IPA was conducted on upregulated and downregulated genes resulting from LIUS treatment in BM cells, it revealed that 108 LIUS-upregulated genes are significantly involved in 54 signaling pathways in BM cells (Figures 3(a) and 3(b)). The top ten pathways included CD28 signaling in T cells, phosphoinositide 3-kinase (PI3K) signaling in B lymphocytes, the role of nuclear factor of activated T cells (NFAT) in regulation of immune responses, phospholipase C signaling, B cell receptor signaling, leukocyte extravasation signaling, integrin signaling, protein kinase C (PKC) zeta signaling in T lymphocytes, inducible T cell costimulator- (ICOS-) inducible T cell costimulator ligand (ICOSL) signaling in T helper cells, and non-small-cell lung cancer signaling. Six out of the top ten pathways listed are related to adaptive immune response. This demonstrates for the first time that LIUS suppression of inflammation and innate immunity in BM cells requires the participation of numerous key signaling pathways of adaptive immune response in T helper cells and B cells.

As shown in Figure 3(c), the 182 LIUS-downregulated genes are significantly involved in 70 signaling pathways in BM cells. The top ten pathways include the role of pattern recognition receptors, TREM1 signaling, Toll-like receptor signaling, neuroinflammation signaling, production of nitric oxide and reactive oxygen species in macrophages, high-mobility group protein B1 (HMGB1) signaling, NF-*κ*B signaling, inflammation pathway, B cell receptor signaling, and MIF regulation of innate immunity. Since nine out of the top ten pathways are related to danger signal recognition and inflammation initiation, these results suggest that LIUS inhibits numerous key innate immunity and inflammation pathways in BM cells, which may be responsible for LIUS's therapeutic effects of inflammation suppression. To find a supportive report to demonstrate whether the antigen-presenting innate immune function can ever be separated from the inflammatory function, we indeed found a recent Science report using a new single-cell RNA sequencing technique to separate the antigen-presenting major histocompatibility complex class II- (MHC-II-) high dendritic cell (DC) population from the inflammatory function-high DC population [90]. Our results have demonstrated for the first time that LIUS has differential effects in suppressing danger signal sensing and recognition, on inflammation initiation in BM cells, and in enhancing IG expression for supporting adaptive immune responses in BM cells.

**Figure 4:**
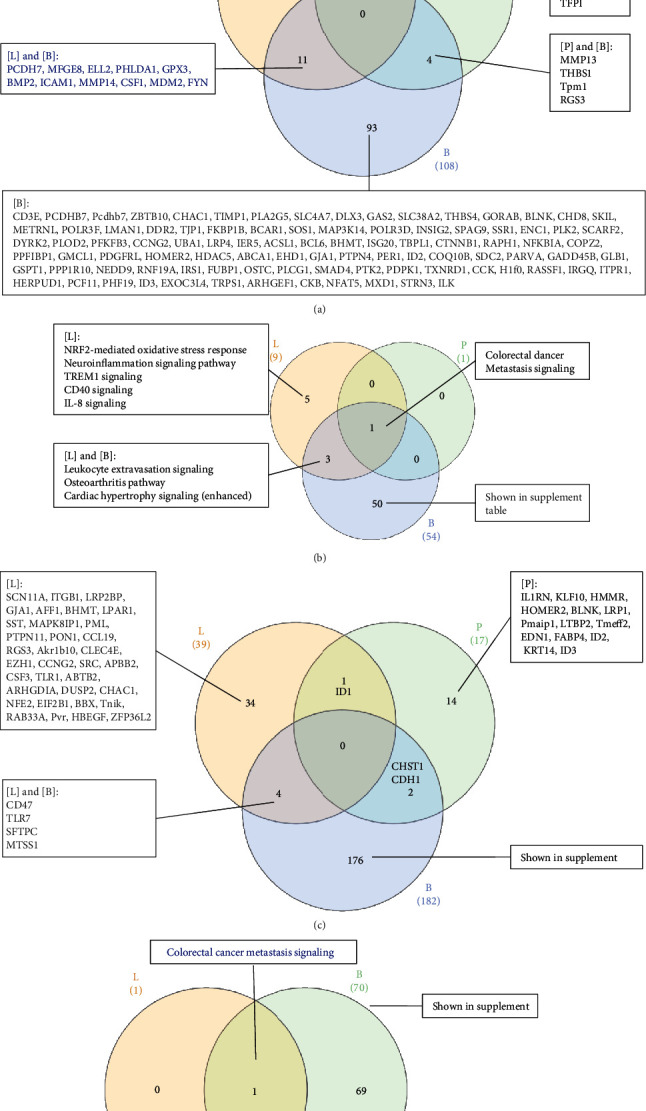
(a) The Venn Diagram analyses shows that LIUS-upregulated innatomic genes in three cell types are partially shared. The three genes shared by lymphoma cells (L) and preosteoblasts (P) and the 11 genes shared by lymphoma cells (L) and bone marrow cells (B) may be used for LIUS therapeutic markers. However, the majority of LIUS-upregulated innatomic genes are cell type specific. (b) The Venn Diagram analyses shows that the signaling pathways involved in LIUS-upregulated innatomic genes in three cell types are partially shared, such as metastasis signaling. In addition, three pathways are shared by LIUS-treated lymphoma cells and bone marrow cells including leukocyte extravasation, osteoarthritis pathway, and cardiac hypertrophy signaling. However, the majority of LIUS-upregulated innatomic pathways in lymphoma and bone marrow cells are cell type specific. (c) The Venn Diagram analyses shows that LIUS-downregulated innatomic genes in three cell types are not shared. The majority of LIUS-downregulated innatomic genes are cell type specific (L: lymphoma cells; B: bone marrow cells; P: preosteoblasts). (d) The Venn Diagram analysis shows that the signaling pathways involved in LIUS-downregulated innatomic genes in lymphoma cells and bone marrow cells are partially shared, such as metastasis signaling. However, the majority of LIUS-downregulated innatomic pathways in bone marrow cells are cell type specific.

As shown in Figure 4(a), the Venn diagram analysis showed that LIUS-upregulated IGs in three cell types are partially shared. The three genes shared by lymphoma cells and preosteoblasts and the 11 genes shared by lymphoma and BM cells may be used for LIUS therapeutic markers. However, the majority of LIUS-upregulated IGs were cell type specific. As shown in Figure 4(b), the Venn diagram analysis showed that the signaling pathways involved in LIUS-upregulated innatomic genes in the three cell types are partially shared, such as cancer metastasis signaling. In addition, three pathways were shared by LIUS-upregulated IGs in lymphoma cells and BM cells, including three inflammation-related signaling pathways (leukocyte extravasation, osteoarthritis pathway, and cardiac hypertrophy signaling). However, the majority of LIUS-upregulated IG pathways in lymphoma and BM cells were cell type specific. As shown in Figure 4(c), the Venn diagram analysis showed that LIUS-downregulated IGs in three cell types are not shared. The majority of LIUS-downregulated IGs were cell type specific. As shown in Figure 4(d), the Venn diagram analysis shows that the signaling pathways involved in LIUS-downregulated IGs in lymphoma cells and BM cells are partially shared, such as cancer metastasis signaling. In both lymphoma cells and BM cells, LIUS upregulated one subgroup of cancer metastasis genes and downregulated another subgroup of cancer metastasis genes. However, the majority of LIUS-downregulated IG pathways in BM cells were cell type specific. These results have demonstrated for the first time that LIUS has differential effects in upregulating IGs for supporting adaptive immune responses and inhibiting proinflammatory regulators in noncancer BM cells. Our previous report showed that [1] LIUS's anti-inflammatory effects are mediated by upregulating anti-inflammatory gene expression, and [2] LIUS induces the upregulation of the markers and master regulators of a group of immunosuppressor cells including myeloid-derived suppressor cells (MDSCs), mesenchymal stem cells (MSCs), B1 B cells, and CD4^+^Foxp3^+^ regulatory T cells (Treg) [2]. Therefore, one explanation of our new results is that LIUS upregulates IGs to make bone marrow-derived cells for Treg-related immunosuppressive adaptive immune responses [40, 47–49].

### 3.3. LIUS Differentially Upregulates More IGs Encoded for Proteins in the Cytoplasm, Extracellular Space, and Others

As shown in Table 2, based on subcellular localization of IGs, five subgroups were classified including the cytoplasm, extracellular space, nucleus, and plasma membrane. In addition, based on functions of IGs, 14 subgroups were classified including cytokines, enzymes, G-protein-coupled receptors, growth factors, ion channels, kinases, ligand-dependent nuclear receptors, others, peptidases, phosphatases, transcription factors, translational regulators, transmembrane receptors, and transporters. We previously reported that LIUS upregulates the expression of extracellular vesicle/exosome biogenesis mediators and docking mediators, suggesting that LIUS has specific effects on the biogenesis of certain subcellular organelles [2]. Thus, we hypothesized that LIUS differentially modulates the expression of IGs in a subcellular localization-dependent manner. As shown in Table 3(a), IPA showed that three out of five subcellular localization groups (cytoplasm, extracellular space, and others) of LIUS-upregulated IGs are significantly changed in lymphoma cells, preosteoblast cells, and BM cells. However, none of the 14 functional subgroups of LIUS-upregulated innatomic genes in these three cell types were changed, suggesting that LIUS-upregulated IGs have global effects on the cell transcriptome regardless of functional subgroups. In addition, as displayed in Table 3(b), IPA showed that two out of five subcellular localization groups (nucleus and plasma membrane) of LIUS-downregulated IGs in lymphoma cells, preosteoblasts, and BM cells are significantly changed. However, one of the 14 functional groups (phosphatase) of IGs was also significantly downregulated from 1.6% in the general innatome to 1.3% in lymphoma cells and 0.93% in BM cells but was not changed in preosteoblast cells.

Taken together, these results have demonstrated that first, LIUS differentially upregulates more IGs encoded for proteins localized in three out of five subcellular locations such as the cytoplasm, extracellular space, and other subcellular localizations, but downregulates more IGs encoded for proteins localized in the nucleus and plasma membrane subcellular locations, suggesting that LIUS has specific effects on different subcellular localized innatome proteins; second, LIUS downregulates more phosphatases than the other 13 functional subgroups; and third, since downregulation of phosphatases appear to be a consequence of LIUS treatment, downregulation of phosphatases may serve as a clinical efficacy marker for LIUS therapies. Our results are well correlated with previous reports showing that proinflammatory protein phosphatase 2A (PP2A) can be targeted for anticancer and anti-inflammatory drugs [91], and that proinflammatory protein phosphatase 6 can also be targeted [92].

### 3.4. LIUS Modulates IGs Partially via Static or Oscillatory Shear Stress Mechanisms and Heat-Generated Mechanisms

**Figure 5:**
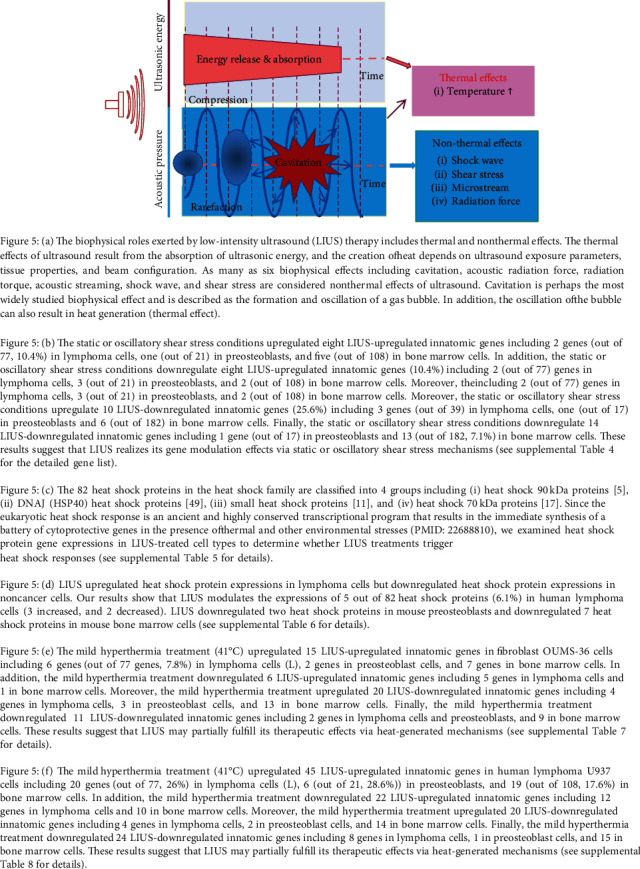


**Figure 6:**
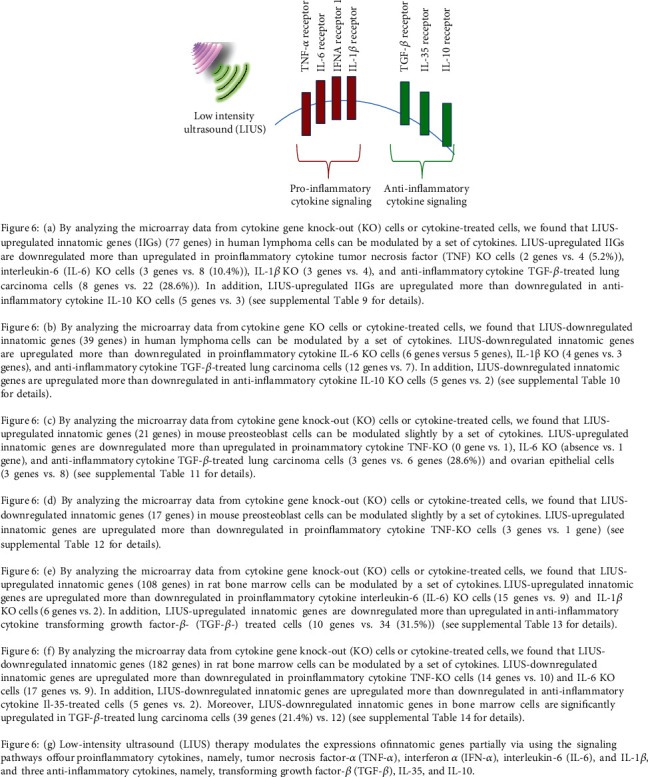


We and others reported that the biophysical roles exerted by LIUS therapy include thermal and nonthermal effects (Figure 5) [2, 64]. The thermal effects of ultrasound result from the absorption of ultrasonic energy, and the creation of heat depends on ultrasound exposure parameters, tissue properties, and beam configuration. As many as six biophysical effects, including cavitation, acoustic radiation force, radiation torque, acoustic streaming, shock wave, and shear stress, are considered nonthermal effects of ultrasound [7], although there are differing opinions on the classification of cavitation [7, 93, 94]. LIUS is a form of ultrasound that delivers ultrasonic energy at a much lower intensity (<3 W/cm^2^) than high-intensity focused ultrasound, and it has been considered as a removed thermal component or having minimal thermal effects due to its low-intensity mode [95, 96]. Cavitation is perhaps the most widely studied biophysical effect and is described as the formation and oscillation of a gas bubble. In addition, the oscillation of the bubble can result in heat generation (thermal effect) [64]. We hypothesized that LIUS partially fulfills its therapeutic effects via static or oscillatory shear stress mechanisms and heat-generated mechanisms. To examine this hypothesis, we identified a few microarray datasets collected from cells treated with static or oscillatory shear stress and thermal stress in the NIH-NCBI GEO DataSet repository (Table 1(a)) to determine whether the expression of LIUS-upregulated and LIUS-downregulated IGs would be changed in those biophysical stress-treated cells. Of note, due to the space limits, we presented the highlights from a large amount of high-throughput data analytic results in unconventional figure formats and presented the detailed results in the Supplemental Tables linked with figures, which were similar to what we reported previously [49, 97]. As shown in Figure 6(b), the static or oscillatory shear stress conditions upregulated eight LIUS-upregulated IGs including two genes (out of 77) in lymphoma cells, one gene (out of 21) in preosteoblasts, and five genes (out of 108) in BM cells. In addition, the static or oscillatory shear stress conditions downregulated eight LIUS-upregulated IGs including two genes (out of 77) in lymphoma cells, three genes (out of 21) in preosteoblasts, and two genes (out of 108) in BM cells. Moreover, the static or oscillatory shear stress conditions upregulated ten LIUS-downregulated IGs including three genes (out of 39) in lymphoma cells, one gene (out of 17) in preosteoblasts, and six genes (out of 182) in BM cells. Finally, the static or oscillatory shear stress conditions downregulated 14 LIUS-downregulated IGs including one gene (out of 17) in preosteoblasts, and 13 genes (out of 182) in BM cells (none in lymphoma cells). These results suggest that LIUS may partially fulfill its therapeutic effects via static or oscillatory shear stress mechanisms and that LIUS uses more static or oscillatory shear stress mechanisms in noncancer BM cells than lymphoma cells.

The eukaryotic heat shock response is an ancient and highly conserved transcriptional program that results in the immediate synthesis of a battery of cytoprotective genes in the presence of thermal and other environmental stresses [98]. Some publications have reported an increase in temperature of approximately 3 to 4°C after LIUS treatment [99], meaning that the thermal effect is inevitable during LIUS treatment. Thus, to determine whether LIUS treatments triggered heat shock responses by modulating the expression of heat shock proteins in the cells where the microarray experiments were performed, we examined heat shock protein gene expression in the three LIUS-treated cell types. The 82 heat shock proteins in the whole heat shock family as shown in Figure 6(c) were classified into four groups including heat shock 90 kDa proteins, DNAJ (HSP40) heat shock proteins [49], small heat shock proteins [11], and heat shock 70 kDa proteins [17]. As shown in Figure 6(d), LIUS upregulated heat shock protein expression in lymphoma cells but heat shock proteins were not significantly modulated in the other two LIUS-treated microarray datasets. Our results showed that LIUS modulated the expression of five out of 82 heat shock proteins (6.1%) in human lymphoma cells (three increased, and two decreased). LIUS either downregulated or insignificantly modulated, two and seven heat shock proteins in mouse preosteoblasts and mouse BM cells, respectively. These results also suggest that LIUS-induced heat shock responses are different in cancer cells and noncancer cells, which were related to the LIUS parameters used (Table 1(a)).

We further determined whether mild hyperthermia treatment modulates LIUS-upregulated and downregulated IGs. As shown in Figure 6(e), the mild hyperthermia treatment (41°C) upregulated 15 LIUS-upregulated IGs in fibroblast OUMS-36 cells including six genes in lymphoma cells (L), two genes in preosteoblast cells, and seven genes in BM cells. In addition, the mild hyperthermia treatment downregulated six LIUS-upregulated IGs including five genes in lymphoma cells, and one gene in BM cells. Moreover, the mild hyperthermia treatment upregulated 20 LIUS-downregulated IGs including four genes in lymphoma cells, three genes in preosteoblast cells, and 13 genes in BM cells. Finally, the mild hyperthermia treatment downregulated 11 LIUS-downregulated IGs including two genes in lymphoma cells and preosteoblasts and nine genes in BM cells. These results suggest that LIUS may partially fulfill its therapeutic effects via heat-generated mechanisms.

As shown in Figure 6(f), the second mild hyperthermia treatment (41°C) upregulated 45 LIUS-upregulated IGs in human lymphoma U937 cells including 20 genes (out of 77, 26%) in lymphoma cells (L), six genes (out of 21, 28.6%) in preosteoblasts, and 19 genes (out of 108, 17.6%) in BM cells. In addition, the mild hyperthermia treatment downregulated 22 LIUS-upregulated IGs including 12 genes in lymphoma cells and ten genes in BM cells. Moreover, the mild hyperthermia treatment upregulated 20 LIUS-downregulated IGs including four genes in lymphoma cells, two genes in preosteoblast cells, and 14 genes in BM cells. Finally, the mild hyperthermia treatment downregulated 24 LIUS-downregulated IGs including eight (out of 39, 20.5%) genes in lymphoma cells, one gene (out of 17, 5.9%) in preosteoblast cells, and 15 genes (out of 182, 8.2%) in BM cells. These results suggest that LIUS may partially fulfill its therapeutic effects via heat-generated mechanisms.

Taken together, LIUS modulated IGs partially via static or oscillatory shear stress mechanisms and heat-generated mechanisms.

### 3.5. LIUS-Modulated IGs Are Regulated Partially by Inflammatory Cytokines

As we reported previously, cytokines play significant roles in modulating inflammation responses [26]. Thus, we hypothesized that LIUS-modulated IGs can also be regulated partially by mediation through inflammatory cytokine signals. To examine this hypothesis, we found eight microarray datasets from cytokine/cytokine receptor knock-out (KO, gene deficient) cells or cytokine-treated cells in the NIH-NCBI GEO DataSet repository including four proinflammatory cytokine datasets, namely, tumor necrosis factor-*α* (TNF-*α*) KO, interferon-*α* receptor 1 (IFNAR1) KO, interleukin-6 (IL-6) KO, and IL-1*β* KO, and four anti-inflammatory cytokine datasets, including IL-10 KO, IL-35-treated, and two transforming growth factor-*β*- (TGF-*β*-) treated cell datasets. By analyzing the microarray data from cytokine/cytokine receptor gene knock-out (KO) cells or cytokine-treated cells, we found that LIUS-upregulated innatomic genes in human lymphoma cells can be modulated by a set of cytokines. LIUS-upregulated IGs were downregulated more than upregulated in proinflammatory cytokine TNF-*α* KO cells, IL-6 KO cells, IL-1*β* KO, and anti-inflammatory cytokine TGF-*β*-treated lung carcinoma cells. In addition, LIUS-upregulated IGs were upregulated more than downregulated in anti-inflammatory cytokine IL-10 KO cells. These results suggest that LIUS treatment of cancer cells promotes IG expression by enhancing proinflammatory cytokine pathways and inhibiting anti-inflammatory cytokine pathways. In addition, as shown in Figure 5, by analyzing the microarray data from cytokine/cytokine receptor gene KO cells or cytokine-treated cells, we found that LIUS-downregulated IGs in human lymphoma cells can be modulated by a set of cytokines. LIUS-downregulated innatomic genes were upregulated more than downregulated in proinflammatory cytokine IL-6 KO cells, IL-1*β* KO, and anti-inflammatory cytokine TGF-*β*-treated lung carcinoma cells. In addition, LIUS-downregulated IGs were upregulated more than downregulated in anti-inflammatory cytokine IL-10 KO cells. Once again, these results suggest that LIUS treatment of cancer cells promotes IG expression by enhancing proinflammatory cytokine pathways and inhibiting anti-inflammatory cytokine pathways.

By analyzing the microarray data from cytokine/cytokine receptor KO cells or cytokine-treated cells, we found that LIUS-upregulated IGs in mouse preosteoblast cells can be modulated slightly by a set of cytokines. LIUS-upregulated IGs were downregulated more than upregulated in proinflammatory cytokine TNF-*α* KO, IL-6 KO, and anti-inflammatory cytokine TGF-*β*-treated lung carcinoma cells and ovarian epithelial cells. These results suggest that LIUS treatment of mouse preosteoblast cells slightly promotes IG expression by enhancing proinflammatory cytokine pathways and inhibiting anti-inflammatory cytokine pathways. By analyzing the microarray data from cytokine/cytokine receptor KO cells or cytokine-treated cells, we found that LIUS-downregulated IGs in mouse preosteoblast cells can be modulated slightly by a set of cytokines. LIUS-upregulated IGs were upregulated more than downregulated in proinflammatory cytokine TNF-KO cells. These results suggest that LIUS treatment of mouse preosteoblast cells slightly promotes IG expression by enhancing proinflammatory cytokine pathways and inhibiting anti-inflammatory cytokine pathways.

By analyzing the microarray data from cytokine/cytokine receptor KO cells or cytokine-treated cells, we found that LIUS-upregulated IGs in rat BM cells can be modulated by a set of cytokines. LIUS-upregulated IGs were upregulated more than downregulated in proinflammatory cytokine IL-6 KO cells and IL-1*β* KO cells. In addition, LIUS-upregulated innatomic genes were downregulated more than upregulated in anti-inflammatory cytokine TGF-*β*-treated cells. These results suggest that LIUS treatment of BM cells suppresses IG expression and inflammation by inhibiting proinflammatory cytokine pathways and enhancing anti-inflammatory cytokine pathways. By analyzing the microarray data from cytokine/cytokine receptor KO cells or cytokine-treated cells, we found that LIUS-downregulated IGs in rat BM cells can be modulated by a set of cytokines. LIUS-upregulated IGs were upregulated more than downregulated in proinflammatory cytokine TNF-*α* KO cells and IL-6 KO cells. In addition, LIUS-downregulated IGs were upregulated more than downregulated in anti-inflammatory cytokine IL-10 KO and IL-35 KO cells. Moreover, LIUS-downregulated IGs in BM cells are significantly upregulated in TGF-*β*-treated lung carcinoma cells. These results suggest that LIUS treatment of BM cells suppresses IG expression and inflammation by inhibiting proinflammatory cytokine pathways and enhancing anti-inflammatory cytokine pathways.

### 3.6. LIUS Upregulates Trained Immunity Enzymes in Lymphoma Cells, Downregulates Trained Immunity Enzymes, and Establishes Trained Tolerance in BM Cells

**Figure 7:**
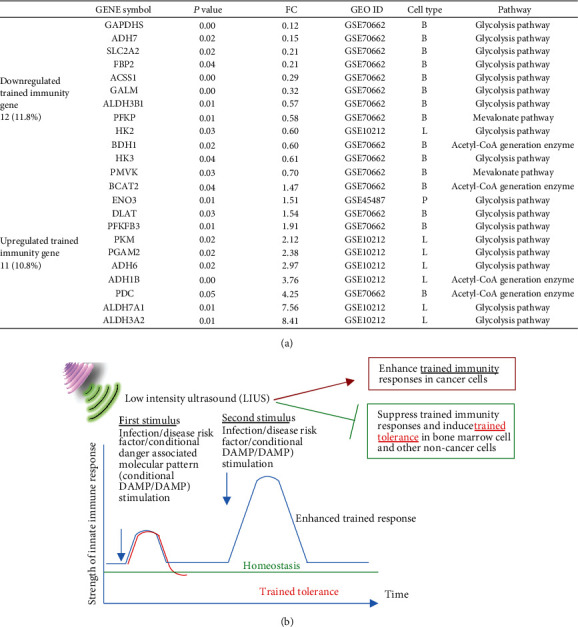
(a) LIUS upregulated trained immunity enzymes in lymphoma cells and downregulated trained immunity enzymes in bone marrow cells, suggesting that LIUS enhances innate immune responses in cancer cells and inhibits innate immune responses in noncancer cells, which are associated with its modulation of trained immunity enzyme expressions. By analyzing microarray data of LIUS-treated human lymphoma cells, mouse preosteoblasts, and rat bone marrow cells, we examined a novel hypothesis that LIUS induces its therapeutic effects in cells by modulating the expressions of our newly reported (PMID: 31153039) innate immune memory (trained immunity) pathway enzymes. Our results show that LIUS downregulates 12 out of 102 trained immunity genes, including 11 (10.8%) in bone marrow cells and one in lymphoma cells. In addition, LIUS induces 11 out of 102 trained immunity genes (10.8%) including 6 in lymphoma cells, 1 in preosteoblasts, and 4 in bone marrow cells. Moreover, in bone marrow cells, LIUS upregulates 4 trained immunity genes and downregulates 11 trained immunity genes including 8 glycolysis enzymes, one acetyl-CoA enzyme, and two mevalonate pathway enzymes, suggesting that 2.75-fold more downregulation than upregulation of trained immunity genes in bone marrow cells contributes significantly to LIUS-suppressed innate immunity and inflammation. Finally, in contrast, in lymphoma cells, LIUS upregulation of six trained immunity enzymes including 5 glycolysis enzymes and one acetyl-CoA generation enzymes and downregulation of one glycolysis enzyme in lymphoma cells (6-fold more upregulation than downregulation) contribute significantly to LIUS-induced innate immunity enhancement. (b) Low-intensity ultrasound (LIUS) therapy upregulated trained immunity enzymes (PMID: 27102489) in lymphoma cells and downregulated trained immunity enzymes in bone marrow cells. These results suggest that LIUS enhances antitumor immune responses against lymphoma cells at least partially by upregulating trained immunity pathways, which is similar to that proposed by others (PMID: 27903713); LIUS also inhibits inflammation in noncancer cells by suppressing trained immunity pathways (see our recent report, PMID: 31153039). Trained immunity (innate immune memory): newly characterized adaptive metabolic and epigenetic remodeling of innate immune cells including (1) upregulation of glycolysis pathway enzymes, (2) increased acetyl-CoA generation, (3) upregulation of mevalonate pathway enzymes, and (4) remodeling of histone methylation and acetylation.

Trained immunity (innate immune memory) [68, 100] is a newly characterized metabolic and epigenetic remodeling of innate immune cells, which includes upregulation of glycolysis pathway enzymes, increased acetyl-CoA generation, upregulation of mevalonate pathway enzymes, and remodeling of histone methylation and acetylation. By analyzing microarray data of LIUS-treated human lymphoma cells, mouse preosteoblasts, and rat BM cells, we examined a novel hypothesis that LIUS induces its therapeutic effects in cells by modulating the expression of our newly reported [46] innate immune memory (trained immunity) pathway enzymes. As depicted in Figure 7(a), our results showed that LIUS downregulates 12 out of 102 trained immunity genes (11.8%), including 11 genes in BM cells and one gene in lymphoma cells. In addition, LIUS induces 11 out of 102 trained immunity genes (10.8%) including six genes in lymphoma cells, one gene in preosteoblasts, and four genes in BM cells. Moreover, in BM cells, the four trained immunity genes that LIUS upregulated and the 11 trained immunity genes that LIUS downregulated include eight glycolysis enzymes, one acetyl-CoA enzyme, and two mevalonate pathway enzymes, suggesting that 2.75-fold more downregulation than upregulation of trained immunity genes contributes significantly to LIUS-suppressed innate immunity and inflammation in BM cells. In contrast, in lymphoma cells, LIUS's upregulation of six trained immunity enzymes, including five glycolysis enzymes and one acetyl-CoA generation enzyme, and LIUS's downregulation of one glycolysis enzyme in lymphoma cells (sixfold more upregulation than downregulation) contribute significantly to LIUS-induced innate immunity enhancement in lymphoma cells.

These results suggest that LIUS enhancement of antitumor immune responses against lymphoma cells, at least partially, is associated with upregulation of trained immunity pathways, which is similar to that recently proposed by others [101]; LIUS also inhibits inflammation in noncancer cells by suppressing trained immunity pathways as we recently reported for human aortic endothelial cells activated by proatherogenic lipid lysophosphatidylcholine (Figure 7(b)) [27, 46].

### 3.7. LIUS Upregulation of IGs in Lymphoma Cells Uses Reverse Signaling of B7-H4, and LIUS Downregulation of IGs in Preosteoblast Cells and BM Counteracts the Reverse Signaling of B7-H4 and BTNL2

**Figure 8:**
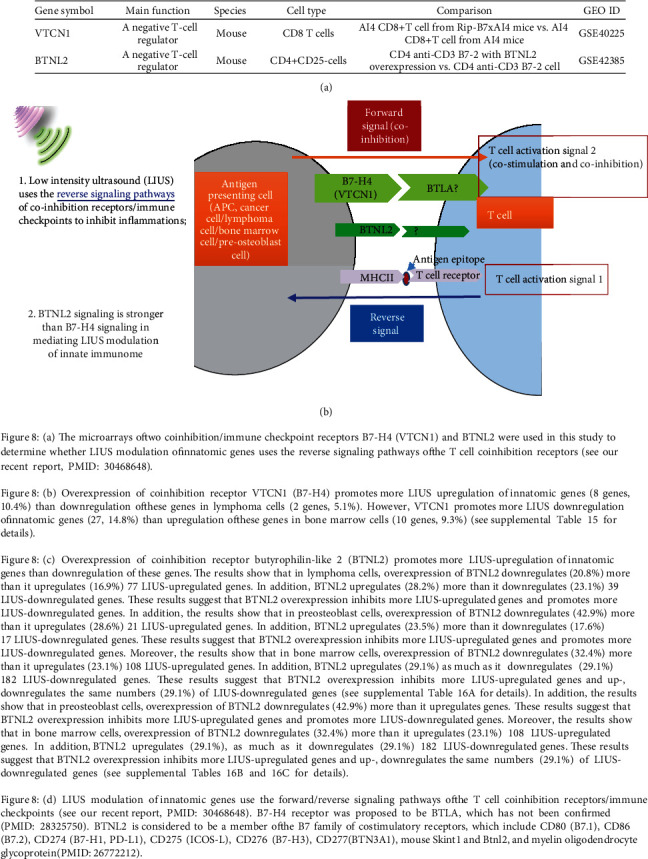


Our recent report determined the expression of 28 cosignaling receptors in 32 human tissues in physiological/pathological conditions and found that forward signaling (for modulation of T cell activation) and reverse signaling (for modulation of antigen-presenting cells) of 50% coinhibition receptors (CI/ICRs) are upregulated in endothelial cells during inflammation [40]. We hypothesized that LIUS regulates the innatome potentially via the reverse signaling (antigen-presenting cell aspect) of CI/ICRs. The microarrays of two CI/ICRs' (B7-H4 (VTCN1) and BTNL2) overexpression were used in this study to determine whether LIUS modulation of IGs uses the reverse signaling pathways of the CI/ICRs [40]. As displayed in Figure 8, the results showed that in lymphoma cells, overexpression of B7-H4 upregulated 10.4% (as opposed to downregulating 1.3%) of 77 LIUS-upregulated IGs, suggesting that LIUS upregulates the innatome potentially via the reverse signaling of B7-H4.

In addition, B7-H4 overexpression promoted 5.1% as well as decreased another 5.1% of 39 LIUS-downregulated IGs. Moreover, in preosteoblast cells, B7-H4 overexpression inhibited 4.8% of 21 LIUS-upregulated IGs. Also, B7-H4 overexpression promoted 11.8% of 17 LIUS-downregulated IGs. These results suggest that LIUS partially counteracts B7-H4 reverse signaling in downregulating IGs in preosteoblast cells.

Furthermore, in BM cells, B7-H4 overexpression promoted 9.3% (as opposed to downregulating 0.9%) of 108 LIUS-upregulated IGs. Finally, B7-H4 overexpression increased 14.8% (as opposed to downregulating 1.6%) of 182 LIUS-downregulated IGs. These results suggest that overexpression of CI/ICR B7-H4 promotes more LIUS-upregulated IGs in lymphoma cells and increases more LIUS-downregulated IGs in BM cells, supporting the conclusion that LIUS partially counteracts B7-H4 reverse signaling in downregulating IGs in BM cells.

As presented in Figure 8(c), the results showed that, in lymphoma cells, overexpression of the second CI/ICR butyrophilin-like 2 (BTNL2) downregulated 20.8% (as opposed to upregulating 16.9%) of LIUS-upregulated 77 genes. In addition, BTNL2 overexpression increased 28.2% (as opposed to downregulating 23.1%) of 39 LIUS-downregulated genes. These results suggest that BTNL2 overexpression inhibits more LIUS upregulated genes and promotes more LIUS-downregulated genes. In addition, the results showed that, in preosteoblast cells, overexpression of BTNL2 downregulates 42.9% (as opposed to upregulating 28.6%) of 21 LIUS-upregulated genes. In addition, BTNL2 increased 23.5% (as opposed to downregulating 17.6%) of 17 LIUS-downregulated genes. These results suggest that BTNL2 overexpression inhibits more LIUS-upregulated genes and promotes more LIUS-downregulated genes. Moreover, the results showed that, in BM cells, overexpression of BTNL2 downregulates 32.4% (as opposed to upregulating 23.1%) of 108 LIUS-upregulated genes. In addition, BTNL2 increased 29.1% as well as decreased another 29.1% of 182 LIUS-downregulated genes. These results suggest that LIUS partially counteracts BTNL2 reverse signaling in upregulating IGs in BM cells.

These results suggest that CI/ICR BTNL2 overexpression inhibits more LIUS-upregulated genes and upregulates and downregulates the same numbers (29.1%) of LIUS-downregulated genes; LIUS modulation of IGs uses the reverse signaling pathways of the CI/ICR; and LIUS may predominantly act via the reverse signaling of CI/ICR compared with previously discussed mechanisms (cytokines, static or oscillatory shear stress, and heat-generated mechanisms).

### 3.8. LIUS Upregulation of IGs Uses Reactive Oxygen Species (ROS) Pathways Significantly

**Figure 9:**
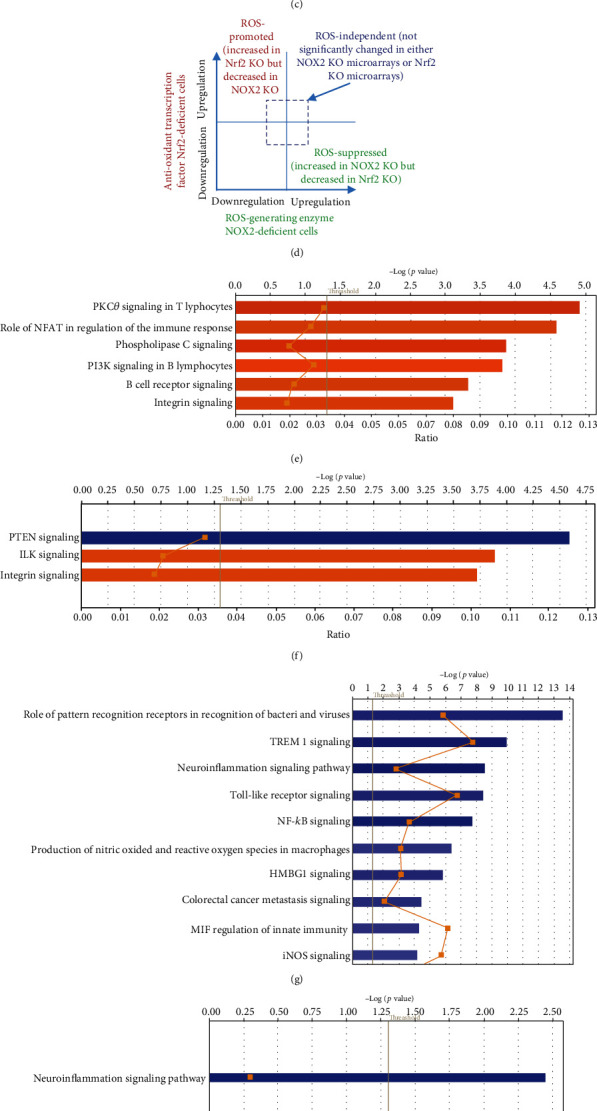
(a) LIUS modulates oxidative stress-related gene expressions (reactive oxygen species, ROS, and regulatome). Three out of a total of 84 (3.70%) oxidative stress genes (ROS regulatome) were upregulated in lymphoma cells (L) and bone marrow cells (B). Gpx3 was shared in L and B. Two out of a total of 84 genes (2.47%) were downregulated in bone marrow cells (B). No oxidative stress gene expressions were changed in the preosteoblast microarray dataset. (b) LIUS modulates the ROS regulatome in a cell-specific manner. (c) The Ingenuity Pathway Analyses (IPA) of ROS dependence to LIUS-modulated innatomic genes in three types of cells were summarized into four groups: (1) the ROS-promoted group, (2) the ROS-suppressed group, (3) the ROS-dependent/suppressed pathway-uncertain group, and (4) the ROS-independent group. These results demonstrated that LIUS-modulated innatomic genes were significantly mediated by ROS-promoted or ROS-suppressed pathways in bone marrow cells, which were higher than those of LIUS-modulated innatomic genes in lymphoma cells. In addition, the IPA-identified significant pathway numbers in bone marrow cells that were significantly higher than those of lymphoma cells, suggesting that ROS-modulated genes in lymphoma cells are much more diversified in the pathways than those of bone marrow cells (see supplemental Tables 17A-17F for details). (d) LIUS-modulated innatomic genes were classified into four groups, namely, (1) the ROS-promoted group, (2) the ROS-suppressed group, (3) the uncertain (not shown) group, and (4) the ROS-independent group, based on their expression changes in ROS-generating enzyme NOX2 KO microarrays and antioxidant transcription factor Nrf2 KO microarrays. (e) Six ROS-promoted pathways were involved in LIUS-upregulated genes in bone marrow cells. (f) Three ROS-suppressed pathways were involved in LIUS-upregulated genes in bone marrow cells. (g) The top 10 of a total of 31 ROS-promoted pathways were involved in LIUS-downregulated genes in bone marrow cells. (h) Only one ROS-suppressed pathway was involved in LIUS-downregulated genes in bone marrow cells. (I) Only one ROS-suppressed pathway was involved in LIUS-upregulated genes in lymphoma cells. (j) LIUS modulates innatome via reactive oxygen species (ROS) pathways mediated by pro-ROS generation enzyme nicotinamide adenine dinucleotide phosphate (NADPH) oxidase 2 (NOX2) (PMIDs: 21629295; 28916473) and antioxidant transcription factor nuclear erythroid-2 like factor (Nrf2) (PMID: 30610225).

It has been well documented that ROS plays a key role in regulating pathophysiological signaling in endothelial cell activation [102], cardiovascular diseases [103], and ultrasound therapy [104]. We also reported that mitochondrial ROS plays a significant role in EC activation [51, 105]. In addition, our new data in Figure 1(b) shows that LIUS modulated the antioxidant nuclear factor erythroid 2-related factor 2 (Nrf2) pathway. Moreover, to find evidence that ROS pathway genes are modulated by LIUS, 84 oxidative and antioxidative genes [106] were examined. As shown in Figures 9(a) and 9(b), LIUS upregulated two (thioredoxin reductase 1 (Txnrd1) and glutathione peroxidase 3 (Gpx3)) and downregulated two oxidative/antioxidative genes (apolipoprotein E (Apoe) and inducible NO synthase (Nos2)) in BM cells, respectively, and LIUS upregulated two oxidative/antioxidative genes such as Gpx3 and Nos2 in lymphoma cells, suggesting that LIUS modulated the ROS regulatome. However, an important question remains whether ROS signaling and antioxidant signaling mediate LIUS modulation of IGs. Thus, we examined a novel hypothesis that ROS signaling and antioxidant signaling mediate LIUS modulation of IGs. By mining the microarray datasets in the NIH-NCBI GEO DataSet database (https://www.ncbi.nlm.nih.gov/gds/), we found several microarray datasets with the deficiency or inhibition of nicotinamide adenine dinucleotide phosphate oxidase 2 (NOX2) [103] and the deficiency of antioxidant transcription factor nuclear factor erythroid 2-related factor 2 (Nrf2) [103]. We classified the LIUS-upregulated and downregulated IGs into four groups: first, ROS-promoted genes are those whose IG expressions are decreased in NOX2-deficient cells and increased in Nrf2-deficient cells; second, ROS-suppressed genes are those whose IGs increased in NOX2-deficient cells and decreased in Nrf2-deficient cells; third, ROS pathway-uncertain genes are those whose IGs were either promoted by ROS pathways in one group of microarray datasets or suppressed by ROS pathways in other groups of microarray datasets; and fourth, ROS-independent genes are those whose IGs were not significantly modulated in NOX2-deficient and Nrf2-deficient cells (Figure 9(d)).

As shown in Figure 9(c), in BM cells, 108 LIUS-upregulated IGs were classified into four groups with ROS-promoted (37%), ROS-suppressed (24%), uncertain (3.7%), and ROS-independent (35.2%) groups, suggesting that ROS-related LIUS upregulation of IGs is 64.8%. We also found that ROS-related LIUS downregulation of IGs in BM cells are 73.6%. Moreover, we found that ROS-related LIUS upregulation of IGs in lymphoma cells and preosteoblast cells are 65% and 62%, respectively, and that ROS-related LIUS downregulation of IGs in lymphoma cells and preosteoblast cells are 64.1% and 58.8%, respectively.

IPA showed that six promoting ROS-promoted pathways (orange color) were involved in LIUS-upregulated IGs in BM cells including protein kinase C-zeta (PKCzeta) signaling in T cells, the role of nuclear factor of activated T cells (NFAT) in regulation of immune response, phospholipase C signaling, phosphoinositide 3-kinase (PI3K) signaling in B cells, B cell receptor signaling, and integrin signaling (Figure 9(e)). In addition, as shown in Figure 9(f), three ROS-suppressed pathways (the one in blue color indicating suppressing) were involved in LIUS-upregulated IGs in BM cells including phosphatase and tensin homolog deleted on chromosome 10 (PTEN) signaling, integrin-linked kinase (ILK) signaling, and integrin signaling. Moreover, the top ten pathways out of the total of 31 ROS-promoted pathways were involved in LIUS-downregulated IGs in BM cells including the role of pattern recognition receptors, triggering receptor expressed on myeloid cells 1 (TREM1) signaling, neuroinflammation, Toll-like receptor signaling, NF-*κ*B signaling, production of nitric oxide (NO) and reactive oxygen species, high-mobility group protein 1 (HMGB1) signaling, colorectal cancer metastasis, macrophage migration inhibitory factor (MIF) regulation of innate immunity, and inducible NO synthase (INOS) signaling. Furthermore, only one ROS-suppressed pathway was involved in LIUS-downregulated IGs in BM cells, namely, neuroinflammation (Figure 9(h)); also, only one ROS-suppressed pathway was involved in LIUS-upregulated IGs in lymphoma cells (Figure 9(i)).

Taken together, these results have demonstrated for the first time that ROS pathways are a dominant mechanism in LIUS regulating innatome. We acknowledge that future studies using LIUS-treated noncancer and cancer cells will be warranted to examine this issue carefully and further determine whether any ROS pathways and antioxidant pathways [107] could modulate the activity of the innatome reported here. Of note, the IPA-identified signaling pathways for our new classification of LIUS-upregulated IGs and LIUS-downregulated IGs into ROS-promoted, ROS-suppressed, ROS-uncertain, and ROS-independent groups could not be comparable to that shown in Figures 1, 2, and 3, since reorganization of IGs according to the new classification made some pathways less significant in the IPA.

### 3.9. LIUS May Modulate Chromatin Long-Range Interactions to Regulate IG Expression in Cancer Cells and Noncancer Cells

The results from this study, our previous study [2], and others' reports indicate that LIUS regulates gene expression presumably at transcription levels. Our recent publication further reports that histone modification enzymes are significantly modulated in response to disease risk factor stimulations [108]; that IL-35 suppresses endothelial cell activation by inhibiting mitochondrial reactive oxygen species-mediated site-specific acetylation of histone 3 lysine 14 [51]; and that DNA damage factors and DNA repair factors serve as an integrated sensor and cell fate-determining machinery for all the intracellular stresses and dangers [109]. Our reports suggest that various nuclear programs control gene expression responses to endogenous and exogenous DAMPs and other stimuli, including LIUS [64].

We hypothesized that newly characterized chromatin long-range interactions (CLRI) differentially regulate the gene promoters to differentiate LIUS-modulated gene expression in cancer cells versus noncancer cells [42, 46]. To test this hypothesis with respect to LIUS's effects in modulating chromatin remodeling, we examined the expression changes of chromatin insulator-binding factors, such as CTCF and RAD21, and other promoter-binding factors and non-promoter-binding factors in LIUS-treated cancer cells and noncancer cells [110]. As shown in Table 10 of our recent paper [64], LIUS did not change the expression of two insulator-binding factors and 16 promoter-binding factors but changed the expression of one of six non-promoter-binding factors in cancer cells. In addition, LIUS changed the expression of two out of 16 promoter-binding factors in noncancer cells.

**Figure 10:**
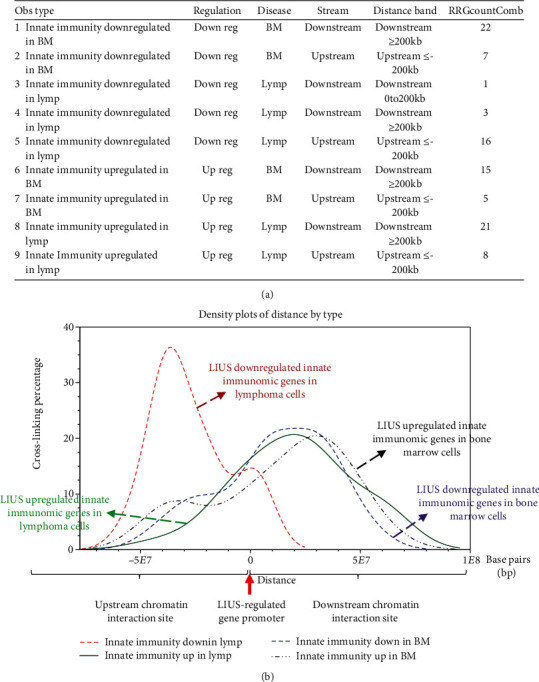
(a) Innate immunome chromatin looping makes long-range interactions that can be regulated by low-intensity ultrasound (LIUS). (a) Chromatin is a whole structure of complex DNA and proteins; it forms the chromosomes of eukaryotic organisms and is packaged inside the nucleus. Nucleosome is a basic unit of chromatin, consisting of a length of DNA coiled around a core of histones. (B) Chromatin looping makes gene promoter and distal regulatory elements come in close proximity and possibly interact with each other, which can be regulated by LIUS. (c) Long-range interactions allow communication between promoters and different distant regulatory elements (for a better understanding, please refer to Figure 4 of our recent paper published on Frontiers in Oncology 2019 at https://www.frontiersin.org/articles/10.3389/fonc.2019.00600/full). (b) LIUS modulates chromatin long-range interactions to regulate innatomic gene expressions in lymphoma cells (cancer cells) and bone marrow cells (the numbers of LIUS-regulated innatomic genes in preosteoblast cells were low so that chromatin long-range interaction data were too low to be analyzed). These results show that (i) the chromosome interaction zones are mostly located downstream of LIUS-upregulated innatomic genes in lymphoma cells, but the chromosome interaction zones are located in similar numbers both upstream and downstream of LIUS-upregulated genes in noncarcinoma cells, and (ii) the long-range interaction zones of LIUS-upregulated genes in lymphoma cells are located in a more concentrated manner both upstream and downstream (between 102 base pairs (bp) and 108 bp) than those of noncancer cells.

We further hypothesized that the differential gene expression seen between LIUS-treated cancer cells and noncancer cells was due to differences in the CLRIs of the modulated genes. Therefore, to analyze this, we obtained CLRI data for all the significantly modulated IGs from the 4DGenome database. This is a well-accepted database, which contains information on a huge collection of 4,433,071 experimentally derived CLRIs [82]. We then calculated the distances between 98 interacting sites (Figure 10(b)) with respect to LIUS-modulated gene promoters. If the LIUS-modulated gene promoter was located downstream of its long-range interaction partner, we designated the CLRI as negative. If the CLRI site is located downstream of the target gene promoter, we designated the interaction as positive. The two-sample Kolmogorov-Smirnov test of the CLRI distances between gene promoters corresponding to LIUS-downregulated and upregulated genes indicated some significant differences between the two distance distributions (*p* < 0.001) (Figure 10(b)).

Our data indicated that the majority of the CLRI sites for IGs that were downregulated by LIUS treatment were concentrated upstream between −5 × 10^7^ to -10^4^ base pairs (bp) in lymphoma cells (Figures 10(b)–10(b), the red dash line). In contrast, the majority of CLRI sites of the genes that were upregulated by LIUS were located downstream in lymphoma cells (Figures 10(b)–10(b), the green solid line). However, in noncancer BM cells, most of the CLRI sites of IGs modulated by LIUS were located downstream of the target genes. In comparison, most of the CLRI sites of IGs upregulated by LIUS were located more downstream of the target genes (the black dash line, Figures 10(b)–10(b)) than those of most of the CLRI sites of IGs downregulated by LIUS (the purple dash line, Figures 10(b)–10(b)) in the BM cells. In addition, in comparison, the CLRI sites of IGs upregulated by LIUS in BM cells (the black dash line, Figures 10(b)–10(b)) were located more upstream than the CLRI sites of IGs upregulated by LIUS in lymphoma cells (Figures 10(b)–10(b), the green solid line).

Future experiments will be needed to verify these interesting associations between CLRI sites and the genes that were modulated by LIUS treatment in both cancer and noncancer cells. Since the 4DGenome database contains the experimental data derived from human nonaortic endothelial cells [82], future work will be needed using circular chromosome conformation capture sequencing (4C-seq) to examine LIUS-treated cancer cells and noncancer cells to map the specific upstream interaction sites for modulation of cell death regulator expression in cancer cells and noncancer cells. Taken together, our results have demonstrated for the first time that LIUS induces a differential gene expression pattern in the innatome in lymphoma cells and noncancer BM cells, and that these genes have unique CLRI sites. Therefore, our results may suggest that optimal CLRI sites may serve as new therapeutic targets in the future to enhance LIUS-mediated cancer cell suppression and LIUS's anti-inflammatory functions in noncancer cells.

## 4. Discussion

Therapeutic applications of ultrasound, in addition to its use in diagnosis, are widely accepted to be beneficial. These benefits include the suppressive effect of LIUS on cancers via inducing cell death pathways [64]. As we pointed out previously [2, 64], the anti-inflammatory effects are responsible for inducing the clinical benefits mediated by LIUS in noncancer cells [111–113]. However, the molecular mechanisms underlying the anticancer cell functions and anti-inflammatory effects of LIUS remain poorly defined. Identification of novel molecular mechanisms underlying the anticancer cell functions and anti-inflammatory properties of LIUS in noncancer cells would significantly provide novel insights into this important issue, thus allowing for the improvement of LIUS-based therapeutics.

To fill in this crucial knowledge gap, in this study, we used cutting-edge molecular database mining approaches that we pioneered in 2004 [2, 34, 52, 64, 108, 114], which are different from the traditional literature review as we reported the comparisons between data mining papers and reviews in detail [115]. Our data analyses have made, for the first time, the following significant findings: (1) LIUS upregulates proinflammatory IGs and downregulates cancer metastasis genes in cancer cells. (2) LIUS has differential effects in suppression of danger signal sensing and of inflammation initiation in bone marrow cells (BM), enhancing IG expression for adaptive immune responses in BM. (3) LIUS-modulated IGs are regulated partially by inflammatory cytokines. (4) LIUS upregulates trained immunity enzymes in lymphoma cells but downregulates trained immunity enzymes in BM. (5) LIUS differentially upregulates more IGs that are encoded for proteins localized in the cytoplasm, extracellular space, and others, but it downregulates more IGs that encode proteins localized in the nucleus and plasma membrane; also, LIUS downregulates more phosphatases than the other 13 functional subgroups. (6) LIUS-modulated IGs are regulated by static or oscillatory shear stress and by using a more heat-generating mechanism in cancer cells than in noncancer cells. (7) Coinhibition/immune checkpoint receptor (CI/ICR) B7-H4 overexpression promotes LIUS-upregulated IGs in lymphoma cells and LIUS-downregulated IGs in BM; and CI/ICR BTNL2 overexpression inhibits more LIUS-upregulated IGs. (8) LIUS may modulate chromatin long-range interactions to regulate IG expression in cancer cells and noncancer cells.

It is not clear how LIUS exposure may transmit signals to the nucleus to modulate the IG expression in both cancer and noncancer cells. Previously, it was shown that LIUS can overstretch the cell membrane and cause reparable submicron pore formation [116]. This phenomenon is called sonoporation. Such effects may lead to disruption of the cytoskeleton in tandem because this network of subcellular filaments is physically interconnected with the plasma membrane [117]. Therefore, sonoporation associated with LIUS may be responsible for inducing important biological effects in cells. In addition, ultrasound at low diagnostic power can cause stable oscillations of the microbubbles, resulting in a transient increase in membrane permeability for Ca^2+^ [118, 119]. We previously reported that LIUS may make use of natural membrane vesicles as small as exosomes that are derived from immunosuppressor cells to fulfill its anti-inflammatory effects by upregulating the expression of extracellular vesicle/exosome biogenesis mediators and docking mediators [2]. In another recent paper, we reported that cancer cells and noncancer cells may use distinct signaling mechanisms to activate downstream targets when exposed to LIUS. We found that LIUS can activate more of the antioxidant effects in noncancer cells compared to cancer cells. Such changes in redox status of the cellular environment may lead to activation of sensors that may produce distinct gene expression patterns in noncancer cells relative to cancer cells exposed to LIUS. Treatment of localized tumors by focused ultrasound (FUS) is a minimally invasive therapy that uses a range of input energy for in situ tumor ablation through the generation of thermal and cavitation effects. A variant of FUS that delivers a reduced level of energy at the focal point and generates mild mechanical and thermal stress in target cells has the ability to increase immunogenic presentation of tumor antigens, which results in reversal of tumor-induced T cell tolerance [120]. Patients with trauma, invasive operations, anticancer treatment, organ transplantation, and probably ultrasound therapy produce a host of danger signals (e.g., nucleic acids, histone, high-mobility group box 1 protein, and S100) and high levels of proinflammatory and prothrombotic mediators, such as DAMPs and extracellular vesicles [121]. Therefore, LIUS produces differential biological responses in different cellular contexts by activating distinct transcription factors, downregulating phosphatases (reported in this paper), and modulating CLRI sites, thus activating IG expression patterns in cancer cells and noncancer cells.

Low-intensity pulsed ultrasound (LIPUS) is a form of ultrasound that delivers ultrasonic energy at a much lower intensity (<3 W/cm^2^) than traditional ultrasound energy and delivers output in the mode of a pulse wave, and it is typically used for therapeutic purpose in rehabilitation medicine and fracture healing [122]. For example, LIPUS may become an effective clinical procedure for the treatment of urological diseases, such as chronic prostatitis/chronic pelvic pain syndrome, erectile dysfunction, and stress urinary incontinence in the field of urology [96]. However, postoperative use of LIPUS after tibial fracture fixation does not accelerate radiographic healing and fails to improve functional recovery [123]. Taken together, the perspective, limitations, and drawbacks of ultrasound therapies also suggest the significance of molecular mechanistic findings presented here in order to improve our understanding on anti-inflammation therapies [43, 84, 97, 124].

**Figure 11:**
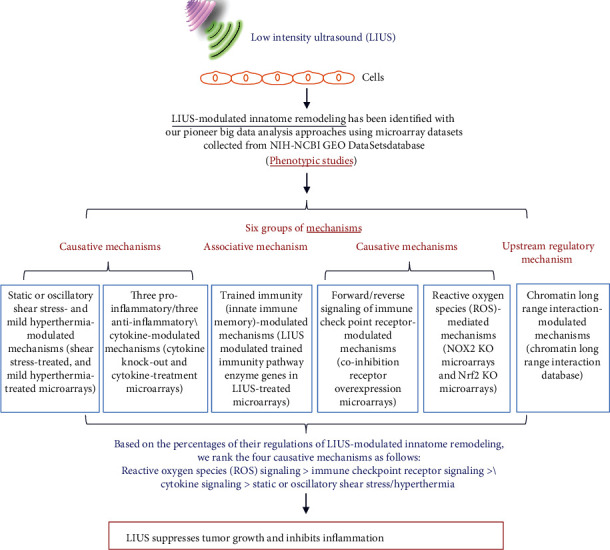
A new working model on LIUS-mediated cancer suppression and anti-inflammatory mechanisms via modulating innatome.

Based on our findings, we propose a new working model on LIUS-mediated cancer-suppressing and anti-inflammatory mechanisms as shown in Figure 11. Our new model integrates the following findings: First, LIUS induces IIG expression potentially via the pathways of ROS, immune checkpoint, trained immunity/tolerance, cytokines, and static or oscillatory shear stress/heat generation. Among four causative effects, based on the percentages of their regulations of LIUS-modulated innate immunome remodeling, we rank these four causative mechanisms as ROS > immune checkpoint receptor signaling > cytokine signaling > static or oscillatory shear stress/hyperthermia signaling (Figure 11). In our recent analytic review [31], based on different features and functions, we classified eleven types of ROS into seven functional groups: metabolic stress-sensing, chemical connecting, organelle communication, stress branch-out, inflammasome-activating, dual function, and triple function ROS. Among the ROS-generating systems, mitochondria consume the most amount of oxygen, and nine types of ROS are generated; thus, mitochondrial ROS systems serve as the central hub for connecting ROS with inflammasome activation, trained immunity, and immunometabolic pathways. In addition, other investigators found that release of mitochondrial DNA into the cytoplasm and out into the extracellular milieu activates a plethora of different pattern recognition receptors and innate immune responses, including cyclic GMP-AMP synthase- (cGAS-) stimulator of interferon genes (STING), Toll-like receptor 9, and inflammasome formation leading to, among others, robust type I interferon responses [125]. Second, most excitingly, LIUS induction of IIG expression is associated with LIUS induction of trained immunity enzyme expressions in lymphoma cells. The induction of trained immunity (innate immune memory) by LIUS in lymphoma cells enhances subsequent LIUS-promoted antitumor/lymphoma innate and adaptive immune responses. On the other hand, LIUS inhibition of IIG expression is associated with LIUS inhibition of trained immunity enzyme expressions in BM cells. The inhibition of trained immunity by LIUS in BM cells facilitate the establishment of trained immune tolerance, which contributes significantly to subsequently improved beneficial responses of inflammatory tissues/cells to LIUS therapeutics. Third, LIUS specifically downregulates phosphatases in both cancer and noncancer cells, which suggests that downregulations of phosphatases can serve as a clinical beneficial marker for LIUS therapies. Our results are also well correlated with previous reports showing that proinflammatory protein phosphatase 2A (PP2A) can be targeted for anticancer and anti-inflammatory drugs [91] and that proinflammatory protein phosphatase 6 can also be targeted [92]. Fourth, LIUS may modulate chromatin long-range interactions to differentially regulate the IIG expression in cancer cells and noncancer cells. Upstream chromatin long-range interaction sites (CLRISs) are more favorable than downstream CLRISs for LIUS modulation of IIG expression in cancer (lymphoma) cells; and in contrast, downstream CLRISs play more important roles than upstream CLRISs for LIUS downregulation of inflammatory pathways in noncancer BM cells.

One limitation of the current study is the unavailability of biological data obtained from LIUS-treated patient biopsies. We acknowledge that carefully designed in vitro and in vivo experimental models will be needed to further verify the LIUS-mediated cancer-suppressing and anti-inflammatory mechanisms we report here. These experimental models will enable consolidation of the efficacy of LIUS in various pathological conditions as well. However, the big datamining analyses that we pioneered in 2004 [114] have provided significant insight into LIUS-mediated modulation of the innatome via newly defined nuclear programs that induce innate immune responses in cancer cells and that downregulate more inflammatory pathways in noncancer cells [2, 64]. Once again [2, 64], our findings provide molecular readouts that can be used to determine optimal ultrasound intensity and duration, and will provide guidance for the development of future LIUS therapeutics for cancers, inflammation, tissue regeneration, and tissue repair.

## Figures and Tables

**Table:** (a) Six microarray datasets were analyzed in the LIUS-related phenotypic studies

Treatment	Disease	GEO ID	Organism	Cell	Method/parameter	Time	PMID
LIUS	Lymphoma	GSE10212	*Homo sapiens*	Lymphoma U937 cells	0.3 W/cm^2^, 1.0 MHz	1 min	18571840
	Noncancer	GSE45487	*Mus musculus*	MC3T3-E1 preosteoblast cells	0.03 W/cm^2^, 1.5 MHz	20 min	24252911
		GSE70662	*Rattus norvegicus*	Bone marrow cells from femora	N/A	15 min/day × 7 days	N/A

Mild hyperthermia	Lymphoma	GSE10043	*Homo sapiens*	Lymphoma U937 cells	41°C	30 min	18608577
	Noncancer	GSE39178	*Homo sapiens*	Fibroblast OUMS-36 cells	41°C	30 min	23311377

Oscillatory shear stress	Cancer	N/A	N/A	N/A	N/A	N/A	N/A
	Noncancer	GSE60152	*Homo sapiens*	Human lymphatic endothelial cells	1 dyn/cm^2^, 1/4 Hz	24 h	26389677

N/A: not applicable.

**Table:** (b) The expression levels of housekeeping genes in all the microarray datasets used for the LIUS-related phenotypic studies were not significantly altered

Housekeeping gene	UniGene ID		Fold change					
	Hs	Mm	GSE10212	GSE45487	GSE70662	GSE10043	GSE39178	GSE60152
CHMP2A	12107	295670	1.076	-1.025	-1.151	1.183	1.160	-1.002
PSMB4	89545	368	-1.015	-1.019	-1.044	1.032	1.087	-1.087
ACTB	520640	391967	1.013	-1.002	-1.094	1.063	1.128	-1.015
GAPDH	544577	304088	1.009	-1.005	1.240	1.139	1.284	-1.031

Abbreviations used: LIUS = low-intensity ultrasound; CHMP2A = charged multivesicular body protein 2A; PSMB4 = proteasome subunit beta 4; ACTB = actin beta; GAPDH = glyceraldehyde-3-phosphate dehydrogenase.

**Table 2:** 1376 innatomic genes^∗^ were studied in this report, all of which were collected from the InnateDB^#^ (PMID: 23180781).

Classification type	Subset	Number	Percentage
Subcellular location (5)	Cytoplasm	481	34.96%
	Extracellular space	151	10.97%
	Nucleus	405	29.43%
	Plasma membrane	86	6.25%
	Other	253	18.39%

Functional type (14)	Cytokine	53	3.85%
	Enzyme	232	16.86%
	G-protein-coupled receptor	22	1.60%
	Growth factor	21	1.53%
	Ion channel	8	0.58%
	Kinase	113	8.21%
	Ligand-dependent nuclear receptor	6	0.44%
	Other	459	33.36%
	Peptidases	54	3.92%
	Phosphatase	22	1.60%
	Transcription regulator	229	16.64%
	Translation regulator	10	0.73%
	Transmembrane receptor	88	6.40%
	Transporter	59	4.29%

Total		1376	100.00%

^#^InnateDB is being developed jointly by the Brinkman Laboratory (http://www.brinkman.mbb.sfu.ca) (Simon Fraser University, British Columbia, Canada), the Hancock Laboratory (http://cmdr.ubc.ca/bobh/) (University of British Columbia, Vancouver, British Columbia), and the Lynn EMBL Australia Group (http://www.emblaustralia.org/research-leadership/sa-node-lynn-group) (South Australian Health & Medical Research Institute and Flinders University, Adelaide, Australia). ^∗^The updated gene list at http://www.innatedb.com provides details of the innatomic genes which have been annotated by either InnateDB or Gene Ontology as having a role in the innatomic response and is updated weekly. Of note, when the list of 1467 genes was used in the Ingenuity Pathway Analysis (IPA), 1376 genes were recognized for classification.

**Table:** (a) The Ingenuity Pathway Analyses (IPA) showed that three out of five subcellular localization groups (cytoplasm, extracellular space, and others) of LIUS-upregulated innatomic genes in lymphoma cells, preosteoblast cells, and bone marrow cells are significantly changed. However, none of the 14 functional groups of LIUS-upregulated innatomic genes in these three cell types were changed

Classification type	Subset	Whole innate immunomic gene		Upregulated in lymphoma cells		Upregulated in preosteoblast cells		Upregulated in bone marrow cell	
		Number	Percentage	Number	Percentage	Number	Percentage	Number	Percentage
5 subcellular locations	Cytoplasm^∗^^,#,&^	481	34.96%	14	18.18%	2	9.52%	38	35.19%
	Extracellular space^∗^^,#^	151	10.97%	19	24.68%	9	42.86%	12	11.11%
	Nucleus	405	29.43%	21	27.27%	9	42.86%	34	31.48%
	Plasma membrane^∗^^,&^	86	6.25%	21	27.27%	1	4.76%	21	19.44%
	Other^∗^^,&^	253	18.39%	2	2.60%	1	4.76%	3	2.78%

14 functional types	Cytokine	53	3.85%	6	7.79%	0	0.00%	2	1.85%
	Enzyme	232	16.86%	6	7.79%	1	4.76%	18	16.67%
	G-protein-coupled receptor	22	1.60%	2	2.60%	0	0.00%	0	0.00%
	Growth factor	21	1.53%	2	2.60%	1	4.76%	1	0.93%
	Ion channel	8	0.58%	0	0.00%	0	0.00%	1	0.93%
	Kinase	113	8.21%	6	7.79%	1	4.76%	11	10.19%
	Ligand-dependent nuclear receptor^#^	6	0.44%	2	2.60%	1	4.76%	0	0.00%
	Other	459	33.36%	22	28.57%	7	33.33%	43	39.81%
	Peptidases	54	3.92%	4	5.19%	3	14.29%	3	2.78%
	Phosphatase	22	1.60%	1	1.30%	0	0.00%	1	0.93%
	Transcription regulator	229	16.64%	15	19.48%	6	28.57%	19	17.59%
	Translation regulator	10	0.73%	0	0.00%	0	0.00%	1	0.93%
	Transmembrane receptor	88	6.40%	7	9.09%	0	0.00%	3	2.78%
	Transporter	59	4.29%	4	5.19%	1	4.76%	5	4.63%

Total		1376		77		21		108	

^∗^ *P* value of comparison between whole innate immunity genes and upregulated gene in lymphoma cell dataset <0.05. ^#^*P* value of comparison between whole innate immunity genes and upregulated gene in preosteoblast cell dataset <0.05. ^&^*P* value of comparison between whole innate immunity genes and upregulated gene in bone marrow cell dataset <0.05.

**Table:** (b) The Ingenuity Pathway Analyses (IPA) show that two out of five subcellular localization groups (nucleus and plasma membrane) of LIUS-downregulated innatomic genes in lymphoma cells, preosteoblasts, and bone marrow cells are significantly changed. However, one of the 14 functional groups (phosphatase) of LIUS-downregulated innatomic genes in these three cell types are changed

Classification type	Subset	Whole innate immunity gene		Downregulated in lymphoma cells		Downregulated in preosteoblast cells		Downregulated in bone marrow cells	
		Number	Percentage	Number	Percentage	Number	Percentage	Number	Percentage
Subcellular location -5	Cytoplasm	481	34.96%	12	30.77%	6	35.29%	67	36.81%
	Extracellular space	151	10.97%	6	15.38%	3	17.65%	24	13.19%
	Nucleus^&^	405	29.43%	11	28.21%	4	23.53%	32	17.58%
	Plasma membrane^∗^^,&^	86	6.25%	10	25.64%	4	23.53%	49	26.92%
	Other^∗^^,&^	253	18.39%	0	0.00%	0	0.00%	10	5.49%

Functional type -14	Cytokine	53	3.85%	2	5.13%	2	11.76%	13	7.14%
	Enzyme	232	16.86%	5	12.82%	1	5.88%	26	14.29%
	G-protein-coupled receptor	22	1.60%	1	2.56%	0	0.00%	6	3.30%
	Growth factor	21	1.53%	1	2.56%	0	0.00%	2	1.10%
	Ion channel	8	0.58%	1	2.56%	0	0.00%	0	0.00%
	Kinase	113	8.21%	2	5.13%	0	0.00%	20	10.99%
	Ligand-dependent nuclear receptor	6	0.44%		0.00%	0	0.00%	1	0.55%
	Other	459	33.36%	12	30.77%	7	41.18%	50	27.47%
	Peptidases	54	3.92%		0.00%	0	0.00%	8	4.40%
	Phosphatase^∗^^,&^	22	1.60%	3	7.69%	0	0.00%	7	3.85%
	Transcription regulator	229	16.64%	6	15.38%	4	23.53%	20	10.99%
	Translation regulator	10	0.73%	1	2.56%	0	0.00%	0	0.00%
	Transmembrane receptor	88	6.40%	4	10.26%	2	11.76%	21	11.54%
	Transporter	59	4.29%	1	2.56%	1	5.88%	8	4.40%

Total		1376		39		17		182	

^∗^ *P* value of comparison between whole innate immunity genes and downregulated gene in lymphocyte dataset <0.05. ^#^*P* value of comparison between whole innate immunity genes and downregulated gene in protoplast dataset <0.05. ^&^*P* value of comparison between whole innate immunity genes and downregulated gene in bone marrow cell dataset <0.05.

## Data Availability

The microarray dataset that was utilized in the study was retrieved from the NIH GEO DataSet database (http://www.ncbi.nlm.nih.gov/gds/), and the number of the dataset is GSE10212, GSE45487, GSE70662, GSE10043, GSE39178, and GSE60152.

## Supplementary Materials

Supplementary Materials Supplemental Figure 1: the Ingenuity Pathway Analysis (IPA) profiles general innatomic genes, which can be categorized by diseases or functions via number of genes. Supplemental Table 1: low-intensity ultrasound (LIUS) upregulated 77 out of 1376 (5.6%) innatomic genes and downregulated 39 out of 1376 (2.8%) innatomic genes (IIGs) in human lymphoma U937 cells (GSE10212). Supplemental Table 2: low-intensity ultrasound (LIUS) upregulates 21 out of 1376 (1.5%) innatomic genes and downregulates 17 out of 1376 (1.2%) innatomic genes in mouse preosteoblast cells. Supplemental Table 3: LIUS-modulated innatomic genes in bone marrow cells (part 1). Supplemental Table 4: the static or oscillatory shear stress conditions upregulated eight LIUS-upregulated innatomic genes including 2 genes (out of 77) in lymphoma cells, one (out of 21) in preosteoblasts and five (out of 108) in bone marrow cells. Supplemental Table 5: the 82 heat shock proteins in the heat shock family are classified into 4 groups including (a) heat shock 90 kDa proteins [5], (b) DNAJ (HSP40) heat shock proteins [49], (c) small heat shock proteins [11], and (d) heat shock 70 kDa proteins [17]. Supplemental Table 6: low-intensity ultrasound (LIUS) upregulated heat shock protein expressions in lymphoma cells but downregulated heat shock protein expressions in noncancer cells. Supplemental Table 7: the mild hyperthermia treatment (41°C) upregulated 15 LIUS-upregulated innatomic genes in fibroblast OUMS-36 cells including 6 genes in lymphoma cells (L), 2 genes in preosteoblast cells, and 7 genes in bone marrow cells. Supplemental Table 8: the mild hyperthermia treatment (41°C) upregulated 45 LIUS-upregulated innatomic genes in human lymphoma U937 cells including 20 genes (out of 77, 26%) in lymphoma cells (L), 6 (out of 21, 28.6%) in preosteoblasts, and 19 (out of 108, 17.6%) in bone marrow cells. Supplemental Table 9: by analyzing the microarray data from cytokine gene knock-out (KO) cells or cytokine-treated cells, we found that LIUS-upregulated innatomic genes in human lymphoma cells can be modulated by a set of cytokines. Supplemental Table 10: by analyzing the microarray data from cytokine gene KO cells or cytokine-treated cells, we found that LIUS-downregulated innatomic genes in human lymphoma cells can be modulated by a set of cytokines. Supplemental Table 11: by analyzing the microarray data from cytokine gene knock-out (KO) cells or cytokine-treated cells, we found that LIUS-upregulated innatomic genes in mouse preosteoblast cells can be modulated slightly by a set of cytokines. Supplemental Table 12: by analyzing the microarray data from cytokine gene knock-out (KO) cells or cytokine-treated cells, we found that LIUS-downregulated innatomic genes in mouse preosteoblast cells can be modulated slightly by a set of cytokines. Supplemental Table 13: by analyzing the microarray data from cytokine gene knock-out (KO) cells or cytokine-treated cells, we found that LIUS-upregulated innatomic genes in rat bone marrow cells can be modulated by a set of cytokines. Supplemental Table 14: by analyzing the microarray data from cytokine gene knock-out (KO) cells or cytokine-treated cells, we found that LIUS-downregulated innatomic genes in rat bone marrow cells can be modulated by a set of cytokines. Supplemental Table 15A: the microarrays of two T cell coinhibition receptors B7-H4 (VTCN1) and BTNL2 were used in this study to determine whether LIUS modulation of innatomic genes uses the reverse signaling pathways of the T cell coinhibition receptors (see our recent report, PMID: 30468648). Supplemental Table 15B: overexpression of coinhibition receptor VTCN1 (B7-H4) promotes more LIUS-upregulated innatomic genes (8 genes, 10.4%) than downregulating these genes in lymphoma cells (2 genes, 5.1%). However, VTCN1 promotes more LIUS-downregulated innatomic genes (27, 14.8%) than upregulating these genes in bone marrow cells (10 genes, 9.3%). Supplemental Table 16A: overexpression of coinhibition receptor butyrophilin-like 2 (BTNL2) promotes more LIUS-upregulated innatomic genes than downregulating these genes. Supplemental Tables 16B and 16C: in addition, the results showed that in preosteoblast cells, overexpression of BTNL2 downregulates 42.9%, more than the upregulation (28.6%) of LIUS-upregulated 21 genes. In addition, BTNL2 increased 23.5%, more than the downregulation (17.6%) of 17 LIUS-downregulated genes. Supplemental Table 17A: LIUS-upregulated genes in bone marrow cells are classified into four groups, namely, the reactive oxygen species- (ROS-) dependent, ROS-suppressed, ROS-dependent/suppressed, and ROS-independent groups. Supplemental Table 17B: LIUS-downregulated genes in bone marrow cells are classified into four groups, namely, the reactive oxygen species- (ROS-) dependent, ROS-suppressed, ROS-dependent/suppressed, and ROS-independent groups. Supplemental Table 17C: LIUS-upregulated genes in lymphoma cells are classified into four groups, namely, the reactive oxygen species- (ROS-) dependent, ROS-suppressed, ROS-dependent/suppressed, and ROS-independent groups. Supplemental 17D: LIUS-downregulated genes in lymphoma cells are classified into four groups, namely, the reactive oxygen species- (ROS-) dependent, ROS-suppressed, ROS-dependent/suppressed, and ROS-independent groups. Supplemental Table 17E: LIUS-upregulated genes in preosteoblast cells are classified into four groups, namely, the reactive oxygen species- (ROS-) dependent, ROS-suppressed, ROS-dependent/suppressed, and ROS-independent groups. Supplemental Table 17F: LIUS-downregulated genes in preosteoblast cells are classified into four groups, namely, the reactive oxygen species- (ROS-) dependent, ROS-suppressed, ROS-dependent/suppressed, and ROS-independent groups.
