# Supplementary material for: Ultrasound May Suppress Tumor Growth, Inhibit Inflammation, and Establish Tolerogenesis by Remodeling Innatome via Pathways of ROS, Immune Checkpoints, Cytokines, and Trained Immunity/Tolerance
Source: J Immunol Res. 2021 Feb 9;2021:6664453. doi: 10.1155/2021/6664453 (PMC7889351; doi:10.1155/2021/6664453)
Supplement: Supplementary Materials — Supplemental Figure 1: the Ingenuity Pathway Analysis (IPA) profiles general innatomic genes, which can be categorized by diseases or functions via number of genes. Supplemental Table 1: low-intensity ultrasound (LIUS) upregulated 77 out of 1376 (5.6%) innatomic genes and downregulated 39 out of 1376 (2.8%) innatomic genes (IIGs) in human lymphoma U937 cells (GSE10212). Supplemental Table 2: low-intensity ultrasound (LIUS) upregulates 21 out of 1376 (1.5%) innatomic genes and downregulates 17 out of 1376 (1.2%) innatomic genes in mouse preosteoblast cells. Supplemental Table 3: LIUS-modulated innatomic genes in bone marrow cells (part 1). Supplemental Table 4: the static or oscillatory shear stress conditions upregulated eight LIUS-upregulated innatomic genes including 2 genes (out of 77) in lymphoma cells, one (out of 21) in preosteoblasts and five (out of 108) in bone marrow cells. Supplemental Table 5: the 82 heat shock proteins in the heat shock family are classified into 4 groups including (a) heat shock 90 kDa proteins [5], (b) DNAJ (HSP40) heat shock proteins [49], (c) small heat shock proteins [11], and (d) heat shock 70 kDa proteins [17]. Supplemental Table 6: low-intensity ultrasound (LIUS) upregulated heat shock protein expressions in lymphoma cells but downregulated heat shock protein expressions in noncancer cells. Supplemental Table 7: the mild hyperthermia treatment (41°C) upregulated 15 LIUS-upregulated innatomic genes in fibroblast OUMS-36 cells including 6 genes in lymphoma cells (L), 2 genes in preosteoblast cells, and 7 genes in bone marrow cells. Supplemental Table 8: the mild hyperthermia treatment (41°C) upregulated 45 LIUS-upregulated innatomic genes in human lymphoma U937 cells including 20 genes (out of 77, 26%) in lymphoma cells (L), 6 (out of 21, 28.6%) in preosteoblasts, and 19 (out of 108, 17.6%) in bone marrow cells. Supplemental Table 9: by analyzing the microarray data from cytokine gene knock-out (KO) cells or cytokine-treated cells [file 6664453.f1.docx]

**Supplemental Figure 1. The Ingenuity Pathway Analysis (IPA) profiles general innatomic genes, which can be categorized by diseases or functions via number of genes. The five groups with the smallest P value are highlighted in green boxes by IPA, which include: 1) quantity of leukocytes; 2) quantity of blood cells; 3) necrosis; 4) proliferation of blood cells; and 5) apoptosis.**

**
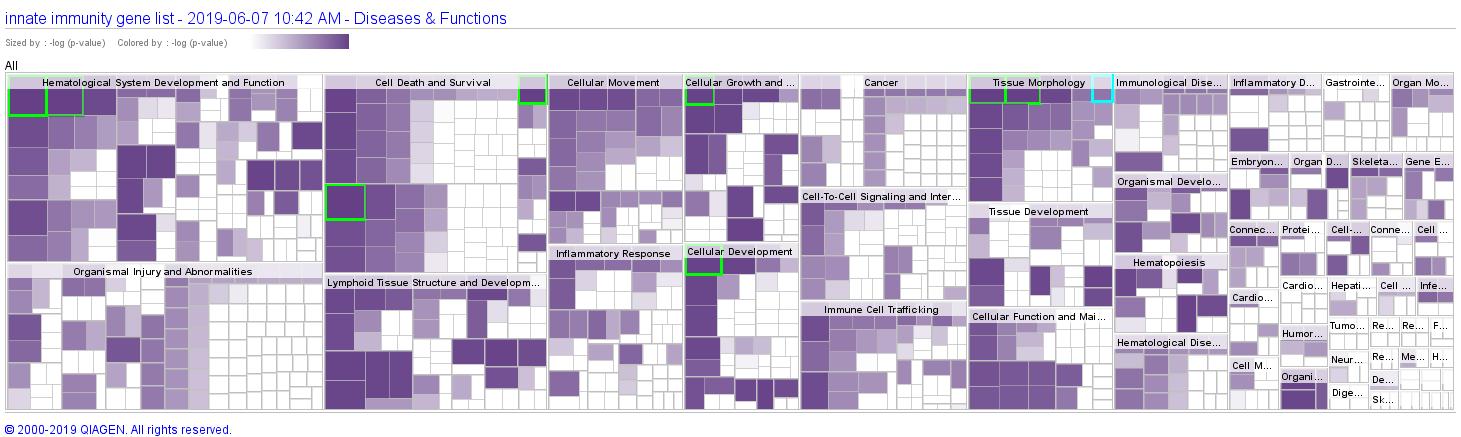
**

**Supplemental Table 1. Low intensity ultrasound (LIUS) upregulated 77 out of 1376 (5.6%) innatomic genes, and downregulated 39 out of 1376 genes (2.8%) innatomic genes (IIGs) in human lymphoma U937 cells (GSE10212),** suggesting that: 1) LIUS increases innatomic gene expressions more than decreasing them in cancer cells in human lymphoma cells; and 2) upregulation of innatomic genes in lymphoma cells serves as a novel immune mechanism underlying anti-tumor effects of LIUS.

| Up-regulated Genes (N=77, 5.60%) | | | Down-regulated Genes (N=39, 2.83%) | | |
| --- | --- | --- | --- | --- | --- |
| Gene | P value | Fold change | Gene | P value | Fold change |
| PCDH7 | 0.01 | 21.66 | CD47 | 0.00 | 0.06 |
| SERPINE1 | 0.00 | 20.21 | SCN11A | 0.01 | 0.11 |
| HMOX1 | 0.00 | 11.64 | ITGB1 | 0.00 | 0.13 |
| IL1RL1 | 0.00 | 10.23 | LRP2BP | 0.01 | 0.13 |
| IL10 | 0.00 | 9.56 | GJA1 | 0.00 | 0.15 |
| MERTK | 0.03 | 7.75 | AFF1 | 0.00 | 0.18 |
| DSG1 | 0.01 | 7.60 | TLR7 | 0.01 | 0.18 |
| EDNRB | 0.00 | 7.50 | BHMT | 0.04 | 0.18 |
| LAMC3 | 0.01 | 7.24 | LPAR1 | 0.00 | 0.23 |
| SLC2A1 | 0.01 | 6.38 | SST | 0.02 | 0.24 |
| GSTM5 | 0.00 | 5.82 | MAPK8IP1 | 0.03 | 0.26 |
| MFGE8 | 0.00 | 5.67 | PML | 0.00 | 0.26 |
| SLAMF7 | 0.00 | 5.64 | PTPN11 | 0.00 | 0.28 |
| MAFB | 0.00 | 5.59 | SFTPC | 0.05 | 0.32 |
| NR4A3 | 0.01 | 5.58 | PON1 | 0.01 | 0.35 |
| JUN | 0.02 | 5.00 | CCL19 | 0.04 | 0.35 |
| ELL2 | 0.00 | 4.98 | RGS3 | 0.01 | 0.36 |
| RGS1 | 0.00 | 4.58 | Akr1b10 | 0.04 | 0.37 |
| SQSTM1 | 0.00 | 4.58 | CLEC4E | 0.02 | 0.38 |
| NOS2 | 0.04 | 4.37 | MTSS1 | 0.00 | 0.39 |
| ITGB3 | 0.03 | 4.16 | EZH1 | 0.01 | 0.40 |
| CDK12 | 0.00 | 4.11 | CCNG2 | 0.03 | 0.48 |
| LHX1 | 0.03 | 3.97 | SRC | 0.03 | 0.50 |
| FABP7 | 0.02 | 3.94 | APBB2 | 0.04 | 0.53 |
| PHLDA1 | 0.00 | 3.62 | CSF3 | 0.02 | 0.54 |
| TRAF1 | 0.00 | 3.62 | TLR1 | 0.02 | 0.56 |
| CD40 | 0.00 | 3.52 | ABTB2 | 0.05 | 0.58 |
| SCARB1 | 0.03 | 3.47 | ID1 | 0.03 | 0.58 |
| XIST | 0.01 | 3.42 | ARHGDIA | 0.03 | 0.61 |
| ZFP36L1 | 0.00 | 3.32 | DUSP2 | 0.05 | 0.62 |
| GPX3 | 0.02 | 3.32 | CHAC1 | 0.04 | 0.62 |
| NAB2 | 0.03 | 3.25 | NFE2 | 0.04 | 0.63 |
| IL7R | 0.00 | 2.87 | EIF2B1 | 0.03 | 0.63 |
| SGK1 | 0.00 | 2.74 | BBX | 0.04 | 0.64 |
| NR4A1 | 0.04 | 2.71 | Tnik | 0.04 | 0.65 |
| C3 | 0.01 | 2.71 | RAB33A | 0.05 | 0.65 |
| FOSL2 | 0.01 | 2.51 | Pvr | 0.04 | 0.65 |
| APOB | 0.02 | 2.46 | HBEGF | 0.04 | 0.65 |
| PTX3 | 0.01 | 2.34 | ZFP36L2 | 0.04 | 0.65 |
| GDF15 | 0.01 | 2.32 |  |  |  |
| MAFK | 0.01 | 2.28 |  |  |  |
| Ccl8 | 0.04 | 2.24 |  |  |  |
| FTH1 | 0.01 | 2.15 |  |  |  |
| KLF6 | 0.02 | 2.08 |  |  |  |
| PKN2 | 0.04 | 2.04 |  |  |  |
| DUSP4 | 0.03 | 2.02 |  |  |  |
| ADM | 0.01 | 2.01 |  |  |  |
| Ccl2 | 0.01 | 1.96 |  |  |  |
| CCL2 | 0.01 | 1.96 |  |  |  |
| S100A10 | 0.01 | 1.96 |  |  |  |
| C18orf25 | 0.01 | 1.95 |  |  |  |
| BMP2 | 0.02 | 1.93 |  |  |  |
| IER3 | 0.02 | 1.93 |  |  |  |
| F3 | 0.03 | 1.92 |  |  |  |
| Rasal2 | 0.03 | 1.91 |  |  |  |
| Tsc22d3 | 0.01 | 1.91 |  |  |  |
| CD44 | 0.02 | 1.89 |  |  |  |
| MBD2 | 0.03 | 1.81 |  |  |  |
| IL2RB | 0.04 | 1.80 |  |  |  |
| CCR1 | 0.02 | 1.77 |  |  |  |
| Sp100 | 0.03 | 1.74 |  |  |  |
| PIM1 | 0.03 | 1.70 |  |  |  |
| EZR | 0.03 | 1.67 |  |  |  |
| RGS2 | 0.02 | 1.67 |  |  |  |
| SERPINF1 | 0.03 | 1.67 |  |  |  |
| ICAM1 | 0.04 | 1.66 |  |  |  |
| MMP14 | 0.05 | 1.66 |  |  |  |
| CSF1 | 0.03 | 1.64 |  |  |  |
| CAPN2 | 0.03 | 1.64 |  |  |  |
| MDM2 | 0.04 | 1.63 |  |  |  |
| SDC4 | 0.05 | 1.62 |  |  |  |
| ADCY2 | 0.05 | 1.58 |  |  |  |
| NDEL1 | 0.04 | 1.57 |  |  |  |
| CCL20 | 0.05 | 1.57 |  |  |  |
| CXCL10 | 0.05 | 1.56 |  |  |  |
| MAFF | 0.04 | 1.54 |  |  |  |
| FYN | 0.05 | 1.50 |  |  |  |

**Supplemental Table 2. Low intensity ultrasound (LIUS) upregulates 21 out of 1376 (1.5%) innatomic genes and downregulates 17 out of 1376 genes (1.2%) innatomic genes in mouse pre-osteoblast cells (GSE45487),** suggesting that LIUS increases innatomic gene expressions slightly more than decreasing them in mouse preosteoblast cells (Patients following organ transplantation and treated with inhibitors of the Calcineurin/NFATc1 pathway, such as cyclosporin A and FK506 often develop osteoporosis. PMID: 11210996; PMID: 28390147).

| up-regulated genes (N=21; 1.53%) | | | down-regulated genes (N=17; 1.24%) | | |
| --- | --- | --- | --- | --- | --- |
| Symbol | P value | FC | Symbol | P value | FC |
| MMP13 | 0.00 | 2.25 | IL1RN | 0.04 | 0.74 |
| APOD | 0.00 | 1.96 | KLF10 | 0.04 | 0.73 |
| THBS1 | 0.00 | 1.69 | CHST1 | 0.04 | 0.73 |
| MMP9 | 0.00 | 1.63 | HMMR | 0.04 | 0.73 |
| EGR3 | 0.01 | 1.62 | HOMER2 | 0.04 | 0.72 |
| NR4A1 | 0.00 | 1.60 | BLNK | 0.05 | 0.72 |
| VEGFC | 0.01 | 1.60 | LRP1 | 0.02 | 0.69 |
| SERPINE1 | 0.01 | 1.58 | Pmaip1 | 0.03 | 0.66 |
| LMCD1 | 0.00 | 1.56 | LTBP2 | 0.01 | 0.66 |
| PLAGL1 | 0.01 | 1.51 | Tmeff2 | 0.01 | 0.66 |
| Tpm1 | 0.04 | 1.49 | EDN1 | 0.01 | 0.64 |
| ADAMTS1 | 0.03 | 1.47 | CDH1 | 0.02 | 0.58 |
| SRF | 0.03 | 1.45 | FABP4 | 0.02 | 0.55 |
| MYC | 0.03 | 1.44 | ID2 | 0.00 | 0.53 |
| CH25H | 0.03 | 1.37 | ID1 | 0.00 | 0.52 |
| DBP | 0.04 | 1.37 | KRT14 | 0.00 | 0.51 |
| RGS2 | 0.03 | 1.36 | ID3 | 0.00 | 0.50 |
| CDK5R1 | 0.05 | 1.35 |  |  |  |
| RGS3 | 0.03 | 1.34 |  |  |  |
| IGFBP4 | 0.03 | 1.33 |  |  |  |
| TFPI | 0.04 | 1.31 |  |  |  |

**Supplemental Table 3. LIUS-modulated innatomic genes in bone marrow cells (part 1)**

| upregulated gene (N=108) | | | downregulated gene (N=182) | | |
| --- | --- | --- | --- | --- | --- |
| Symbol | p value | FC | Symbol | p value | FC |
| CD3E | 0.012854 | 7.311149 | DUSP4 | 0.000157 | 0.058801 |
| PCDHB7 | 0.01506 | 5.225763 | CDH1 | 9.61E-06 | 0.08111 |
| Pcdhb7 | 0.01506 | 5.225763 | NEIL3 | 0.001536 | 0.095 |
| THBS1 | 0.00013 | 3.728397 | IL7R | 0.014261 | 0.109669 |
| ZBTB10 | 0.013578 | 3.12797 | IL10RA | 0.004607 | 0.112023 |
| CHAC1 | 0.010925 | 2.939513 | SFTPC | 0.000613 | 0.113644 |
| TIMP1 | 0.000215 | 2.933617 | GCA | 0.026654 | 0.114572 |
| PLA2G5 | 0.001206 | 2.919021 | SLAMF7 | 0.008507 | 0.118605 |
| PHLDA1 | 0.0003 | 2.840528 | WFDC2 | 0.003376 | 0.119116 |
| SLC4A7 | 0.030625 | 2.715134 | DBNDD1 | 0.006014 | 0.122546 |
| DLX3 | 0.012531 | 2.710385 | MAP3K2 | 0.014327 | 0.138485 |
| GAS2 | 0.043496 | 2.648424 | Ccl9 | 0.031102 | 0.144808 |
| FYN | 0.002111 | 2.475748 | CCL22 | 0.01624 | 0.150383 |
| SLC38A2 | 0.001947 | 2.386853 | ADCY3 | 0.024976 | 0.164628 |
| THBS4 | 0.002097 | 2.378684 | GBP4 | 0.009377 | 0.170436 |
| GORAB | 0.019488 | 2.349391 | ARHGAP25 | 0.025173 | 0.175683 |
| BLNK | 0.000893 | 2.328978 | CKLF | 0.004757 | 0.177341 |
| CHD8 | 0.00583 | 2.309488 | MAPK9 | 0.025675 | 0.187188 |
| SKIL | 0.006279 | 2.287508 | IL1RL1 | 0.000848 | 0.191092 |
| METRNL | 0.001151 | 2.247783 | EBI3 | 6.94E-05 | 0.201859 |
| PCDH7 | 0.009736 | 2.221125 | NHLRC1 | 0.006263 | 0.203043 |
| POLR3F | 0.01844 | 2.178247 | C3AR1 | 0.005344 | 0.206907 |
| LMAN1 | 0.002058 | 2.173829 | WAS | 0.025226 | 0.215519 |
| DDR2 | 0.006251 | 2.170695 | RSAD2 | 0.01677 | 0.230983 |
| TJP1 | 0.009243 | 2.166494 | TROVE2 | 0.001182 | 0.235216 |
| RGS3 | 0.001851 | 2.147369 | CCR1 | 0.001719 | 0.23605 |
| FKBP1B | 0.021044 | 2.13183 | MAFB | 0.000237 | 0.236879 |
| BCAR1 | 0.002956 | 2.125448 | Clec4a3 | 0.00036 | 0.237703 |
| SOS1 | 0.028472 | 2.119617 | TLR2 | 0.000267 | 0.24587 |
| MAP3K14 | 0.044391 | 2.069977 | PLAU | 0.008363 | 0.258483 |
| POLR3D | 0.009127 | 2.054145 | NPR3 | 0.033631 | 0.283452 |
| INSIG2 | 0.004848 | 2.0514 | VCAM1 | 0.000407 | 0.284616 |
| SPAG9 | 0.002522 | 2.018899 | CCDC39 | 0.038809 | 0.287041 |
| SSR1 | 0.002508 | 2.009341 | TLR4 | 0.001171 | 0.301296 |
| ENC1 | 0.005066 | 2.007742 | HHEX | 0.004256 | 0.301606 |
| GPX3 | 0.014863 | 1.995721 | CNR2 | 0.000237 | 0.302978 |
| MMP13 | 0.00341 | 1.977803 | CH25H | 0.000755 | 0.307149 |
| PLK2 | 0.005396 | 1.956415 | MSC | 0.013437 | 0.30822 |
| SCARF2 | 0.012938 | 1.951678 | IL10 | 0.01018 | 0.312415 |
| DYRK2 | 0.006787 | 1.943471 | PIM1 | 0.04579 | 0.314325 |
| PLOD2 | 0.010294 | 1.906448 | MYT1 | 0.016903 | 0.336764 |
| PFKFB3 | 0.009477 | 1.905493 | FCER1G | 0.000553 | 0.342677 |
| CCNG2 | 0.03417 | 1.898198 | FOS | 0.00998 | 0.342853 |
| UBA1 | 0.025569 | 1.882256 | Ccl2 | 0.003971 | 0.345258 |
| LRP4 | 0.010553 | 1.876958 | CCL2 | 0.003971 | 0.345258 |
| IER5 | 0.048565 | 1.852981 | TLR7 | 0.001373 | 0.350342 |
| ACSL1 | 0.012305 | 1.839462 | ALCAM | 0.004115 | 0.354963 |
| BCL6 | 0.02255 | 1.838788 | LAMC3 | 0.043218 | 0.361228 |
| ELL2 | 0.015631 | 1.825623 | ARC | 0.010304 | 0.361882 |
| CSF1 | 0.008027 | 1.804834 | SYK | 0.000595 | 0.369134 |
| BHMT | 0.02243 | 1.801665 | ICAM2 | 0.01833 | 0.377207 |
| ISG20 | 0.012009 | 1.797386 | CDH5 | 0.019053 | 0.377403 |
| TBPL1 | 0.015201 | 1.773058 | NEURL3 | 0.004287 | 0.378463 |
| CTNNB1 | 0.00529 | 1.771689 | LMO2 | 0.003113 | 0.380079 |
| RAPH1 | 0.009902 | 1.767454 | FCGR1A | 0.000818 | 0.380287 |
| NFKBIA | 0.005897 | 1.765065 | CDKL1 | 0.00371 | 0.380298 |
| COPZ2 | 0.008211 | 1.755791 | CENPA | 0.001082 | 0.385163 |
| PPFIBP1 | 0.007005 | 1.750828 | CD83 | 0.001541 | 0.38746 |
| MDM2 | 0.01399 | 1.74683 | RRM2 | 0.037407 | 0.390779 |
| GMCL1 | 0.012728 | 1.732449 | TIRAP | 0.006239 | 0.395226 |
| PDGFRL | 0.016912 | 1.719118 | CRYM | 0.003177 | 0.396456 |
| HOMER2 | 0.015185 | 1.714427 | EGR1 | 0.017956 | 0.396883 |
| HDAC5 | 0.044345 | 1.705609 | ITGB2 | 0.002173 | 0.402829 |
| ABCA1 | 0.015011 | 1.69555 | TMEM176B | 0.002297 | 0.405689 |
| EHD1 | 0.025004 | 1.694048 | TGIF1 | 0.002836 | 0.406361 |
| GJA1 | 0.011412 | 1.692474 | SAT1 | 0.001846 | 0.408042 |
| PTPN4 | 0.035392 | 1.686779 | ELOVL3 | 0.0499 | 0.408809 |
| PER1 | 0.006279 | 1.685662 | AMPD3 | 0.001707 | 0.409387 |
| ID2 | 0.009859 | 1.684086 | NLRP3 | 0.002808 | 0.416239 |
| COQ10B | 0.020293 | 1.679924 | RCSD1 | 0.001482 | 0.416477 |
| SDC2 | 0.007988 | 1.675332 | Tnfsf9 | 0.008458 | 0.416927 |
| PARVA | 0.017219 | 1.6521 | IRF4 | 0.026417 | 0.418319 |
| GADD45B | 0.012454 | 1.649253 | HMOX1 | 0.001152 | 0.418906 |
| GLB1 | 0.009533 | 1.627358 | CD163 | 0.001823 | 0.421568 |
| GSPT1 | 0.031725 | 1.619256 | TNFSF13 | 0.001079 | 0.421738 |

Part 2 (Continued…)

| upregulated gene (N=108) | | | downregulated gene (N=182) | | |
| --- | --- | --- | --- | --- | --- |
| Symbol | p value | FC | Symbol | p value | FC |
| PPP1R10 | 0.026246 | 1.600522 | APOB | 0.029638 | 0.423421 |
| NEDD9 | 0.022701 | 1.598312 | ADAM9 | 0.002209 | 0.426078 |
| RNF19A | 0.020478 | 1.588982 | EDNRB | 0.014812 | 0.426324 |
| IRS1 | 0.013937 | 1.586814 | Ifitm1 | 0.000929 | 0.429182 |
| FUBP1 | 0.021174 | 1.585596 | SLC30A1 | 0.005528 | 0.43232 |
| OSTC | 0.022162 | 1.580427 | C3 | 0.010269 | 0.432399 |
| PLCG1 | 0.046331 | 1.577943 | IRF5 | 0.002872 | 0.432571 |
| MFGE8 | 0.02708 | 1.57005 | IL18 | 0.001917 | 0.436314 |
| BMP2 | 0.021593 | 1.566266 | HCK | 0.001456 | 0.437248 |
| SMAD4 | 0.037501 | 1.564762 | EPRS | 0.007919 | 0.437758 |
| PTK2 | 0.025541 | 1.549441 | CHST1 | 0.020002 | 0.446299 |
| PDPK1 | 0.037973 | 1.549319 | CSF1R | 0.005968 | 0.446374 |
| TXNRD1 | 0.04184 | 1.533735 | LYL1 | 0.01192 | 0.450847 |
| CCK | 0.025606 | 1.533267 | LTB | 0.003742 | 0.45128 |
| H1f0 | 0.049937 | 1.52435 | NME4 | 0.002024 | 0.4515 |
| RASSF1 | 0.016471 | 1.523397 | EIF2AK2 | 0.029867 | 0.454197 |
| IRGQ | 0.029731 | 1.522762 | ITGA6 | 0.004994 | 0.461153 |
| ITPR1 | 0.019697 | 1.520793 | IL1B | 0.005607 | 0.462555 |
| Tpm1 | 0.018837 | 1.505396 | CCL20 | 0.01965 | 0.463194 |
| HERPUD1 | 0.01871 | 1.499705 | RGS1 | 0.030743 | 0.463314 |
| PCF11 | 0.035221 | 1.490868 | JUN | 0.008038 | 0.463788 |
| PHF19 | 0.039847 | 1.489077 | PPP1R3C | 0.004579 | 0.465449 |
| ID3 | 0.022863 | 1.48665 | CAMP | 0.003696 | 0.466537 |
| EXOC3L4 | 0.039565 | 1.47558 | HGF | 0.022724 | 0.468125 |
| TRPS1 | 0.047678 | 1.472734 | PTPN6 | 0.001817 | 0.468738 |
| ARHGEF1 | 0.046517 | 1.467499 | DOK3 | 0.001966 | 0.469021 |
| CKB | 0.034141 | 1.45806 | CASP4 | 0.003075 | 0.471221 |
| NFAT5 | 0.038162 | 1.441903 | PPCDC | 0.006313 | 0.472589 |
| MMP14 | 0.033165 | 1.432911 | SLC16A6 | 0.020129 | 0.479146 |
| MXD1 | 0.047756 | 1.423073 | SPRY2 | 0.030253 | 0.480036 |
| ICAM1 | 0.042874 | 1.420584 | CREG1 | 0.003955 | 0.481058 |
| STRN3 | 0.049631 | 1.416667 | Samd9l | 0.005618 | 0.490572 |
| ILK | 0.038266 | 1.394604 | DUSP6 | 0.00531 | 0.498799 |
|  |  |  | IGFBP4 | 0.005075 | 0.503478 |
|  |  |  | AXL | 0.003536 | 0.513924 |
|  |  |  | CCND1 | 0.003024 | 0.514186 |
|  |  |  | MSR1 | 0.009626 | 0.518348 |
|  |  |  | CDK6 | 0.041562 | 0.518391 |
|  |  |  | C5AR1 | 0.006584 | 0.518878 |
|  |  |  | CD68 | 0.002243 | 0.518982 |
|  |  |  | APOE | 0.003838 | 0.524429 |
|  |  |  | PTPRJ | 0.023463 | 0.528698 |
|  |  |  | Rps6ka5 | 0.00753 | 0.536178 |
|  |  |  | PSMB9 | 0.013015 | 0.541892 |
|  |  |  | RFFL | 0.014098 | 0.548869 |
|  |  |  | CD14 | 0.007801 | 0.552242 |
|  |  |  | CDK1 | 0.007484 | 0.553842 |
|  |  |  | ATF3 | 0.008856 | 0.555426 |
|  |  |  | CD47 | 0.012838 | 0.556633 |
|  |  |  | APOBR | 0.013557 | 0.559386 |
|  |  |  | TLR6 | 0.008641 | 0.559643 |
|  |  |  | CCL11 | 0.034165 | 0.566904 |
|  |  |  | PELI2 | 0.041637 | 0.567311 |
|  |  |  | IFI30 | 0.020445 | 0.569626 |
|  |  |  | THBD | 0.011003 | 0.571641 |
|  |  |  | INHBA | 0.019261 | 0.571781 |
|  |  |  | TMEM140 | 0.007256 | 0.57356 |
|  |  |  | PLSCR1 | 0.010427 | 0.577697 |
|  |  |  | KDELC2 | 0.017431 | 0.579066 |
|  |  |  | NMI | 0.017029 | 0.579654 |
|  |  |  | VAT1 | 0.011607 | 0.585039 |
|  |  |  | CFD | 0.010446 | 0.58525 |
|  |  |  | GBP2 | 0.0134 | 0.585695 |
|  |  |  | TCF19 | 0.011495 | 0.588895 |
|  |  |  | SH3BP5 | 0.021564 | 0.589112 |
|  |  |  | UCP2 | 0.037364 | 0.595358 |
|  |  |  | UCHL3 | 0.019834 | 0.596802 |
|  |  |  | Slfn2 | 0.011519 | 0.598708 |
|  |  |  | APOBEC1 | 0.01338 | 0.600499 |
|  |  |  | MAF | 0.015039 | 0.601677 |
|  |  |  | SET | 0.020592 | 0.601838 |
|  |  |  | MDP1 | 0.026734 | 0.604906 |
|  |  |  | NOS2 | 0.029067 | 0.609919 |
|  |  |  | CDC42EP2 | 0.02622 | 0.609995 |

Part 3 (Continued…)

| upregulated gene (N=108) | | | downregulated gene (N=182) | | |
| --- | --- | --- | --- | --- | --- |
| Symbol | p value | FC | Symbol | p value | FC |
|  |  |  | PTK2B | 0.014683 | 0.612083 |
|  |  |  | WARS | 0.010235 | 0.615468 |
|  |  |  | DRAM2 | 0.026318 | 0.616251 |
|  |  |  | MAP2K7 | 0.015 | 0.616519 |
|  |  |  | PLK3 | 0.014415 | 0.621823 |
|  |  |  | TRADD | 0.047163 | 0.622141 |
|  |  |  | DUT | 0.011701 | 0.622491 |
|  |  |  | EPS15 | 0.037508 | 0.622818 |
|  |  |  | LYN | 0.023731 | 0.623521 |
|  |  |  | IFNGR1 | 0.014862 | 0.626984 |
|  |  |  | MAP3K3 | 0.01627 | 0.630149 |
|  |  |  | RNF166 | 0.041258 | 0.631196 |
|  |  |  | AKAP10 | 0.023834 | 0.631394 |
|  |  |  | ITGAM | 0.026775 | 0.635182 |
|  |  |  | MTSS1 | 0.027493 | 0.636087 |
|  |  |  | ITPKB | 0.042047 | 0.636941 |
|  |  |  | LIPA | 0.015324 | 0.640493 |
|  |  |  | SEC14L1 | 0.042418 | 0.645835 |
|  |  |  | CASP1 | 0.01913 | 0.650703 |
|  |  |  | MOSPD2 | 0.045068 | 0.656837 |
|  |  |  | IFITM2 | 0.030635 | 0.675157 |
|  |  |  | DCBLD2 | 0.043076 | 0.675572 |
|  |  |  | BID | 0.049877 | 0.680013 |
|  |  |  | RNF213 | 0.042251 | 0.680268 |
|  |  |  | SAMSN1 | 0.03501 | 0.68253 |
|  |  |  | BCL10 | 0.043766 | 0.68295 |
|  |  |  | ZMYND8 | 0.040272 | 0.690336 |
|  |  |  | SCAP | 0.041817 | 0.691825 |
|  |  |  | CD48 | 0.041512 | 0.701707 |
|  |  |  | VPS29 | 0.039716 | 0.704564 |
|  |  |  | DOCK8 | 0.035803 | 0.708191 |
|  |  |  | MAD2L1 | 0.041284 | 0.708427 |
|  |  |  | NR1H3 | 0.043372 | 0.708571 |

**Supplemental Table 4. The static or oscillatory shear stress conditions upregulated eight LIUS-upregulated innatomic genes including 2 genes (out of 77) in lymphoma cells, one (out of 21) in preosteoblasts and five (out of 108) in bone marrow cells.** In addition, the static or oscillatory shear stress conditions downregulate eight LIUS-upregulated innatomic genes including 2 (out of 77) genes in lymphoma cells, 3 (out of 21) in preosteoblasts and 2 (out of 108) in bone marrow cells. Moreover, the static or oscillatory shear stress conditions upregulate 10 LIUS-downregulated innatomic genes including 3 genes (out of 39) in lymphoma cells, one (out of 17) in preosteoblasts and 6 (out of 182) in bone marrow cells. Finally, the static or oscillatory shear stress conditions downregulate 14 LIUS-downregulated innatomic genes including 1 gene (out of 17) in preosteoblasts, and 13 (out of 182) in bone marrow cells. These results suggest that LIUS may partially fulfill its therapeutic effects via static or oscillatory shear stress mechanisms.

| Treatment | Static or oscillatory shear stress conditions | | | | | | | | |
| --- | --- | --- | --- | --- | --- | --- | --- | --- | --- |
| GEO ID | GSE60152 | | | | | | | | |
|  | LIUS-upregulated gene | | | |  | LIUS-downregulated gene | | | |
| Modulation | Gene | P. value | Fold Change | Cell Type | Trend | Gene | P. value | Fold Change | Cell Type |
| Upregulated | DUSP4 | 0.05 | 1.53 | L | Upregulated | MTSS1 | 0.01 | 1.59 | L |
| 8 | CCR1 | 0.04 | 1.32 | L | 10 | Tnik | 0.01 | 1.56 | L |
|  | RGS3 | 0.03 | 1.42 | P |  | RGS3 | 0.03 | 1.42 | L |
|  | ENC1 | 0.00 | 2.65 | B |  | EDN1 | 0.00 | 2.21 | P |
|  | SLC4A7 | 0.02 | 1.67 | B |  | MTSS1 | 0.01 | 1.59 | B |
|  | BCAR1 | 0.02 | 1.55 | B |  | DUSP4 | 0.05 | 1.53 | B |
|  | RGS3 | 0.03 | 1.42 | B |  | ADAM9 | 0.02 | 1.46 | B |
|  | METRNL | 0.04 | 1.30 | B |  | SLC30A1 | 0.02 | 1.43 | B |
|  |  |  |  |  |  | ITPKB | 0.03 | 1.33 | B |
|  |  |  |  |  |  | CCR1 | 0.04 | 1.32 | B |
| Downregulated | PDGFRL | 0.02 | 0.72 | B | Downregulated | HMMR | 0.00 | 0.46 | P |
| 8 | HERPUD1 | 0.01 | 0.64 | B | 14 | KDELC2 | 0.04 | 0.76 | B |
|  | DDR2 | 0.02 | 0.55 | B |  | ITGB2 | 0.03 | 0.76 | B |
|  | FTH1 | 0.05 | 0.78 | L |  | IFNGR1 | 0.03 | 0.70 | B |
|  | RGS2 | 0.03 | 0.56 | L |  | PELI2 | 0.01 | 0.68 | B |
|  | VEGFC | 0.04 | 0.68 | P |  | CD83 | 0.05 | 0.65 | B |
|  | RGS2 | 0.03 | 0.56 | P |  | TCF19 | 0.03 | 0.65 | B |
|  | CH25H | 0.01 | 0.50 | P |  | NEIL3 | 0.04 | 0.62 | B |
|  |  |  |  |  |  | MAD2L1 | 0.04 | 0.61 | B |
|  |  |  |  |  |  | SAT1 | 0.04 | 0.60 | B |
|  |  |  |  |  |  | RRM2 | 0.02 | 0.58 | B |
|  |  |  |  |  |  | CDK1 | 0.00 | 0.53 | B |
|  |  |  |  |  |  | CH25H | 0.01 | 0.50 | B |
|  |  |  |  |  |  | GBP2 | 0.00 | 0.45 | B |

**Supplemental Table 5. The 82 heat shock proteins in heat shock family are classified into 4 groups including: a) heat shock 90kDa proteins (5); b) DNAJ (HSP40) heat shock proteins (49), c) small heat shock proteins (11), and d) heat shock 70kDa proteins (17).** Since the eukaryotic heat shock response is an ancient and highly conserved transcriptional program that results in the immediate synthesis of a battery of cytoprotective genes in the presence of thermal and other environmental stresses (PMID: 22688810), we examined heat shock protein gene expressions in LIUS-treated cell types to determine whether LIUS treatments trigger heat shock responses.

| Group | Gene symbol | Gene name |
| --- | --- | --- |
| Heat shock 90kDa proteins | HSP90AA1 | heat shock protein 90 alpha family class A member 1 |
| Heat shock 90kDa proteins | HSP90AA3P | heat shock protein 90 alpha family class A member 3, pseudogene |
| Heat shock 90kDa proteins | HSP90AB1 | heat shock protein 90 alpha family class B member 1 |
| Heat shock 90kDa proteins | HSP90B1 | heat shock protein 90 beta family member 1 |
| Heat shock 90kDa proteins | TRAP1 | TNF receptor associated protein 1 |
| DNAJ (HSP40) heat shock proteins | DNAJA1 | DnaJ heat shock protein family (Hsp40) member A1 |
| DNAJ (HSP40) heat shock proteins | DNAJA2 | DnaJ heat shock protein family (Hsp40) member A2 |
| DNAJ (HSP40) heat shock proteins | DNAJA3 | DnaJ heat shock protein family (Hsp40) member A3 |
| DNAJ (HSP40) heat shock proteins | DNAJA4 | DnaJ heat shock protein family (Hsp40) member A4 |
| DNAJ (HSP40) heat shock proteins | DNAJB1 | DnaJ heat shock protein family (Hsp40) member B1 |
| DNAJ (HSP40) heat shock proteins | DNAJB2 | DnaJ heat shock protein family (Hsp40) member B2 |
| DNAJ (HSP40) heat shock proteins | DNAJB3 | DnaJ heat shock protein family (Hsp40) member B3 |
| DNAJ (HSP40) heat shock proteins | DNAJB4 | DnaJ heat shock protein family (Hsp40) member B4 |
| DNAJ (HSP40) heat shock proteins | DNAJB5 | DnaJ heat shock protein family (Hsp40) member B5 |
| DNAJ (HSP40) heat shock proteins | DNAJB6 | DnaJ heat shock protein family (Hsp40) member B6 |
| DNAJ (HSP40) heat shock proteins | DNAJB7 | DnaJ heat shock protein family (Hsp40) member B7 |
| DNAJ (HSP40) heat shock proteins | DNAJB8 | DnaJ heat shock protein family (Hsp40) member B8 |
| DNAJ (HSP40) heat shock proteins | DNAJB9 | DnaJ heat shock protein family (Hsp40) member B9 |
| DNAJ (HSP40) heat shock proteins | DNAJB11 | DnaJ heat shock protein family (Hsp40) member B11 |
| DNAJ (HSP40) heat shock proteins | DNAJB12 | DnaJ heat shock protein family (Hsp40) member B12 |
| DNAJ (HSP40) heat shock proteins | DNAJB13 | DnaJ heat shock protein family (Hsp40) member B13 |
| DNAJ (HSP40) heat shock proteins | DNAJB14 | DnaJ heat shock protein family (Hsp40) member B14 |
| DNAJ (HSP40) heat shock proteins | DNAJC1 | DnaJ heat shock protein family (Hsp40) member C1 |
| DNAJ (HSP40) heat shock proteins | DNAJC2 | DnaJ heat shock protein family (Hsp40) member C2 |
| DNAJ (HSP40) heat shock proteins | DNAJC3 | DnaJ heat shock protein family (Hsp40) member C3 |
| DNAJ (HSP40) heat shock proteins | DNAJC4 | DnaJ heat shock protein family (Hsp40) member C4 |
| DNAJ (HSP40) heat shock proteins | DNAJC5 | DnaJ heat shock protein family (Hsp40) member C5 |
| DNAJ (HSP40) heat shock proteins | DNAJC5B | DnaJ heat shock protein family (Hsp40) member C5 beta |
| DNAJ (HSP40) heat shock proteins | DNAJC5G | DnaJ heat shock protein family (Hsp40) member C5 gamma |
| DNAJ (HSP40) heat shock proteins | DNAJC6 | DnaJ heat shock protein family (Hsp40) member C6 |
| DNAJ (HSP40) heat shock proteins | DNAJC7 | DnaJ heat shock protein family (Hsp40) member C7 |
| DNAJ (HSP40) heat shock proteins | DNAJC8 | DnaJ heat shock protein family (Hsp40) member C8 |
| DNAJ (HSP40) heat shock proteins | DNAJC9 | DnaJ heat shock protein family (Hsp40) member C9 |
| DNAJ (HSP40) heat shock proteins | DNAJC10 | DnaJ heat shock protein family (Hsp40) member C10 |
| DNAJ (HSP40) heat shock proteins | DNAJC11 | DnaJ heat shock protein family (Hsp40) member C11 |
| DNAJ (HSP40) heat shock proteins | DNAJC12 | DnaJ heat shock protein family (Hsp40) member C12 |
| DNAJ (HSP40) heat shock proteins | DNAJC13 | DnaJ heat shock protein family (Hsp40) member C13 |
| DNAJ (HSP40) heat shock proteins | DNAJC14 | DnaJ heat shock protein family (Hsp40) member C14 |
| DNAJ (HSP40) heat shock proteins | DNAJC15 | DnaJ heat shock protein family (Hsp40) member C15 |
| DNAJ (HSP40) heat shock proteins | DNAJC16 | DnaJ heat shock protein family (Hsp40) member C16 |
| DNAJ (HSP40) heat shock proteins | DNAJC17 | DnaJ heat shock protein family (Hsp40) member C17 |
| DNAJ (HSP40) heat shock proteins | DNAJC18 | DnaJ heat shock protein family (Hsp40) member C18 |
| DNAJ (HSP40) heat shock proteins | DNAJC19 | DnaJ heat shock protein family (Hsp40) member C19 |
| DNAJ (HSP40) heat shock proteins | HSCB | HscB mitochondrial iron-sulfur cluster cochaperone |
| DNAJ (HSP40) heat shock proteins | DNAJC21 | DnaJ heat shock protein family (Hsp40) member C21 |
| DNAJ (HSP40) heat shock proteins | DNAJC22 | DnaJ heat shock protein family (Hsp40) member C22 |
| DNAJ (HSP40) heat shock proteins | SEC63 | SEC63 homolog, protein translocation regulator |
| DNAJ (HSP40) heat shock proteins | DNAJC24 | DnaJ heat shock protein family (Hsp40) member C24 |
| DNAJ (HSP40) heat shock proteins | DNAJC25 | DnaJ heat shock protein family (Hsp40) member C25 |
| DNAJ (HSP40) heat shock proteins | GAK | cyclin G associated kinase |
| DNAJ (HSP40) heat shock proteins | DNAJC27 | DnaJ heat shock protein family (Hsp40) member C27 |
| DNAJ (HSP40) heat shock proteins | DNAJC28 | DnaJ heat shock protein family (Hsp40) member C28 |
| DNAJ (HSP40) heat shock proteins | SACS | sacsin molecular chaperone |
| DNAJ (HSP40) heat shock proteins | DNAJC30 | DnaJ heat shock protein family (Hsp40) member C30 |
| Small heat shock proteins | HSPB1 | heat shock protein family B (small) member 1 |
| Small heat shock proteins | HSPB2 | heat shock protein family B (small) member 2 |
| Small heat shock proteins | HSPB3 | heat shock protein family B (small) member 3 |
| Small heat shock proteins | CRYAA | crystallin alpha A |
| Small heat shock proteins | CRYAB | crystallin alpha B |
| Small heat shock proteins | HSPB6 | heat shock protein family B (small) member 6 |
| Small heat shock proteins | HSPB7 | heat shock protein family B (small) member 7 |
| Small heat shock proteins | HSPB8 | heat shock protein family B (small) member 8 |
| Small heat shock proteins | HSPB9 | heat shock protein family B (small) member 9 |
| Small heat shock proteins | ODF1 | outer dense fiber of sperm tails 1 |
| Small heat shock proteins | HSPB11 | heat shock protein family B (small) member 11 |
| Heat shock 70kDa proteins | HSPA1A | heat shock protein family A (Hsp70) member 1A |
| Heat shock 70kDa proteins | HSPA1B | heat shock protein family A (Hsp70) member 1B |
| Heat shock 70kDa proteins | HSPA1L | heat shock protein family A (Hsp70) member 1 like |
| Heat shock 70kDa proteins | HSPA2 | heat shock protein family A (Hsp70) member 2 |
| Heat shock 70kDa proteins | HSPA4 | heat shock protein family A (Hsp70) member 4 |
| Heat shock 70kDa proteins | HSPA4L | heat shock protein family A (Hsp70) member 4 like |
| Heat shock 70kDa proteins | HSPA5 | heat shock protein family A (Hsp70) member 5 |
| Heat shock 70kDa proteins | HSPA6 | heat shock protein family A (Hsp70) member 6 |
| Heat shock 70kDa proteins | HSPA7 | heat shock protein family A (Hsp70) member 7 |
| Heat shock 70kDa proteins | HSPA8 | heat shock protein family A (Hsp70) member 8 |
| Heat shock 70kDa proteins | HSPA9 | heat shock protein family A (Hsp70) member 9 |
| Heat shock 70kDa proteins | HSPA12A | heat shock protein family A (Hsp70) member 12A |
| Heat shock 70kDa proteins | HSPA12B | heat shock protein family A (Hsp70) member 12B |
| Heat shock 70kDa proteins | HSPA13 | heat shock protein family A (Hsp70) member 13 |
| Heat shock 70kDa proteins | HSPA14 | heat shock protein family A (Hsp70) member 14 |
| Heat shock 70kDa proteins | HSPH1 | heat shock protein family H (Hsp110) member 1 |
| Heat shock 70kDa proteins | HYOU1 | hypoxia up-regulated 1 |

**Supplemental Table 6. Low intensity ultrasound (LIUS) upregulated heat shock protein expressions in lymphoma cells but downregulated heat shock protein expressions in non-cancer cells.** Our results showed that LIUS modulates the expressions of 5 out of 82 heat shock proteins in human lymphoma cells (3 increased, and 2 decreased). LIUS downregulated two heat shock proteins in mouse preosteoblasts and downregulated 7 heat shock proteins in mouse bone marrow cells.

|  | Group | Symbol | P. Value | FC |
| --- | --- | --- | --- | --- |
| GSE10212  Human lymphoma | DNAJ (HSP40) heat shock proteins | GAK | 0.02 | 6.78 |
|  | DNAJ (HSP40) heat shock proteins | DNAJB12 | 0.03 | 1.58 |
|  | Heat shock 70kDa proteins | HSPA6 | 0.04 | 1.55 |
|  | DNAJ (HSP40) heat shock proteins | DNAJC7 | 0.03 | 0.57 |
|  | Small heat shock proteins | HSPB3 | 0.04 | 0.44 |
| GSE45487  Mouse preosteoblasts | Heat shock 70kDa proteins | HSPA4L | 0.03 | 0.71 |
|  | Heat shock 70kDa proteins | HSPH1 | 0.04 | 0.75 |
| GSE70662  Rat bone marrow cells | DNAJ (HSP40) heat shock proteins | DNAJB13 | 0.00 | 0.10 |
|  | DNAJ (HSP40) heat shock proteins | DNAJC5G | 0.00 | 0.13 |
|  | DNAJ (HSP40) heat shock proteins | SACS | 0.00 | 0.22 |
|  | Small heat shock proteins | HSPB11 | 0.01 | 0.43 |
|  | Small heat shock proteins | CRYAB | 0.01 | 0.59 |
|  | Small heat shock proteins | HSPB7 | 0.04 | 0.65 |
|  | Heat shock 70kDa proteins | HSPA1B | 0.01 | 0.53 |

**Supplemental Table 7. The mild hyperthermia treatment (41** °C**) upregulated 15 LIUS-upregulated innatomic genes in fibroblast OUMS-36 cells including 6 genes in lymphoma cells (L), 2 genes in preosteoblast cells and 7 genes in bone marrow cells**. In addition, the mild hyperthermia treatment downregulate 6 LIUS-upregulated innatomic genes including 5 genes in lymphoma cells, and 1 in bone marrow cells. Moreover, the mild hyperthermia treatment upregulate 20 LIUS-downregulated innatomic genes including 4 genes in lymphoma cells, 3 in preosteoblast cells and 13 in bone marrow cells. Finally, the mild hyperthermia treatment downregulate 11 LIUS-downregulated innatomic genes including 2 genes in lymphoma cells and preosteoblasts, and 9 in bone marrow cells. These results suggest that LIUS may partially fulfill its therapeutic effects via heat-generated mechanisms.

| Treatment | Mild hyperthermia (41 °C) in Fibroblast OUMS-36 cells | | | | | | | | |
| --- | --- | --- | --- | --- | --- | --- | --- | --- | --- |
| GEO ID | GSE39178 | | | | | | | | |
|  | LIUS-upregulated gene | | | |  | LIUS-downregulated gene | | | |
| Modulation | Gene | P. value | Fold Change | Cell Type | Modulation | Gene | P. value | Fold Change | Cell Type |
| Upregulated | NR4A3 | 0.02 | 8.46 | L | Upregulated | BLNK | 0.00 | 14.93 | P |
| 15 | APOB | 0.03 | 4.41 | L | 20 | IL1RN | 0.04 | 5.17 | P |
|  | LAMC3 | 0.05 | 4.14 | L |  | FABP4 | 0.03 | 3.10 | P |
|  | DUSP4 | 0.03 | 4.11 | L |  | TLR4 | 0.01 | 17.75 | B |
|  | Rasal2 | 0.02 | 3.41 | L |  | SYK | 0.02 | 9.71 | B |
|  | CHAC1 | 0.03 | 3.46 | B |  | MYT1 | 0.02 | 9.19 | B |
|  | HMOX1 | 0.04 | 2.57 | L |  | CD48 | 0.02 | 6.87 | B |
|  | Tpm1 | 0.01 | 6.32 | P |  | LMO2 | 0.01 | 6.15 | B |
|  | CH25H | 0.01 | 4.47 | P |  | WAS | 0.01 | 4.92 | B |
|  | IRGQ | 0.00 | 22.01 | B |  | CH25H | 0.01 | 4.47 | B |
|  | BLNK | 0.00 | 14.93 | B |  | APOB | 0.03 | 4.41 | B |
|  | Tpm1 | 0.01 | 6.32 | B |  | LAMC3 | 0.05 | 4.14 | B |
|  | PCDHB7 | 0.04 | 4.35 | B |  | DUSP4 | 0.03 | 4.11 | B |
|  | Pcdhb7 | 0.04 | 4.35 | B |  | ARHGAP25 | 0.03 | 3.58 | B |
|  | ZBTB10 | 0.03 | 3.56 | B |  | APOBEC1 | 0.04 | 3.43 | B |
|  |  |  |  |  |  | HMOX1 | 0.04 | 2.57 | B |
|  |  |  |  |  |  | PTPN11 | 0.03 | 9.45 | L |
|  |  |  |  |  |  | SRC | 0.01 | 5.43 | L |
|  |  |  |  |  |  | AFF1 | 0.04 | 3.56 | L |
|  |  |  |  |  |  | CHAC1 | 0.03 | 3.46 | L |
| Downregulated | DLX3 | 0.03 | 0.17 | B | Downregulated | CD47 | 0.01 | 0.15 | L |
| 6 | RGS1 | 0.05 | 0.33 | L | 11 | SST | 0.01 | 0.07 | L |
|  | CD40 | 0.03 | 0.22 | L |  | RGS1 | 0.05 | 0.33 | B |
|  | ZFP36L1 | 0.01 | 0.18 | L |  | PELI2 | 0.03 | 0.29 | B |
|  | MERTK | 0.03 | 0.12 | L |  | RSAD2 | 0.01 | 0.19 | B |
|  | EZR | 0.01 | 0.06 | L |  | CD47 | 0.01 | 0.15 | B |
|  |  |  |  |  |  | NLRP3 | 0.03 | 0.12 | B |
|  |  |  |  |  |  | ELOVL3 | 0.01 | 0.10 | B |
|  |  |  |  |  |  | Samd9l | 0.01 | 0.08 | B |
|  |  |  |  |  |  | FCGR1A | 0.00 | 0.07 | B |
|  |  |  |  |  |  | CD14 | 0.03 | 0.07 | B |

**Supplemental Table 8. The mild hyperthermia treatment (41** °C**) upregulated 45 LIUS-upregulated innatomic genes in human lymphoma U937 cells including 20 genes (out of 77, 26%) in lymphoma cells (L), 6 (out of 21, 28.6%)) in preosteoblasts, and 19 (out of 108, 17.6%) in bone marrow cells**. In addition, the mild hyperthermia treatment downregulated 22 LIUS-upregulated innatomic genes including 12 genes in lymphoma cells, and 10 in bone marrow cells. Moreover, the mild hyperthermia treatment upregulated 20 LIUS-downregulated innatomic genes including 4 genes in lymphoma cells, 2 in preosteoblast cells and 14 in bone marrow cells. Finally, the mild hyperthermia treatment downregulated 24 LIUS-downregulated innatomic genes including 8 genes in lymphoma cells, 1 in preosteoblast cells, and 15 in bone marrow cells. These results suggest that LIUS may partially fulfill its therapeutic effects via heat-generated mechanisms.

| Treatment | Mild hyperthermia (41 °C) in human lymphoma U937 cells | | | | | | | | |
| --- | --- | --- | --- | --- | --- | --- | --- | --- | --- |
| GEO ID | GSE10043 | | | | | | | | |
|  | LIUS-upregulated gene | | | |  | LIUS-downregulated gene | | | |
| Modulation | Gene | P. value | Fold Change | Cell Type | Trend | Gene | P. value | Fold Change | Cell Type |
| Upregulated | SERPINE1 | 0.00 | 22.60 | P | Upregulated | ITGB1 | 0.00 | 10.41 | L |
| 45 | TFPI | 0.00 | 12.54 | P | 20 | GJA1 | 0.01 | 8.74 | L |
|  | DBP | 0.00 | 3.04 | P |  | CCNG2 | 0.01 | 1.89 | L |
|  | RGS2 | 0.00 | 2.93 | P |  | ABTB2 | 0.03 | 1.53 | L |
|  | LMCD1 | 0.03 | 1.54 | P |  | IL1RN | 0.03 | 4.76 | P |
|  | Tpm1 | 0.04 | 1.46 | P |  | Pmaip1 | 0.03 | 1.95 | P |
|  | SERPINE1 | 0.00 | 22.60 | L |  | C3 | 0.01 | 21.08 | B |
|  | C3 | 0.01 | 21.08 | L |  | IL7R | 0.00 | 11.28 | B |
|  | IL7R | 0.00 | 11.28 | L |  | INHBA | 0.00 | 6.78 | B |
|  | HMOX1 | 0.00 | 5.53 | L |  | HMOX1 | 0.00 | 5.53 | B |
|  | PHLDA1 | 0.01 | 4.64 | L |  | IL10 | 0.04 | 3.53 | B |
|  | ELL2 | 0.02 | 4.39 | L |  | SFTPC | 0.05 | 2.86 | B |
|  | IL10 | 0.04 | 3.53 | L |  | PELI2 | 0.00 | 2.21 | B |
|  | RGS2 | 0.00 | 2.93 | L |  | CASP1 | 0.01 | 2.18 | B |
|  | MDM2 | 0.04 | 2.90 | L |  | CD163 | 0.02 | 2.13 | B |
|  | F3 | 0.04 | 2.64 | L |  | IL1B | 0.02 | 1.92 | B |
|  | ZFP36L1 | 0.02 | 2.61 | L |  | RGS1 | 0.04 | 1.85 | B |
|  | SGK1 | 0.01 | 2.42 | L |  | DUSP6 | 0.05 | 1.77 | B |
|  | EZR | 0.02 | 2.27 | L |  | SAT1 | 0.03 | 1.72 | B |
|  | IL2RB | 0.04 | 2.26 | L |  | SLC30A1 | 0.03 | 1.60 | B |
|  | C18orf25 | 0.02 | 2.18 | L |  |  |  |  |  |
|  | IER3 | 0.01 | 1.97 | L |  |  |  |  |  |
|  | GDF15 | 0.02 | 1.96 | L |  |  |  |  |  |
|  | RGS1 | 0.04 | 1.85 | L |  |  |  |  |  |
|  | SLC2A1 | 0.03 | 1.64 | L |  |  |  |  |  |
|  | KLF6 | 0.04 | 1.48 | L |  |  |  |  |  |
|  | PLK2 | 0.01 | 10.19 | B |  |  |  |  |  |
|  | MXD1 | 0.04 | 9.82 | B |  |  |  |  |  |
|  | GJA1 | 0.01 | 8.74 | B |  |  |  |  |  |
|  | IER5 | 0.00 | 6.86 | B |  |  |  |  |  |
|  | PLOD2 | 0.03 | 6.79 | B |  |  |  |  |  |
|  | PDGFRL | 0.01 | 6.03 | B |  |  |  |  |  |
|  | PARVA | 0.00 | 5.20 | B |  |  |  |  |  |
|  | PHLDA1 | 0.01 | 4.64 | B |  |  |  |  |  |
|  | ELL2 | 0.02 | 4.39 | B |  |  |  |  |  |
|  | SDC2 | 0.01 | 3.73 | B |  |  |  |  |  |
|  | MDM2 | 0.04 | 2.90 | B |  |  |  |  |  |
|  | SLC38A2 | 0.01 | 2.12 | B |  |  |  |  |  |
|  | ENC1 | 0.01 | 2.09 | B |  |  |  |  |  |
|  | CCNG2 | 0.01 | 1.89 | B |  |  |  |  |  |
|  | TBPL1 | 0.01 | 1.84 | B |  |  |  |  |  |
|  | SLC4A7 | 0.02 | 1.81 | B |  |  |  |  |  |
|  | COQ10B | 0.04 | 1.62 | B |  |  |  |  |  |
|  | GADD45B | 0.05 | 1.60 | B |  |  |  |  |  |
|  | Tpm1 | 0.04 | 1.46 | B |  |  |  |  |  |
| Downregulated | DYRK2 | 0.05 | 0.63 | B | Downregulated | CHAC1 | 0.03 | 0.57 | L |
| 22 | FUBP1 | 0.02 | 0.61 | B | 24 | APBB2 | 0.02 | 0.53 | L |
|  | CHAC1 | 0.03 | 0.57 | B |  | SRC | 0.04 | 0.52 | L |
|  | IRGQ | 0.03 | 0.56 | B |  | NFE2 | 0.01 | 0.51 | L |
|  | NEDD9 | 0.02 | 0.53 | B |  | DUSP2 | 0.01 | 0.50 | L |
|  | BMP2 | 0.03 | 0.47 | B |  | EZH1 | 0.01 | 0.49 | L |
|  | GSPT1 | 0.01 | 0.44 | B |  | Pvr | 0.02 | 0.45 | L |
|  | SMAD4 | 0.03 | 0.43 | B |  | TLR7 | 0.01 | 0.17 | L |
|  | HOMER2 | 0.04 | 0.25 | B |  | HOMER2 | 0.04 | 0.25 | P |
|  | ZBTB10 | 0.00 | 0.03 | B |  | TROVE2 | 0.03 | 0.66 | B |
|  | FOSL2 | 0.05 | 0.65 | L |  | GCA | 0.02 | 0.62 | B |
|  | CDK12 | 0.05 | 0.53 | L |  | ZMYND8 | 0.04 | 0.57 | B |
|  | IL1RL1 | 0.04 | 0.48 | L |  | IFNGR1 | 0.04 | 0.51 | B |
|  | BMP2 | 0.03 | 0.47 | L |  | IRF4 | 0.01 | 0.48 | B |
|  | ITGB3 | 0.02 | 0.44 | L |  | IL1RL1 | 0.04 | 0.48 | B |
|  | NAB2 | 0.02 | 0.37 | L |  | EPS15 | 0.04 | 0.48 | B |
|  | Ccl2 | 0.00 | 0.33 | L |  | EGR1 | 0.01 | 0.45 | B |
|  | CCL2 | 0.00 | 0.33 | L |  | Ccl2 | 0.00 | 0.33 | B |
|  | ADCY2 | 0.02 | 0.31 | L |  | CCL2 | 0.00 | 0.33 | B |
|  | NOS2 | 0.00 | 0.27 | L |  | NOS2 | 0.00 | 0.27 | B |
|  | Sp100 | 0.01 | 0.26 | L |  | MAP2K7 | 0.00 | 0.26 | B |
|  | CD44 | 0.00 | 0.06 | L |  | MSR1 | 0.03 | 0.26 | B |
|  |  |  |  |  |  | TLR7 | 0.01 | 0.17 | B |
|  |  |  |  |  |  | HGF | 0.01 | 0.12 | B |

**Supplemental Table 9. By analyzing the microarray data from cytokine gene knock-out (KO) cells, or cytokine-treated cells, we found that LIUS-upregulated innatomic genes in human lymphoma cells can be modulated by a set of cytokines.** LIUS-upregulated innatomic genes are downregulated more than upregulated in proinflammatory cytokines tumor necrosis factor (TNF) KO cells (2 genes vs. 4), interleukin-6 (IL-6) KO cells (3 genes vs. 8), IL-1b KO (3 genes vs. 4), and anti-inflammatory cytokine TGFb-treated lung carcinoma cells (8 genes vs. 22). In addition, LIUS-upregulated innatomic genes are upregulated more than downregulated in anti-inflammatory cytokine IL-10 KO cells (5 genes vs. 3). These results suggest that LIUS treatment of cancer cells promote the innatomic gene expressions by enhancing proinflammatory cytokine pathways and inhibiting anti-inflammatory cytokine pathways.

| Array L |  |  |  |  |  |  |  |  |  |  |
| --- | --- | --- | --- | --- | --- | --- | --- | --- | --- | --- |
| Cytokine | Species | Tissue/cell type | comparison | number | | Up-regulated | Fold change | Down-regulated | Fold change | GEO ID |
|  |  |  |  | up | down |  |  |  |  |  |
| TNF | mouse | gastric tumors | TNF KO gastric tumor | 2 | 4 | SP100 | 2.38 | MMP14 | 0.72 | GSE43145 |
|  |  |  | vs. |  |  | ELL2 | 1.49 | CD44 | 0.64 |  |
|  |  |  | gastric tumor |  |  |  |  | PTX3 | 0.63 |  |
|  |  |  |  |  |  |  |  | EDNRB | 0.56 |  |
| IFN1A | mouse | intestinal epithelium | IFNAR1 KO intestinal epithelium | 6 | 4 | MFGE8 | 1.50 | CXCL10 | 0.85 | GSE76512 |
|  |  |  | vs. |  |  | MMP14 | 1.44 | EZR | 0.76 |  |
|  |  |  | Intestinal epithelium |  |  | ZFP36L1 | 1.31 | MERTK | 0.58 |  |
|  |  |  |  |  |  | GDF15 | 1.27 | C3 | 0.26 |  |
|  |  |  |  |  |  | SQSTM1 | 1.25 |  |  |  |
|  |  |  |  |  |  | DUSP4 | 1.20 |  |  |  |
| IL-6 | mouse | hypothalamus | IL-6 KO mice | 3 | 8 | FABP7 | 1.47 | GDF15 | 0.95 | GSE34673 |
|  |  |  | vs. |  |  | GSTM5 | 1.21 | MBD2 | 0.95 |  |
|  |  |  | wt mice |  |  | FTH1 | 1.07 | IL2RB | 0.94 |  |
|  |  |  |  |  |  |  |  | SCARB1 | 0.92 |  |
|  |  |  |  |  |  |  |  | LHX1 | 0.92 |  |
|  |  |  |  |  |  |  |  | EZR | 0.92 |  |
|  |  |  |  |  |  |  |  | NAB2 | 0.89 |  |
|  |  |  |  |  |  |  |  | FOSL2 | 0.86 |  |
| IL-1β | mouse | spinal cord | IL-1b-KO | 3 | 4 | RGS1 | 1.10 | S100A10 | 0.90 | GSE70302 |
|  |  |  | vs. |  |  | NR4A3 | 1.08 | MERTK | 0.90 |  |
|  |  |  | wt mice |  |  | FYN | 1.07 | SDC4 | 0.90 |  |
|  |  |  |  |  |  |  |  | EZR | 0.85 |  |
| IL-10 | mouse | lung | IL-10 KNOCK OUT | 5 | 3 | CCR1 | 1.46 | NR4A1 | 0.79 | GSE25095 |
|  |  |  | vs. |  |  | RGS2 | 1.36 | KLF6 | 0.78 |  |
|  |  |  | wt mice |  |  | CSF1 | 1.33 | SDC4 | 0.64 |  |
|  |  |  |  |  |  | CD44 | 1.27 |  |  |  |
|  |  |  |  |  |  | MBD2 | 1.20 |  |  |  |
| IL-35 | mouse | T cell | IL35_treated | 2 | 1 | APOB | 1.29 | Tsc22d3 | 0.68 | GSE24210 |
|  |  |  | vs. control |  |  | MERTK | 1.36 |  |  |  |
| TGF-β | human | A549 lung adenocarcinoma cell line | TGF-β1 treated | 8 | 22 | CCL20 | 2.19 | CDK12 | 0.95 | GSE17708 |
|  |  |  | vs. |  |  | GDF15 | 1.39 | Rasal2 | 0.94 |  |
|  |  |  | control |  |  | CSF1 | 1.23 | PCDH7 | 0.91 |  |
|  |  |  |  |  |  | DUSP4 | 1.17 | Tsc22d3 | 0.90 |  |
|  |  |  |  |  |  | SQSTM1 | 1.13 | Sp100 | 0.88 |  |
|  |  |  |  |  |  | MBD2 | 1.11 | ADM | 0.87 |  |
|  |  |  |  |  |  | MDM2 | 1.09 | NDEL1 | 0.85 |  |
|  |  |  |  |  |  | XIST | 1.06 | S100A10 | 0.83 |  |
|  |  |  |  |  |  |  |  | MAFK | 0.83 |  |
|  |  |  |  |  |  |  |  | SLC2A1 | 0.83 |  |
|  |  |  |  |  |  |  |  | IL7R | 0.82 |  |
|  |  |  |  |  |  |  |  | ZFP36L1 | 0.82 |  |
|  |  |  |  |  |  |  |  | SDC4 | 0.82 |  |
|  |  |  |  |  |  |  |  | Ccl2 | 0.79 |  |
|  |  |  |  |  |  |  |  | CCL2 | 0.79 |  |
|  |  |  |  |  |  |  |  | IER3 | 0.78 |  |
|  |  |  |  |  |  |  |  | HMOX1 | 0.74 |  |
|  |  |  |  |  |  |  |  | BMP2 | 0.69 |  |
|  |  |  |  |  |  |  |  | F3 | 0.67 |  |
|  |  |  |  |  |  |  |  | MAFB | 0.67 |  |
|  |  |  |  |  |  |  |  | JUN | 0.64 |  |
|  |  |  |  |  |  |  |  | SERPINE1 | 0.38 |  |
| TGF-β | human | immortalized ovarian surface epithelial cells | TGF-β1 treated | 15 | 14 | MAFB | 4.14 | ZFP36L1 | 0.81 | GSE6653 |
|  |  |  | vs. |  |  | PHLDA1 | 2.62 | CAPN2 | 0.80 |  |
|  |  |  | control |  |  | ADM | 2.51 | ELL2 | 0.79 |  |
|  |  |  |  |  |  | GDF15 | 1.69 | XIST | 0.78 |  |
|  |  |  |  |  |  | RGS2 | 1.62 | MMP14 | 0.77 |  |
|  |  |  |  |  |  | DUSP4 | 1.62 | NDEL1 | 0.76 |  |
|  |  |  |  |  |  | FYN | 1.62 | SGK1 | 0.74 |  |
|  |  |  |  |  |  | CSF1 | 1.59 | MAFK | 0.68 |  |
|  |  |  |  |  |  | SQSTM1 | 1.44 | C18orf25 | 0.67 |  |
|  |  |  |  |  |  | JUN | 1.40 | ITGB3 | 0.67 |  |
|  |  |  |  |  |  | FOSL2 | 1.40 | Rasal2 | 0.66 |  |
|  |  |  |  |  |  | MDM2 | 1.28 | SERPINE1 | 0.63 |  |
|  |  |  |  |  |  | SCARB1 | 1.28 | CD44 | 0.55 |  |
|  |  |  |  |  |  | MERTK | 1.28 | HMOX1 | 0.51 |  |
|  |  |  |  |  |  | MFGE8 | 1.22 |  |  |  |

**Supplemental Table 10. By analyzing the microarray data from cytokine gene KO cells, or cytokine-treated cells, we found that LIUS-downregulated innatomic genes in human lymphoma cells can be modulated by a set of cytokines**. LIUS-downregulated innatomic genes are upregulated more than downregulated in proinflammatory cytokines IL-6 KO cells (6 genes versus 5 genes), IL-1b KO (4 genes vs. 3 genes), and anti-inflammatory cytokine TGFb-treated lung carcinoma cells (12 genes vs. 7). In addition, LIUS-downregulated innatomic genes are upregulated more than downregulated in anti-inflammatory cytokine IL-10 KO cells (5 genes vs. 2). Once again, these results suggest that LIUS treatment of cancer cells promote the innatomic gene expressions by enhancing proinflammatory cytokine pathways and inhibiting anti-inflammatory cytokine pathways.

| Array L downregulate | | |  |  |  |  |  |  |  |  |
| --- | --- | --- | --- | --- | --- | --- | --- | --- | --- | --- |
| Cytokine | Species | Tissue/cell type | comparison | number | | Up-regulated gene | Fold change | Down-regulated gene | Fold change | GeoID |
|  |  |  |  | up | down |  |  |  |  |  |
| TNF | mouse | gastric tumors | TNF KO gastric tumor | 2 | 3 | EZH1 | 12.04 | ITGB1 | 0.57 | GSE43145 |
|  |  |  | vs. |  |  | SST | 3.38 | BHMT | 0.34 |  |
|  |  |  | gastric tumor |  |  |  |  | RGS3 | 0.24 |  |
| IFN1A | mouse | intestinal epithelium | IFNAR1 KO intestinal epithelium | 2 | 3 | MTSS1 | 1.25 | TLR1 | 0.80 | GSE76512 |
|  |  |  | vs. |  |  | SCN11A | 1.16 | CCNG2 | 0.78 |  |
|  |  |  | Intestinal epithelium |  | |  |  | ID1 | 0.74 |  |
| IL-6 | mouse | hypothalamus | IL-6 KO mice | 6 | 5 | RAB33A | 1.16 | MAPK8IP1 | 0.93 | GSE34673 |
|  |  |  | vs. |  |  | CD47 | 1.12 | CHAC1 | 0.93 |  |
|  |  |  | wt mice |  |  | ITGB1 | 1.10 | RGS3 | 0.92 |  |
|  |  |  |  |  |  | LPAR1 | 1.09 | DUSP2 | 0.92 |  |
|  |  |  |  |  |  | CCNG2 | 1.08 | ARHGDIA | 0.88 |  |
|  |  |  |  |  |  | EIF2B1 | 1.07 |  |  |  |
| IL-1β | mouse | spinal cord | IL-1b-KO | 4 | 3 | CCL19 | 1.16 | TLR1 | 0.93 | GSE70302 |
|  |  |  | vs. |  |  | ABTB2 | 1.15 | ZFP36L2 | 0.92 |  |
|  |  |  | wt mice |  |  | GJA1 | 1.10 | CCNG2 | 0.92 |  |
|  |  |  |  |  |  | RAB33A | 1.10 |  |  |  |
| IL-10 | mouse | lung | IL-10 KNOCK OUT | 5 | 2 | EZH1 | 1.38 | MTSS1 | 0.87 | GSE25095 |
|  |  |  | vs. |  |  | AFF1 | 1.19 | SRC | 0.82 |  |
|  |  |  | wt mice |  |  | LPAR1 | 1.19 |  |  |  |
|  |  |  |  |  |  | CLEC4E | 1.13 |  |  |  |
|  |  |  |  |  |  | RGS3 | 1.13 |  |  |  |
| IL-35 | mouse | T cell | IL35_treated | 0 | 2 |  |  | SST | 0.60 | GSE24210 |
|  |  |  | vs. control |  |  |  |  | RAB33A | 0.77 |  |
| TGF-β | human | A549 lung adenocarcinoma cell line | TGF-β1 treated | 12 | 7 | ID1 | 3.14 | ITGB1 | 0.84 | GSE17708 |
|  |  |  | vs. |  |  | ZFP36L2 | 1.89 | SCN11A | 0.84 |  |
|  |  |  | control |  |  | LPAR1 | 1.67 | PTPN11 | 0.80 |  |
|  |  |  |  |  |  | CSF3 | 1.66 | APBB2 | 0.78 |  |
|  |  |  |  |  |  | ARHGDIA | 1.34 | GJA1 | 0.75 |  |
|  |  |  |  |  |  | PML | 1.34 | RGS3 | 0.72 |  |
|  |  |  |  |  |  | ABTB2 | 1.31 | Pvr | 0.47 |  |
|  |  |  |  |  |  | BBX | 1.31 |  |  |  |
|  |  |  |  |  |  | TLR1 | 1.30 |  |  |  |
|  |  |  |  |  |  | EZH1 | 1.27 |  |  |  |
|  |  |  |  |  |  | CD47 | 1.27 |  |  |  |
|  |  |  |  |  |  | MTSS1 | 1.26 |  |  |  |
| TGF-β | human | immortalized ovarian surface epithelial cells | TGF-β1 treated | 6 | 8 | TLR1 | 1.22 | SCN11A | 0.93 | GSE6653 |
|  |  |  | vs. |  |  | MTSS1 | 1.19 | APBB2 | 0.91 |  |
|  |  |  | control |  |  | LPAR1 | 1.18 | HBEGF | 0.85 |  |
|  |  |  |  |  |  | CD47 | 1.14 | ABTB2 | 0.84 |  |
|  |  |  |  |  |  | EZH1 | 1.08 | PML | 0.84 |  |
|  |  |  |  |  |  | PON1 | 1.06 | Pvr | 0.79 |  |
|  |  |  |  |  |  |  |  | ID1 | 0.73 |  |
|  |  |  |  |  |  |  |  | GJA1 | 0.63 |  |

**Supplemental Table 11. By analyzing the microarray data from cytokine gene knock-out (KO) cells, or cytokine-treated cells, we found that LIUS-upregulated innatomic genes in mouse preosteoblast cells can be modulated slightly by a set of cytokines.** LIUS-upregulated innatomic genes are downregulated more than upregulated in proinflammatory cytokines TNF-KO (0 gene vs. 1), IL-6 KO (absence vs. 1 gene), and anti-inflammatory cytokine TGFb-treated lung carcinoma cells (3 genes vs. 6 genes) and ovarian epithelial cells (3 genes vs. 8). These results suggest that LIUS treatment of mouse preosteoblast cells slightly promote the innatomic gene expressions by enhancing proinflammatory cytokine pathways and inhibiting anti-inflammatory cytokine pathways.

| Array P |  |  |  |  |  |  |  |  |  |  |
| --- | --- | --- | --- | --- | --- | --- | --- | --- | --- | --- |
| Cytokine | Species | Tissue/cell type | comparison | number | | Up-regulated gene | Fold change | Down-regulated gene | Fold change | GeoID |
|  |  |  |  | up | down |  |  |  |  |  |
| TNF | mouse | gastric tumors | TNF KO gastric tumor | 0 | 1 |  |  | RGS3 | 0.24 | GSE43145 |
|  |  |  | vs. |  |  |  |  |  |  |  |
|  |  |  | gastric tumor |  |  |  |  |  |  |  |
| IFN1A | mouse | intestinal epithelium | IFNAR1 KO intestinal epithelium | 1 | 1 | LMCD1 | 1.23 | DBP | 0.66 | GSE76512 |
|  |  |  | vs. |  |  |  |  |  |  |  |
|  |  |  | Intestinal epithelium |  |  |  |  |  |  |  |
| IL-6 | mouse | hypothalamus | IL-6 KO mice |  | 1 |  |  | RGS3 | 0.92 | GSE34673 |
|  |  |  | vs. wt mice |  |  |  |  |  |  |  |
| IL-1β | mouse | spinal cord | IL-1b-KO | 1 | 1 | CDK5R1 | 1.08 | MMP9 | 0.80 | GSE70302 |
|  |  |  | vs. wt mice |  |  |  |  |  |  |  |
| IL-10 | mouse | lung | IL-10 KNOCK OUT | 3 | 3 | RGS2 | 1.36 | SRF | 0.87 | GSE25095 |
|  |  |  | vs. |  |  | MMP9 | 1.58 | ADAMTS1 | 0.62 |  |
|  |  |  | wt mice |  |  | RGS3 | 1.13 | NR4A1 | 0.79 |  |
| IL-35 | mouse | T cell | IL35_treated | N/A |  |  |  |  |  | GSE24210 |
|  |  |  | vs. control |  |  |  |  |  |  |  |
| TGF-β | human | A549 lung adenocarcinoma cell line | TGF-β1 treated | 3 | 6 | TFPI | 1.22 | EGR3 | 0.90 | GSE17708 |
|  |  |  | vs. |  |  | DBP | 1.18 | IGFBP4 | 0.85 |  |
|  |  |  | control |  |  | MYC | 1.14 | Tpm1 | 0.70 |  |
|  |  |  |  |  |  |  |  | LMCD1 | 0.64 |  |
|  |  |  |  |  |  |  |  | THBS1 | 0.58 |  |
|  |  |  |  |  |  |  |  | SERPINE1 | 0.38 |  |
| TGF-β | human | immortalized ovarian surface epithelial cells | TGF-β1 treated | 3 | 8 | RGS2 | 1.62 | VEGFC | 0.73 | GSE6653 |
|  |  |  | vs. |  |  | IGFBP4 | 1.60 | RGS3 | 0.72 |  |
|  |  |  | control |  |  | CH25H | 1.30 | SRF | 0.70 |  |
|  |  |  |  |  |  |  |  | SERPINE1 | 0.63 |  |
|  |  |  |  |  |  |  |  | CDK5R1 | 0.62 |  |
|  |  |  |  |  |  |  |  | THBS1 | 0.54 |  |
|  |  |  |  |  |  |  |  | Tpm1 | 0.44 |  |
|  |  |  |  |  |  |  |  | LMCD1 | 0.11 |  |

**Supplemental Table 12. By analyzing the microarray data from cytokine gene knock-out (KO) cells, or cytokine-treated cells, we found that LIUS-downregulated innatomic genes in mouse preosteoblast cells can be modulated slightly by a set of cytokines.** LIUS-upregulated innatomic genes are upregulated more than downregulated in proinflammatory cytokine TNF-KO cells (3 genes vs. 1 gene). These results suggest that LIUS treatment of mouse preosteoblast cells slightly promote the innatomic gene expressions by enhancing proinflammatory cytokine pathways and inhibiting anti-inflammatory cytokine pathways.

| Array P down | | | | | | | | | | |
| --- | --- | --- | --- | --- | --- | --- | --- | --- | --- | --- |
| Cytokine | Species | Tissue/cell type | comparison | number | | Up-regulated gene | Fold change | Down-regulated gene | Fold change | GeoID |
|  |  |  |  | up | down |  |  |  |  |  |
| TNF | mouse | gastric tumors | TNF KO gastric tumor | 3 | 1 | KRT14 | 6.57 | HOMER2 | 0.71 | GSE43145 |
|  |  |  | vs. |  |  | HMMR | 1.80 |  |  |  |
|  |  |  | gastric tumor |  |  | FABP4 | 1.72 |  |  |  |
| IFN1A | mouse | intestinal epithelium | IFNAR1 KO intestinal epithelium | 1 | 1 | BLNK | 1.23 | ID1 | 0.74 | GSE76512 |
|  |  |  | vs. |  |  |  |  |  |  |  |
|  |  |  | Intestinal epithelium |  |  |  |  |  |  |  |
| IL-6 | mouse | hypothalamus | IL-6 KO mice | 2 | 2 | ID2 | 1.16 | CHST1 | 0.94 | GSE34673 |
|  |  |  | vs. wt mice |  |  | Tmeff2 | 1.09 | LRP1 | 0.91 |  |
| IL-1β | mouse | spinal cord | IL-1b-KO | 1 | 2 | KLF10 | 1.12 | LTBP2 | 0.87 | GSE70302 |
|  |  |  | vs. wt mice |  |  |  |  | EDN1 | 0.82 |  |
| IL-10 | mouse | lung | IL-10 KNOCK OUT | 0 | 1 |  |  | ID2 | 1.28 | GSE25095 |
|  |  |  | vs. |  |  |  |  |  |  |  |
|  |  |  | wt mice |  |  |  |  |  |  |  |
| IL-35 | mouse | T cell | IL35_treated | 0 | 1 |  |  | HMMR | 1.23 | GSE24210 |
|  |  |  | vs. control |  |  |  |  |  |  |  |
| TGF-β | human | A549 lung adenocarcinoma cell line | TGF-β1 treated | 2 | 3 | ID1 | 3.14 | KLF10 | 0.72 | GSE17708 |
|  |  |  | vs. |  |  | ID2 | 1.75 | LTBP2 | 0.53 |  |
|  |  |  | control |  |  |  |  | EDN1 | 0.24 |  |
| TGF-β | human | immortalized ovarian surface epithelial cells | TGF-β1 treated | 0 | 4 |  |  | CHST1 | 0.93 | GSE6653 |
|  |  |  | vs. |  |  |  |  | KLF10 | 0.75 |  |
|  |  |  | control |  |  |  |  | ID1 | 0.73 |  |
|  |  |  |  |  |  |  |  | ID3 | 0.66 |  |

**Supplemental Table 13. By analyzing the microarray data from cytokine gene knock-out (KO) cells, or cytokine-treated cells, we found that LIUS-upregulated innatomic genes in rat bone marrow cells can be modulated by a set of cytokines.** LIUS-upregulated innatomic genes are upregulated more than downregulated in proinflammatory cytokines interleukin-6 (IL-6) KO cells (15 genes vs. 9), and IL-1b KO cells (6 genes vs 2). In addition, LIUS-upregulated innatomic genes are downregulated more than upregulated in anti-inflammatory cytokine transforming growth factor-b (TGF-b)-treated cells (10 genes vs. 34). These results suggest that LIUS treatment of bone marrow cells suppresses the innatomic gene expressions and inflammation by inhibiting proinflammatory cytokine pathways and enhancing anti-inflammatory cytokine pathways.

| LIUS-upregulated innate immunome genes in bone marrow cells |  |  |  |  |  |  |  |  |  |  |
| --- | --- | --- | --- | --- | --- | --- | --- | --- | --- | --- |
|  | Species | Tissue/cell type | comparison | number |  | Up-regulated gene | Fold change | Down-regulated gene | Fold change | GEO ID |
|  |  |  |  | up | down |  |  |  |  |  |
| TNF | mouse | gastric tumors | TNF KO gastric tumor | 7 (6.5%) | 8 (4.4%) | RASSF1 | 11.46 | MMP14 | 0.72 | GSE43145 |
|  |  |  | vs. |  |  | CKB | 2.84 | HOMER2 | 0.71 |  |
|  |  |  | gastric tumor |  |  | SCARF2 | 2.45 | ZBTB10 | 0.62 |  |
|  |  |  |  |  |  | COPZ2 | 1.73 | BHMT | 0.34 |  |
|  |  |  |  |  |  | UBA1 | 1.57 | DYRK2 | 0.28 |  |
|  |  |  |  |  |  | ELL2 | 1.49 | CCK | 0.26 |  |
|  |  |  |  |  |  | POLR3F | 1.25 | RGS3 | 0.24 |  |
|  |  |  |  |  |  |  |  | METRNL | 0.21 |  |
| IFN1A | mouse | intestinal epithelium | IFNAR1 KO intestinal epithelium | 5 (4.6%) | 11 (6.0%) | MFGE8 | 1.50 | UBA1 | 0.84 | GSE76512 |
|  |  |  | vs. |  |  | MMP14 | 1.44 | METRNL | 0.82 |  |
|  |  |  | Intestinal epithelium |  |  | PLA2G5 | 1.24 | LMAN1 | 0.82 |  |
|  |  |  |  |  |  | BLNK | 1.23 | TIMP1 | 0.81 |  |
|  |  |  |  |  |  | ACSL1 | 1.22 | GADD45B | 0.80 |  |
|  |  |  |  |  |  |  |  | EHD1 | 0.79 |  |
|  |  |  |  |  |  |  |  | CCNG2 | 0.78 |  |
|  |  |  |  |  |  |  |  | COQ10B | 0.77 |  |
|  |  |  |  |  |  |  |  | BCAR1 | 0.76 |  |
|  |  |  |  |  |  |  |  | CD3E | 0.70 |  |
|  |  |  |  |  |  |  |  | EXOC3L4 | 0.58 |  |
| IL-6 | mouse | hypothalamus | IL-6 KO mice | 15 (13.9%) | 9 (4.9%) | ID2 | 1.16 | GAS2 | 0.95 | GSE34673 |
|  |  |  | vs. |  |  | INSIG2 | 1.14 | CHAC1 | 0.93 |  |
|  |  |  | wt mice |  |  | TRPS1 | 1.14 | EXOC3L4 | 0.93 |  |
|  |  |  |  |  |  | COQ10B | 1.13 | RGS3 | 0.92 |  |
|  |  |  |  |  |  | SLC38A2 | 1.12 | PARVA | 0.92 |  |
|  |  |  |  |  |  | PPFIBP1 | 1.11 | BCAR1 | 0.92 |  |
|  |  |  |  |  |  | TBPL1 | 1.09 | ARHGEF1 | 0.89 |  |
|  |  |  |  |  |  | RNF19A | 1.09 | ILK | 0.86 |  |
|  |  |  |  |  |  | POLR3F | 1.09 | IER5 | 0.86 |  |
|  |  |  |  |  |  | CCNG2 | 1.08 |  |  |  |
|  |  |  |  |  |  | SLC4A7 | 1.08 |  |  |  |
|  |  |  |  |  |  | SSR1 | 1.08 |  |  |  |
|  |  |  |  |  |  | SPAG9 | 1.07 |  |  |  |
|  |  |  |  |  |  | CTNNB1 | 1.06 |  |  |  |
|  |  |  |  |  |  | GLB1 | 1.06 |  |  |  |
| IL-1β | mouse | spinal cord | IL-1b KO | 6 (5.6%) | 2 (1.1%) | GJA1 | 1.10 | CCNG2 | 0.92 | GSE70302 |
|  |  |  | vs. |  |  | SKIL | 1.08 | TIMP1 | 0.81 |  |
|  |  |  | wt mice |  |  | NEDD9 | 1.08 |  |  |  |
|  |  |  |  |  |  | FYN | 1.07 |  |  |  |
|  |  |  |  |  |  | CKB | 1.06 |  |  |  |
|  |  |  |  |  |  | BCAR1 | 1.05 |  |  |  |
| IL-10 | mouse | lung | IL-10 KO | 10 (9.3%) | 6 (3.3%) | TXNRD1 | 1.36 | ITPR1 | 0.89 | GSE25095 |
|  |  |  | vs. |  |  | IER5 | 1.35 | INSIG2 | 0.87 |  |
|  |  |  | wt mice |  |  | CSF1 | 1.33 | FUBP1 | 0.76 |  |
|  |  |  |  |  |  | ID2 | 1.28 | PCF11 | 0.72 |  |
|  |  |  |  |  |  | PFKFB3 | 1.21 | PLOD2 | 0.64 |  |
|  |  |  |  |  |  | EHD1 | 1.18 | PER1 | 0.51 |  |
|  |  |  |  |  |  | MXD1 | 1.15 |  |  |  |
|  |  |  |  |  |  | MAP3K14 | 1.15 |  |  |  |
|  |  |  |  |  |  | RGS3 | 1.13 |  |  |  |
|  |  |  |  |  |  | NFAT5 | 1.12 |  |  |  |
| IL-35 | mouse | T cell | IL35 treated | 1 (0.9%) | 3 (1.6%) | GAS2 | 1.46 | SOS1 | 0.73 | GSE24210 |
|  |  |  | vs. control |  |  |  |  | BCAR1 | 0.64 |  |
|  |  |  |  |  |  |  |  | PPP1R10 | 0.76 |  |

(Continued…)

| cytokine | Species | Tissue/cell type | comparison | number | | Up-regulated gene | Fold change | Down-regulated gene | Fold change | GEO ID |
| --- | --- | --- | --- | --- | --- | --- | --- | --- | --- | --- |
|  |  |  |  | up | down |  |  |  |  |  |
| TGF-β | human | A549 lung adenocarcinoma cell line | TGF-β1-treated | 10 (9.3%) | 34 (18.7%) | PHLDA1 | 2.62 | PLOD2 | 0.86 | GSE17708 |
|  |  |  | vs. |  |  | ID2 | 1.75 | GSPT1 | 0.85 |  |
|  |  |  | control |  |  | PLK2 | 1.72 | TJP1 | 0.82 |  |
|  |  |  |  |  |  | FYN | 1.62 | ARHGEF1 | 0.81 |  |
|  |  |  |  |  |  | CSF1 | 1.59 | PCF11 | 0.80 |  |
|  |  |  |  |  |  | DDR2 | 1.38 | RNF19A | 0.80 |  |
|  |  |  |  |  |  | TRPS1 | 1.32 | ELL2 | 0.79 |  |
|  |  |  |  |  |  | MDM2 | 1.28 | SDC2 | 0.78 |  |
|  |  |  |  |  |  | PHF19 | 1.26 | MMP14 | 0.77 |  |
|  |  |  |  |  |  | FKBP1B | 1.24 | ACSL1 | 0.76 |  |
|  |  |  |  |  |  | MFGE8 | 1.22 | SOS1 | 0.75 |  |
|  |  |  |  |  |  | DLX3 | 1.19 | IRGQ | 0.75 |  |
|  |  |  |  |  |  |  |  | FUBP1 | 0.75 |  |
|  |  |  |  |  |  |  |  | GJA1 | 0.75 |  |
|  |  |  |  |  |  |  |  | PTK2 | 0.74 |  |
|  |  |  |  |  |  |  |  | PPFIBP1 | 0.73 |  |
|  |  |  |  |  |  |  |  | DYRK2 | 0.73 |  |
|  |  |  |  |  |  |  |  | RGS3 | 0.72 |  |
|  |  |  |  |  |  |  |  | PPP1R10 | 0.70 |  |
|  |  |  |  |  |  |  |  | SMAD4 | 0.69 |  |
|  |  |  |  |  |  |  |  | SLC4A7 | 0.68 |  |
|  |  |  |  |  |  |  |  | HERPUD1 | 0.67 |  |
|  |  |  |  |  |  |  |  | LRP4 | 0.66 |  |
|  |  |  |  |  |  |  |  | ABCA1 | 0.61 |  |
|  |  |  |  |  |  |  |  | GADD45B | 0.57 |  |
|  |  |  |  |  |  |  |  | MXD1 | 0.55 |  |
|  |  |  |  |  |  |  |  | THBS1 | 0.54 |  |
|  |  |  |  |  |  |  |  | NFAT5 | 0.53 |  |
|  |  |  |  |  |  |  |  | CTNNB1 | 0.50 |  |
|  |  |  |  |  |  |  |  | ITPR1 | 0.45 |  |
|  |  |  |  |  |  |  |  | ENC1 | 0.45 |  |
|  |  |  |  |  |  |  |  | Tpm1 | 0.44 |  |
|  |  |  |  |  |  |  |  | SKIL | 0.32 |  |
|  |  |  |  |  |  |  |  | NEDD9 | 0.23 |  |
| TGF-β | human | immortalized ovarian surface epithelial cells | TGF-β1-treated | 7 (6.5%) | 27 (14.8%) | BCL6 | 1.25 | COQ10B | 0.96 | GSE6653 |
|  |  |  | vs. |  |  | CSF1 | 1.23 | HERPUD1 | 0.95 |  |
|  |  |  | control |  |  | GMCL1 | 1.18 | TJP1 | 0.93 |  |
|  |  |  |  |  |  | NFAT5 | 1.17 | SMAD4 | 0.92 |  |
|  |  |  |  |  |  | TXNRD1 | 1.15 | IRGQ | 0.92 |  |
|  |  |  |  |  |  | MDM2 | 1.09 | PTK2 | 0.92 |  |
|  |  |  |  |  |  | RNF19A | 1.07 | EHD1 | 0.91 |  |
|  |  |  |  |  |  |  |  | PCDH7 | 0.91 |  |
|  |  |  |  |  |  |  |  | PARVA | 0.91 |  |
|  |  |  |  |  |  |  |  | IRS1 | 0.88 |  |
|  |  |  |  |  |  |  |  | ZBTB10 | 0.87 |  |
|  |  |  |  |  |  |  |  | SLC4A7 | 0.86 |  |
|  |  |  |  |  |  |  |  | DYRK2 | 0.86 |  |
|  |  |  |  |  |  |  |  | DDR2 | 0.84 |  |
|  |  |  |  |  |  |  |  | CTNNB1 | 0.83 |  |
|  |  |  |  |  |  |  |  | PLOD2 | 0.82 |  |
|  |  |  |  |  |  |  |  | NEDD9 | 0.82 |  |
|  |  |  |  |  |  |  |  | ACSL1 | 0.81 |  |
|  |  |  |  |  |  |  |  | METRNL | 0.76 |  |
|  |  |  |  |  |  |  |  | PFKFB3 | 0.72 |  |
|  |  |  |  |  |  |  |  | Tpm1 | 0.70 |  |
|  |  |  |  |  |  |  |  | BMP2 | 0.69 |  |
|  |  |  |  |  |  |  |  | ID3 | 0.66 |  |
|  |  |  |  |  |  |  |  | GADD45B | 0.65 |  |
|  |  |  |  |  |  |  |  | SKIL | 0.64 |  |
|  |  |  |  |  |  |  |  | GJA1 | 0.63 |  |
|  |  |  |  |  |  |  |  | THBS1 | 0.58 |  |

**Supplemental Table 14. By analyzing the microarray data from cytokine gene knock-out (KO) cells, or cytokine-treated cells, we found that LIUS-downregulated innatomic genes in rat bone marrow cells can be modulated by a set of cytokines.** LIUS-downregulated innatomic genes are upregulated more than downregulated in proinflammatory cytokines TNF KO cells (14 genes vs. 10), and IL-6 KO cells (17 genes vs. 9). In addition, LIUS-downregulated innatomic genes are upregulated more than downregulated in anti-inflammatory cytokine Il-35 treated cells (5 genes vs. 2). Moreover, LIUS-downregulated innatomic genes in bone marrow cells are significantly upregulated in TGFb-treated lung carcinoma cells (39 genes vs. 12). These results suggest that LIUS treatment of bone marrow cells suppresses the innatomic gene expressions and inflammation by inhibiting proinflammatory cytokine pathways and enhancing anti-inflammatory cytokine pathways.

| LIUS-downregulated innate immunome genes in bone marrow cells | | | | | | | | | | |
| --- | --- | --- | --- | --- | --- | --- | --- | --- | --- | --- |
| Cytokine | Species | Tissue/cell type | comparison | number |  | Up-regulated gene | Fold change | Down-regulated gene | Fold change | GeoID |
|  |  |  |  | up | down |  |  |  |  |  |
| TNF | mouse | gastric tumors | TNF KO gastric tumor | 14 | 10 | CCDC39 | 10.42 | TRADD | 0.68 | GSE43145 |
|  |  |  | vs. |  |  | GBP2 | 7.65 | ITGAM | 0.65 |  |
|  |  |  | gastric tumor |  |  | PSMB9 | 4.04 | PLSCR1 | 0.62 |  |
|  |  |  |  |  |  | RSAD2 | 2.72 | EDNRB | 0.56 |  |
|  |  |  |  |  |  | DCBLD2 | 2.61 | CDK6 | 0.56 |  |
|  |  |  |  |  |  | TCF19 | 2.51 | SLC30A1 | 0.56 |  |
|  |  |  |  |  |  | SEC14L1 | 1.93 | TLR2 | 0.46 |  |
|  |  |  |  |  |  | SYK | 1.91 | MAP2K7 | 0.44 |  |
|  |  |  |  |  |  | MYT1 | 1.85 | DBNDD1 | 0.24 |  |
|  |  |  |  |  |  | RNF213 | 1.58 | CFD | 0.09 |  |
|  |  |  |  |  |  | MAP3K3 | 1.47 |  |  |  |
|  |  |  |  |  |  | EIF2AK2 | 1.41 |  |  |  |
|  |  |  |  |  |  | WARS | 1.30 |  |  |  |
|  |  |  |  |  |  | NMI | 1.28 |  |  |  |
| IFN1A | mouse | intestinal epithelium | IFNAR1 KO intestinal epithelium | 9 | 12 | IFITM2 | 1.48 | DRAM2 | 0.81 | GSE76512 |
|  |  |  | vs. |  |  | WFDC2 | 1.47 | WAS | 0.81 |  |
|  |  |  | Intestinal epithelium |  |  | NME4 | 1.38 | PTPN6 | 0.79 |  |
|  |  |  |  |  |  | MAPK9 | 1.32 | EIF2AK2 | 0.78 |  |
|  |  |  |  |  |  | TGIF1 | 1.28 | APOBEC1 | 0.71 |  |
|  |  |  |  |  |  | MTSS1 | 1.25 | NR1H3 | 0.69 |  |
|  |  |  |  |  |  | TIRAP | 1.22 | CASP4 | 0.69 |  |
|  |  |  |  |  |  | RFFL | 1.21 | PSMB9 | 0.60 |  |
|  |  |  |  |  |  | DUSP4 | 1.20 | IL18 | 0.60 |  |
|  |  |  |  |  |  |  |  | GBP2 | 0.53 |  |
|  |  |  |  |  |  |  |  | Samd9l | 0.51 |  |
|  |  |  |  |  |  |  |  | C3 | 0.26 |  |
| IL-6 | mouse | hypothalamus | IL-6 KO mice | 17 | 9 | CREG1 | 1.15 | CHST1 | 0.94 | GSE34673 |
|  |  |  | vs. |  |  | DRAM2 | 1.14 | PTPN6 | 0.93 |  |
|  |  |  | wt mice |  |  | UCHL3 | 1.14 | MAP2K7 | 0.93 |  |
|  |  |  |  |  |  | CD47 | 1.12 | ADCY3 | 0.93 |  |
|  |  |  |  |  |  | GCA | 1.11 | SAMSN1 | 0.92 |  |
|  |  |  |  |  |  | MDP1 | 1.11 | EGR1 | 0.90 |  |
|  |  |  |  |  |  | EPS15 | 1.11 | UCP2 | 0.85 |  |
|  |  |  |  |  |  | ITGA6 | 1.10 | IFITM2 | 0.84 |  |
|  |  |  |  |  |  | TROVE2 | 1.10 | PTK2B | 0.83 |  |
|  |  |  |  |  |  | RRM2 | 1.09 |  |  |  |
|  |  |  |  |  |  | EPRS | 1.08 |  |  |  |
|  |  |  |  |  |  | CCND1 | 1.07 |  |  |  |
|  |  |  |  |  |  | MAPK9 | 1.07 |  |  |  |
|  |  |  |  |  |  | SLC30A1 | 1.07 |  |  |  |
|  |  |  |  |  |  | VPS29 | 1.06 |  |  |  |
|  |  |  |  |  |  | NLRP3 | 1.06 |  |  |  |
|  |  |  |  |  |  | DUT | 1.04 |  |  |  |
| IL-1β | mouse | spinal cord | IL-1b-KO | 8 | 10 | CD83 | 1.13 | TIRAP | 0.91 | GSE70302 |
|  |  |  | vs. |  |  | ARC | 1.11 | TGIF1 | 0.91 |  |
|  |  |  | wt mice |  |  | RGS1 | 1.10 | SAT1 | 0.90 |  |
|  |  |  |  |  |  | DBNDD1 | 1.08 | TLR6 | 0.89 |  |
|  |  |  |  |  |  | UCHL3 | 1.08 | LYN | 0.89 |  |
|  |  |  |  |  |  | CDC42EP2 | 1.07 | AMPD3 | 0.88 |  |
|  |  |  |  |  |  | VPS29 | 1.06 | ELOVL3 | 0.86 |  |
|  |  |  |  |  |  | RFFL | 1.06 | PLSCR1 | 0.85 |  |
|  |  |  |  |  |  |  |  | ATF3 | 0.85 |  |
|  |  |  |  |  |  |  |  | ITGAM | 0.81 |  |
| IL-10 | mouse | lung | IL-10 KNOCK OUT | 10 | 6 | IL1B | 1.54 | IL10RA | 0.91 | GSE25095 |
|  |  |  | vs. |  |  | CCR1 | 1.46 | MAP2K7 | 0.91 |  |
|  |  |  | wt mice |  |  | MSC | 1.31 | MTSS1 | 0.87 |  |
|  |  |  |  |  |  | BID | 1.26 | EPRS | 0.86 |  |
|  |  |  |  |  |  | AXL | 1.20 | DRAM2 | 0.86 |  |
|  |  |  |  |  |  | CREG1 | 1.18 | ITPKB | 0.83 |  |
|  |  |  |  |  |  | SLC30A1 | 1.17 | SH3BP5 | 0.8050383 |  |
|  |  |  |  |  |  | ITGAM | 1.16 | MOSPD2 | 0.7812218 |  |
|  |  |  |  |  |  | NME4 | 1.15 | CDH5 | 0.708765 |  |
|  |  |  |  |  |  | TLR6 | 1.15 |  |  |  |
|  |  |  |  |  |  | NR1H3 | 1.11 |  |  |  |

(Continued…)

| IL-35 | mouse | T cell | IL35_treated | 5 | 2 | MAF | 1.38 | RSAD2 | 0.80 | GSE24210 |
| --- | --- | --- | --- | --- | --- | --- | --- | --- | --- | --- |
|  |  |  | vs. control |  |  | CDH5 | 1.35 | TROVE2 | 0.69 |  |
|  |  |  |  |  |  | TIRAP | 1.32 |  |  |  |
|  |  |  |  |  |  | PLSCR1 | 1.30 |  |  |  |
|  |  |  |  |  |  | APOB | 1.29 |  |  |  |
| TGF-β | human | A549 lung adenocarcinoma cell line | TGF-β1 treated | 39 | 12 | MAFB | 4.14 | BCL10 | 0.87 | GSE17708 |
|  |  |  | vs. |  |  | PPP1R3C | 1.96 | CCND1 | 0.79 |  |
|  |  |  | control |  |  | EGR1 | 1.92 | CSF1R | 0.74 |  |
|  |  |  |  |  |  | RSAD2 | 1.75 | TLR2 | 0.74 |  |
|  |  |  |  |  |  | Samd9l | 1.69 | FCER1G | 0.74 |  |
|  |  |  |  |  |  | TMEM140 | 1.63 | MAP3K2 | 0.60 |  |
|  |  |  |  |  |  | DUSP4 | 1.62 | ALCAM | 0.58 |  |
|  |  |  |  |  |  | IGFBP4 | 1.60 | INHBA | 0.58 |  |
|  |  |  |  |  |  | DUSP6 | 1.59 | MSC | 0.53 |  |
|  |  |  |  |  |  | DCBLD2 | 1.56 | PELI2 | 0.52 |  |
|  |  |  |  |  |  | RNF213 | 1.50 | HMOX1 | 0.51 |  |
|  |  |  |  |  |  | CASP1 | 1.48 | CDK6 | 0.29 |  |
|  |  |  |  |  |  | EIF2AK2 | 1.46 |  |  |  |
|  |  |  |  |  |  | NPR3 | 1.45 |  |  |  |
|  |  |  |  |  |  | JUN | 1.40 |  |  |  |
|  |  |  |  |  |  | THBD | 1.38 |  |  |  |
|  |  |  |  |  |  | ARHGAP25 | 1.37 |  |  |  |
|  |  |  |  |  |  | AXL | 1.35 |  |  |  |
|  |  |  |  |  |  | LMO2 | 1.34 |  |  |  |
|  |  |  |  |  |  | NLRP3 | 1.34 |  |  |  |
|  |  |  |  |  |  | MAP2K7 | 1.32 |  |  |  |
|  |  |  |  |  |  | ITGA6 | 1.31 |  |  |  |
|  |  |  |  |  |  | GCA | 1.30 |  |  |  |
|  |  |  |  |  |  | NR1H3 | 1.30 |  |  |  |
|  |  |  |  |  |  | CH25H | 1.30 |  |  |  |
|  |  |  |  |  |  | TGIF1 | 1.28 |  |  |  |
|  |  |  |  |  |  | VAT1 | 1.27 |  |  |  |
|  |  |  |  |  |  | RNF166 | 1.27 |  |  |  |
|  |  |  |  |  |  | CD47 | 1.27 |  |  |  |
|  |  |  |  |  |  | MTSS1 | 1.26 |  |  |  |
|  |  |  |  |  |  | SCAP | 1.26 |  |  |  |
|  |  |  |  |  |  | CD14 | 1.26 |  |  |  |
|  |  |  |  |  |  | UCP2 | 1.25 |  |  |  |
|  |  |  |  |  |  | IRF4 | 1.24 |  |  |  |
|  |  |  |  |  |  | NME4 | 1.23 |  |  |  |
|  |  |  |  |  |  | WFDC2 | 1.23 |  |  |  |
|  |  |  |  |  |  | CD163 | 1.19 |  |  |  |
|  |  |  |  |  |  | RCSD1 | 1.19 |  |  |  |
|  |  |  |  |  |  | TIRAP | 1.16 |  |  |  |
| TGF-β | human | immortalized ovarian surface epithelial cells | TGF-β1 treated | 19 | 30 | CCL20 | 2.19 | DRAM2 | 0.96 | GSE6653 |
|  |  |  | vs. |  |  | HHEX | 1.41 | SET | 0.95 |  |
|  |  |  | control |  |  | NEIL3 | 1.36 | TGIF1 | 0.94 |  |
|  |  |  |  |  |  | PELI2 | 1.25 | TROVE2 | 0.93 |  |
|  |  |  |  |  |  | CDK1 | 1.23 | MSC | 0.93 |  |
|  |  |  |  |  |  | MAD2L1 | 1.22 | CHST1 | 0.93 |  |
|  |  |  |  |  |  | MTSS1 | 1.19 | SEC14L1 | 0.91 |  |
|  |  |  |  |  |  | DUSP4 | 1.17 | WAS | 0.90 |  |
|  |  |  |  |  |  | EPRS | 1.14 | BCL10 | 0.90 |  |
|  |  |  |  |  |  | ALCAM | 1.14 | RSAD2 | 0.90 |  |
|  |  |  |  |  |  | CD47 | 1.14 | MAP2K7 | 0.89 |  |
|  |  |  |  |  |  | TMEM140 | 1.14 | MAP3K2 | 0.89 |  |
|  |  |  |  |  |  | WFDC2 | 1.12 | ZMYND8 | 0.89 |  |
|  |  |  |  |  |  | Rps6ka5 | 1.10 | CCND1 | 0.87 |  |
|  |  |  |  |  |  | CASP1 | 1.08 | PLK3 | 0.87 |  |
|  |  |  |  |  |  | DUT | 1.08 | PTK2B | 0.86 |  |
|  |  |  |  |  |  | ARHGAP25 | 1.07 | SPRY2 | 0.85 |  |
|  |  |  |  |  |  | LIPA | 1.07 | IGFBP4 | 0.85 |  |
|  |  |  |  |  |  | AKAP10 | 1.06 | IRF4 | 0.84 |  |
|  |  |  |  |  |  |  |  | IL7R | 0.82 |  |
|  |  |  |  |  |  |  |  | PTPRJ | 0.82 |  |
|  |  |  |  |  |  |  |  | SLC30A1 | 0.81 |  |
|  |  |  |  |  |  |  |  | PPP1R3C | 0.80 |  |
|  |  |  |  |  |  |  |  | MAF | 0.80 |  |
|  |  |  |  |  |  |  |  | Ccl2 | 0.79 |  |
|  |  |  |  |  |  |  |  | CCL2 | 0.79 |  |
|  |  |  |  |  |  |  |  | HMOX1 | 0.74 |  |
|  |  |  |  |  |  |  |  | PLAU | 0.70 |  |
|  |  |  |  |  |  |  |  | MAFB | 0.67 |  |
|  |  |  |  |  |  |  |  | JUN | 0.64 |  |

**Supplemental Table 15A. The microarrays of two T cell co-inhibition receptors B7-H4 (VTCN1) and BTNL2 were used in this study to determine whether LIUS-modulation of innatomic genes uses the reverse signaling pathways of the T cell co-inhibition receptors (see our recent report, PMID: 30468648).**

| Gene Symbol | Main Function | Species | Cell Type | Comparison | GEO ID |
| --- | --- | --- | --- | --- | --- |
| VTCN1 | a negative T-cell regulator | mouse | CD8 T cells | AI4 CD8+T cell from Rip-B7xAI4 mice vs. AI4 CD8+T cell from AI4 mice | GSE40225 |
| BTNL2 | a negative T-cell regulator | mouse | CD4+CD25- cells | CD4 anti-CD3 B7-2 with BTNL2 overexpression vs. CD4 anti-CD3 B7-2 cell | GSE42385 |

**Supplemental Table 15B. Overexpression of co-inhibition receptor VTCN1 (B7-H4) promotes more LIUS-upregulated innatomic genes (8 genes, 10.4%) than downregulating these genes in lymphoma cells (2 genes, 5.1%). But, VTCN1 promotes more LIUS-downregulated innatomic genes (27, 14.8%) than upregulating these genes in bone marrow cells (10 genes, 9.3%).**

| Treatment | VTCN1 overexpression dataset GSE40225 | | |  |  |  |  |
| --- | --- | --- | --- | --- | --- | --- | --- |
| Primary GEO ID | GSE10212 | | |  |  |  |  |
|  | LIUS-upregulated gene (77 genes in human lymphoma cells) | |  |  | LIUS-downregulated gene (39 genes in human lymphoma cells) | | |
| Trend | Gene | P. value | Fold Change | Trend | Gene | P. value | Fold Change |
| Upregulated | MERTK | 0.05 | 2.58 | Upregulated | ITGB1 | 0.00 | 3.61 |
| 8 (10.4%) | CAPN2 | 0.01 | 2.57 | 2 (5.1%) | ARHGDIA | 0.04 | 1.54 |
|  | CD44 | 0.02 | 2.39 |  |  |  |  |
|  | S100A10 | 0.00 | 2.19 |  |  |  |  |
|  | IL2RB | 0.02 | 2.11 |  |  |  |  |
|  | DUSP4 | 0.03 | 2.00 |  |  |  |  |
|  | ELL2 | 0.04 | 1.81 |  |  |  |  |
|  | MBD2 | 0.01 | 1.70 |  |  |  |  |
|  | IL10 | 0.01 | 1.63 |  |  |  |  |
|  | FTH1 | 0.04 | 1.58 |  |  |  |  |
|  | CSF1 | 0.04 | 1.30 |  |  |  |  |
| Downregulated | ADCY2 | 0.03 | 0.71 | Downregulated | RAB33A | 0.03 | 0.76 |
| 1 (1.3%) |  |  |  | 2 (5.1%) | TLR1 | 0.00 | 0.30 |
| Primary GEO ID | GSE45487 | | |  |  |  |  |
|  | LIUS-upregulated gene (21 genes in preosteoblast cells) | |  |  | LIUS-downregulated gene (17 genes in preosteoblast cells) | | |
| Trend | Gene | P. value | Fold Change | Trend | Gene | P. value | Fold Change |
| Upregulated |  |  |  | Upregulated | ID2 | 0.00 | 8.46 |
| 0 |  |  |  | 2 (11.8%) | HMMR | 0.00 | 5.90 |
| Downregulated | IGFBP4 | 0.01 | 0.60 | Downregulated |  |  |  |
| 1 (4.8%) |  |  |  | 0 |  |  |  |
| Primary GEO ID | GSE70662 | | |  |  |  |  |
|  | LIUS-upregulated gene (108 genes in bone marrow cells) | |  |  | LIUS-downregulated gene (182 genes in bone marrow cells) | | |
| Trend | Gene | P. value | Fold Change | Trend | Gene | P. value | Fold Change |
| Upregulated | ID2 | 0.00 | 8.46 | Upregulated | PTPRJ | 0.00 | 6.15 |
| 10 (9.3%) | TRPS1 | 0.02 | 5.90 | 27 (14.8%) | MAD2L1 | 0.00 | 6.06 |
|  | ILK | 0.01 | 2.30 |  | CASP1 | 0.00 | 6.02 |
|  | ISG20 | 0.04 | 1.94 |  | Ifitm1 | 0.03 | 5.43 |
|  | ELL2 | 0.04 | 1.81 |  | CD68 | 0.00 | 4.44 |
|  | PPP1R10 | 0.01 | 1.64 |  | IFITM2 | 0.04 | 4.41 |
|  | GMCL1 | 0.01 | 1.58 |  | IFI30 | 0.00 | 4.20 |
|  | SSR1 | 0.04 | 1.48 |  | PLSCR1 | 0.00 | 4.03 |
|  | GADD45B | 0.04 | 1.47 |  | DRAM2 | 0.00 | 4.00 |
|  | CSF1 | 0.04 | 1.30 |  | IL1B | 0.03 | 3.94 |
|  |  |  |  |  | CDK1 | 0.01 | 3.66 |
|  |  |  |  |  | FCER1G | 0.02 | 3.32 |
|  |  |  |  |  | CD48 | 0.03 | 3.18 |
|  |  |  |  |  | LIPA | 0.00 | 2.97 |
|  |  |  |  |  | VPS29 | 0.02 | 2.89 |
|  |  |  |  |  | EIF2AK2 | 0.02 | 2.66 |
|  |  |  |  |  | RRM2 | 0.02 | 2.58 |
|  |  |  |  |  | CASP4 | 0.04 | 2.50 |
|  |  |  |  |  | TLR4 | 0.03 | 2.23 |
|  |  |  |  |  | DUT | 0.01 | 2.11 |
|  |  |  |  |  | DUSP4 | 0.03 | 2.00 |
|  |  |  |  |  | EPS15 | 0.05 | 1.98 |
|  |  |  |  |  | PSMB9 | 0.00 | 1.98 |
|  |  |  |  |  | IL10RA | 0.03 | 1.73 |
|  |  |  |  |  | CENPA | 0.05 | 1.64 |
|  |  |  |  |  | IL10 | 0.01 | 1.63 |
|  |  |  |  |  | UCP2 | 0.04 | 1.37 |
| Downregulated | SCARF2 | 0.04 | 0.70 | Downregulated | RFFL | 0.05 | 0.78 |
| 1 (0.9%) |  |  |  | 3 (1.6%) | RCSD1 | 0.01 | 0.63 |
|  |  |  |  |  | IGFBP4 | 0.01 | 0.60 |

**Supplemental Table 16A. Overexpression of co-inhibition receptor butyrophilin-like 2 (BTNL2) promotes more LIUS-upregulated innatomic genes than downregulating these genes.** The results showed in lymphoma cells, overexpression of BTNL2 downregulates 20.8%, more than upregulation (16.9%) of LIUS-upregulated 77 genes. In addition, BTNL2 increased 28.2%, more than downregulation (23.1%) of 39 LIUS-downregulated genes. These results suggest that BTNL2 overexpression inhibits more LIUS upregulated genes and promotes more LIUS-downregulated genes. In addition, the results showed that in preosteoblast cells, overexpression of BTNL2 downregulates 42.9%, more than upregulation (28.6%) of LIUS-upregulated 21 genes. In addition, BTNL2 increased 23.5%, more than downregulation (17.6%) of 17 LIUS-downregulated genes. These results suggest that BTNL2 overexpression inhibits more LIUS-upregulated genes and promotes more LIUS-downregulated genes. Moreover, the results showed that in bone marrow cells, overexpression of BTNL2 downregulates 32.4%, more than upregulation (23.1%) of LIUS-upregulated 108 genes. In addition, BTNL2 increased 29.1%, the same as downregulation (29.1%) of 182 LIUS-downregulated genes. These results suggest that BTNL2 overexpression inhibits more LIUS-upregulated genes and up-, down-regulates the same numbers (29.1%) of LIUS-downregulated genes.

| Treatment | BTNL2 overexpression dataset GSE43285 | | |  |  |  |  |
| --- | --- | --- | --- | --- | --- | --- | --- |
| Primary GEO ID | GSE10212 | | |  |  |  |  |
|  | LIUS-Upregulated Genes (77 genes in lymphoma cells) | |  |  | LIUS-Downregulated Genes (39 genes in lymphoma cells) | | |
| Modulation | Gene | P. value | Fold Change | Trend | Gene | P. value | Fold Change |
| Upregulated | ITGB3 | 0.00 | 4.43 | Upregulated | TLR1 | 0.00 | 5.39 |
| 13 (16.9%) | NR4A3 | 0.00 | 4.24 | 11 (28.2%) | RGS3 | 0.00 | 2.47 |
|  | CXCL10 | 0.00 | 3.91 |  | Tnik | 0.00 | 2.28 |
|  | IL1RL1 | 0.00 | 3.22 |  | TLR7 | 0.00 | 1.98 |
|  | CD40 | 0.00 | 3.16 |  | SRC | 0.00 | 1.51 |
|  | JUN | 0.00 | 2.93 |  | DUSP2 | 0.00 | 1.46 |
|  | CCL20 | 0.00 | 2.40 |  | AFF1 | 0.01 | 1.39 |
|  | FYN | 0.00 | 1.85 |  | CCNG2 | 0.04 | 1.35 |
|  | CSF1 | 0.00 | 1.78 |  | CD47 | 0.01 | 1.35 |
|  | MBD2 | 0.00 | 1.55 |  | Pvr | 0.00 | 1.35 |
|  | SGK1 | 0.00 | 1.44 |  | ITGB1 | 0.01 | 1.30 |
|  | ADCY2 | 0.01 | 1.34 |  |  |  |  |
|  | Sp100 | 0.01 | 1.32 |  |  |  |  |
| Downregulated | SERPINE1 | 0.01 | 0.79 | Downregulated | PML | 0.01 | 0.77 |
| 16 (20.8%) | FABP7 | 0.02 | 0.77 | 9 (23.1%) | ABTB2 | 0.01 | 0.76 |
|  | HMOX1 | 0.01 | 0.76 |  | ZFP36L2 | 0.01 | 0.76 |
|  | SLC2A1 | 0.01 | 0.75 |  | MTSS1 | 0.02 | 0.72 |
|  | PIM1 | 0.01 | 0.75 |  | APBB2 | 0.00 | 0.69 |
|  | F3 | 0.02 | 0.73 |  | CHAC1 | 0.01 | 0.68 |
|  | MMP14 | 0.00 | 0.67 |  | HBEGF | 0.01 | 0.68 |
|  | ELL2 | 0.00 | 0.66 |  | RAB33A | 0.00 | 0.50 |
|  | TRAF1 | 0.00 | 0.66 |  | GJA1 | 0.00 | 0.11 |
|  | SCARB1 | 0.00 | 0.65 |  |  |  |  |
|  | MFGE8 | 0.01 | 0.63 |  |  |  |  |
|  | DUSP4 | 0.00 | 0.62 |  |  |  |  |
|  | CCR1 | 0.00 | 0.55 |  |  |  |  |
|  | GPX3 | 0.00 | 0.53 |  |  |  |  |
|  | NR4A1 | 0.00 | 0.53 |  |  |  |  |
|  | KLF6 | 0.00 | 0.51 |  |  |  |  |
|  | S100A10 | 0.00 | 0.48 |  |  |  |  |
|  | C3 | 0.01 | 0.47 |  |  |  |  |
|  | IL10 | 0.00 | 0.39 |  |  |  |  |
|  | PHLDA1 | 0.00 | 0.36 |  |  |  |  |
|  | SDC4 | 0.00 | 0.26 |  |  |  |  |
|  | FOSL2 | 0.00 | 0.23 |  |  |  |  |
|  | RGS2 | 0.00 | 0.21 |  |  |  |  |
|  | GSTM5 | 0.00 | 0.16 |  |  |  |  |
|  | IER3 | 0.00 | 0.13 |  |  |  |  |
|  | SERPINF1 | 0.00 | 0.06 |  |  |  |  |
| Primary GEO ID | GSE45487 | | |  |  |  |  |
|  | LIUS-Upregulated Gene (21 genes in pre-osteoblast cells)) | |  |  | LIUS-Downregulated Gene (17 genes in pre-osteoblast cells) | | |
| Modulation | Gene | P. value | Fold Change | Trend | Gene | P. value | Fold Change |
| Upregulated | IGFBP4 | 0.00 | 2.49 | Upregulated | CDH1 | 0.00 | 28.49 |
| 6 (28.6%) | RGS3 | 0.00 | 2.47 | 4 (23.5%) | ID3 | 0.00 | 2.72 |
|  | Tpm1 | 0.02 | 1.38 |  | BLNK | 0.02 | 1.43 |
|  | CDK5R1 | 0.01 | 1.35 |  | Tmeff2 | 0.01 | 1.40 |
|  | MMP9 | 0.04 | 1.31 |  |  |  |  |
|  | DBP | 0.04 | 1.21 |  |  |  |  |
| Downregulated | SERPINE1 | 0.01 | 0.79 | Downregulated | ID2 | 0.00 | 0.65 |
| 9 (42.9%) | SRF | 0.02 | 0.76 | 3 (17.6%) | LRP1 | 0.00 | 0.62 |
|  | LMCD1 | 0.01 | 0.72 |  | IL1RN | 0.00 | 0.19 |
|  | MMP13 | 0.00 | 0.65 |  |  |  |  |
|  | TFPI | 0.00 | 0.65 |  |  |  |  |
|  | NR4A1 | 0.00 | 0.53 |  |  |  |  |
|  | MYC | 0.00 | 0.50 |  |  |  |  |
|  | PLAGL1 | 0.00 | 0.47 |  |  |  |  |
|  | RGS2 | 0.00 | 0.21 |  |  |  |  |

**Supplemental Tables 16B and 16C**. In addition, the results showed that in preosteoblast cells, overexpression of BTNL2 downregulates 42.9%, more than upregulation (28.6%) of LIUS-upregulated 21 genes. In addition, BTNL2 increased 23.5%, more than downregulation (17.6%) of 17 LIUS-downregulated genes. These results suggest that BTNL2 overexpression inhibits more LIUS-upregulated genes and promotes more LIUS-downregulated genes. Moreover, the results showed that in bone marrow cells, overexpression of BTNL2 downregulates 32.4%, more than upregulation (23.1%) of LIUS-upregulated 108 genes. In addition, BTNL2 increased 29.1%, the same as downregulation (29.1%) of 182 LIUS-downregulated genes. These results suggest that BTNL2 overexpression inhibits more LIUS-upregulated genes and up-, down-regulates the same numbers (29.1%) of LIUS-downregulated genes.

| Primary GEO ID | GSE70662 | | |  |  |  |  |
| --- | --- | --- | --- | --- | --- | --- | --- |
|  | LIUS-Upregulated Gene (108 genes in bone marrow cells) | |  |  | LIUS-Downregulated Gene (182 genes in bone marrow cells) | | |
| Modulation | Gene | P. value | Fold Change | Modulation | Gene | P. value | Fold Change |
| Downregulated | UBA1 | 0.04 | 0.84 | Downregulated | PLK3 | 0.04 | 0.84 |
| 35 (32.4%) | BCAR1 | 0.03 | 0.81 | 53 (29.1%) | MDP1 | 0.04 | 0.81 |
|  | POLR3D | 0.03 | 0.81 |  | MSC | 0.05 | 0.81 |
|  | EHD1 | 0.03 | 0.80 |  | PPCDC | 0.02 | 0.81 |
|  | GMCL1 | 0.01 | 0.77 |  | IFITM2 | 0.04 | 0.79 |
|  | OSTC | 0.02 | 0.76 |  | CCND1 | 0.01 | 0.79 |
|  | LMAN1 | 0.02 | 0.74 |  | GBP4 | 0.02 | 0.79 |
|  | HDAC5 | 0.01 | 0.74 |  | SAMSN1 | 0.01 | 0.77 |
|  | INSIG2 | 0.00 | 0.74 |  | HMOX1 | 0.01 | 0.76 |
|  | RASSF1 | 0.00 | 0.69 |  | PIM1 | 0.01 | 0.75 |
|  | PPFIBP1 | 0.00 | 0.68 |  | MTSS1 | 0.02 | 0.72 |
|  | CHAC1 | 0.01 | 0.68 |  | SAT1 | 0.00 | 0.69 |
|  | MMP14 | 0.00 | 0.67 |  | SPRY2 | 0.00 | 0.69 |
|  | ELL2 | 0.00 | 0.66 |  | EPRS | 0.00 | 0.69 |
|  | FKBP1B | 0.00 | 0.66 |  | MOSPD2 | 0.00 | 0.67 |
|  | MMP13 | 0.00 | 0.65 |  | CD83 | 0.00 | 0.66 |
|  | SLC4A7 | 0.01 | 0.65 |  | CD68 | 0.00 | 0.65 |
|  | ID2 | 0.00 | 0.65 |  | VPS29 | 0.01 | 0.62 |
|  | MFGE8 | 0.01 | 0.63 |  | DUSP4 | 0.00 | 0.62 |
|  | ZBTB10 | 0.00 | 0.63 |  | LYL1 | 0.04 | 0.61 |
|  | TBPL1 | 0.00 | 0.62 |  | MAF | 0.00 | 0.59 |
|  | NFAT5 | 0.00 | 0.62 |  | EGR1 | 0.00 | 0.58 |
|  | H1f0 | 0.00 | 0.62 |  | NPR3 | 0.00 | 0.58 |
|  | RNF19A | 0.00 | 0.61 |  | TROVE2 | 0.00 | 0.57 |
|  | GLB1 | 0.00 | 0.59 |  | VAT1 | 0.00 | 0.57 |
|  | TRPS1 | 0.00 | 0.55 |  | DUSP6 | 0.00 | 0.56 |
|  | GPX3 | 0.00 | 0.53 |  | DRAM2 | 0.00 | 0.55 |
|  | PTPN4 | 0.00 | 0.48 |  | CCR1 | 0.00 | 0.55 |
|  | RAPH1 | 0.00 | 0.48 |  | CASP4 | 0.00 | 0.54 |
|  | COPZ2 | 0.00 | 0.39 |  | AMPD3 | 0.00 | 0.53 |
|  | PHLDA1 | 0.00 | 0.36 |  | C3 | 0.01 | 0.47 |
|  | GAS2 | 0.00 | 0.29 |  | HHEX | 0.00 | 0.46 |
|  | PLK2 | 0.00 | 0.26 |  | EBI3 | 0.00 | 0.43 |
|  | GJA1 | 0.00 | 0.11 |  | IL10 | 0.00 | 0.39 |
|  | TIMP1 | 0.00 | 0.06 |  | TIRAP | 0.00 | 0.39 |
|  |  |  |  |  | Rps6ka5 | 0.00 | 0.34 |
|  |  |  |  |  | NME4 | 0.00 | 0.31 |
|  |  |  |  |  | IRF4 | 0.00 | 0.28 |
|  |  |  |  |  | IL18 | 0.00 | 0.25 |
|  |  |  |  |  | ATF3 | 0.00 | 0.22 |
|  |  |  |  |  | INHBA | 0.00 | 0.03 |

Supplemental Table 16C

| Primary GEO ID | GSE70662 | | |  |  |  |  |
| --- | --- | --- | --- | --- | --- | --- | --- |
|  | Primary Upregulated Gene (108) | |  |  | Primary Downregulated Gene (182) | | |
| Modulation | Gene | P. value | Fold Change | Modulation | Gene | P. value | Fold Change |
| Upregulated | ISG20 | 0.00 | 13.95 | Upregulated | CDH1 | 0.00 | 28.49 |
| 25 (23.1%) | MXD1 | 0.00 | 3.49 | 53 (29.1%) | Samd9l | 0.00 | 9.07 |
|  | ID3 | 0.00 | 2.72 |  | RSAD2 | 0.00 | 5.19 |
|  | RGS3 | 0.00 | 2.47 |  | SH3BP5 | 0.00 | 3.64 |
|  | METRNL | 0.00 | 1.98 |  | ITGA6 | 0.00 | 3.30 |
|  | DYRK2 | 0.00 | 1.98 |  | IL1RL1 | 0.00 | 3.22 |
|  | BCL6 | 0.00 | 1.90 |  | FCER1G | 0.00 | 3.20 |
|  | COQ10B | 0.00 | 1.87 |  | LTB | 0.00 | 2.94 |
|  | FYN | 0.00 | 1.85 |  | JUN | 0.00 | 2.93 |
|  | SKIL | 0.00 | 1.83 |  | GBP2 | 0.00 | 2.80 |
|  | CSF1 | 0.00 | 1.78 |  | TLR2 | 0.00 | 2.69 |
|  | NEDD9 | 0.00 | 1.76 |  | Ccl9 | 0.00 | 2.68 |
|  | PLOD2 | 0.00 | 1.45 |  | TMEM176B | 0.00 | 2.65 |
|  | BLNK | 0.02 | 1.43 |  | IGFBP4 | 0.00 | 2.49 |
|  | Tpm1 | 0.02 | 1.38 |  | CNR2 | 0.00 | 2.43 |
|  | ARHGEF1 | 0.00 | 1.37 |  | CCL20 | 0.00 | 2.40 |
|  | DLX3 | 0.01 | 1.37 |  | RNF213 | 0.00 | 2.33 |
|  | CCNG2 | 0.04 | 1.35 |  | ARHGAP25 | 0.00 | 2.30 |
|  | PER1 | 0.02 | 1.34 |  | IFNGR1 | 0.00 | 2.10 |
|  | ACSL1 | 0.01 | 1.33 |  | NMI | 0.00 | 2.00 |
|  | CTNNB1 | 0.01 | 1.29 |  | TLR6 | 0.00 | 2.00 |
|  | SMAD4 | 0.04 | 1.28 |  | TLR7 | 0.00 | 1.98 |
|  | GADD45B | 0.04 | 1.26 |  | LYN | 0.00 | 1.84 |
|  | SLC38A2 | 0.05 | 1.21 |  | ICAM2 | 0.00 | 1.77 |
|  | TXNRD1 | 0.04 | 1.20 |  | DOCK8 | 0.00 | 1.76 |
|  |  |  |  |  | ARC | 0.00 | 1.70 |
|  |  |  |  |  | TMEM140 | 0.01 | 1.62 |
|  |  |  |  |  | ALCAM | 0.00 | 1.61 |
|  |  |  |  |  | Slfn2 | 0.00 | 1.56 |
|  |  |  |  |  | IL10RA | 0.00 | 1.54 |
|  |  |  |  |  | PTPRJ | 0.00 | 1.53 |
|  |  |  |  |  | LIPA | 0.01 | 1.51 |
|  |  |  |  |  | IFI30 | 0.00 | 1.49 |
|  |  |  |  |  | UCHL3 | 0.00 | 1.49 |
|  |  |  |  |  | TGIF1 | 0.00 | 1.48 |
|  |  |  |  |  | ITPKB | 0.00 | 1.46 |
|  |  |  |  |  | RCSD1 | 0.00 | 1.42 |
|  |  |  |  |  | PTK2B | 0.00 | 1.41 |
|  |  |  |  |  | APOBR | 0.01 | 1.40 |
|  |  |  |  |  | MAP3K2 | 0.02 | 1.38 |
|  |  |  |  |  | ZMYND8 | 0.01 | 1.37 |
|  |  |  |  |  | CD47 | 0.01 | 1.35 |
|  |  |  |  |  | EIF2AK2 | 0.00 | 1.35 |
|  |  |  |  |  | NEIL3 | 0.01 | 1.34 |
|  |  |  |  |  | PSMB9 | 0.01 | 1.34 |
|  |  |  |  |  | TRADD | 0.01 | 1.31 |
|  |  |  |  |  | CKLF | 0.01 | 1.30 |
|  |  |  |  |  | AKAP10 | 0.02 | 1.26 |
|  |  |  |  |  | SLC30A1 | 0.02 | 1.25 |
|  |  |  |  |  | CREG1 | 0.02 | 1.25 |
|  |  |  |  |  | MAPK9 | 0.03 | 1.24 |
|  |  |  |  |  | TCF19 | 0.03 | 1.23 |
|  |  |  |  |  | BCL10 | 0.03 | 1.21 |

**Supplemental Table 17A. LIUS-upregulated genes in bone marrow cells are classified into four groups, reactive oxygen species (ROS)-dependent; ROS-suppressed; ROS-dependent/suppressed; and ROS-independent.** 44 out of total 108 (40.74%) LIUS-upregulated genes in bone marrow cells are identified as reactive oxygen species (ROS)-dependent upregulations, which were upregulated in anti-oxidant transcription factor **Nuclear factor erythroid 2-related factor 2** (Nrf2)-knocked out dataset (GSE7810) or/and downregulated in pro-oxidant nicotinamide adenine dinucleotide phosphate (NADPH) oxidase 2 (NOX2)-knocked out dataset (GSE35446). Meanwhile, 30 of 108 genes (27.78%) are identified as ROS-suppressed upregulations. Four genes are shared, including PLCG1, CCK, COQ10B and ARHGEF1.

| Up regulate genes in Bone marrow cells | | | | | | | | | | | |  |
| --- | --- | --- | --- | --- | --- | --- | --- | --- | --- | --- | --- | --- |
| ROS promoter genes | p value | Fold Change | dataset | tissue | KO/  Inhibit Target | ROS inhibitor genes | p value | Fold Change | dataset | tissue | KO/  Inhibit Target | Dual |
| TRPS1 | 0.00 | 2.94 | GSE7810 | liver | Nrf2 | CKB | 0.05 | 0.76 | GSE7810 | liver | Nrf2 | PLCG1 |
| BLNK | 0.00 | 2.52 | GSE7810 | liver | Nrf2 | CHAC1 | 0.03 | 0.72 | GSE7810 | liver | Nrf2 | CCK |
| ELL2 | 0.05 | 2.51 | GSE7810 | liver | Nrf2 | GSPT1 | 0.02 | 0.72 | GSE7810 | liver | Nrf2 | COQ10B |
| PPFIBP1 | 0.02 | 2.40 | GSE7810 | liver | Nrf2 | IRS1 | 0.01 | 0.68 | GSE7810 | liver | Nrf2 | ARHGEF1 |
| MXD1 | 0.00 | 2.38 | GSE7810 | liver | Nrf2 | COQ10B | 0.02 | 0.67 | GSE7810 | liver | Nrf2 |  |
| LMAN1 | 0.00 | 2.19 | GSE7810 | liver | Nrf2 | PTK2 | 0.02 | 0.66 | GSE7810 | liver | Nrf2 |  |
| RAPH1 | 0.00 | 2.11 | GSE7810 | liver | Nrf2 | TJP1 | 0.02 | 0.65 | GSE7810 | liver | Nrf2 |  |
| GPX3 | 0.00 | 1.87 | GSE7810 | liver | Nrf2 | IER5 | 0.02 | 0.62 | GSE7810 | liver | Nrf2 |  |
| NFKBIA | 0.00 | 1.79 | GSE7810 | liver | Nrf2 | ICAM1 | 0.00 | 0.60 | GSE7810 | liver | Nrf2 |  |
| ABCA1 | 0.01 | 1.79 | GSE7810 | liver | Nrf2 | GJA1 | 0.02 | 0.58 | GSE7810 | liver | Nrf2 |  |
| PDGFRL | 0.00 | 1.73 | GSE7810 | liver | Nrf2 | BCAR1 | 0.02 | 0.54 | GSE7810 | liver | Nrf2 |  |
| METRNL | 0.00 | 1.66 | GSE7810 | liver | Nrf2 | TXNRD1 | 0.00 | 0.54 | GSE7810 | liver | Nrf2 |  |
| BMP2 | 0.05 | 1.63 | GSE7810 | liver | Nrf2 | GADD45B | 0.00 | 0.52 | GSE7810 | liver | Nrf2 |  |
| HERPUD1 | 0.00 | 1.57 | GSE7810 | liver | Nrf2 | SDC2 | 0.00 | 0.46 | GSE7810 | liver | Nrf2 |  |
| MMP13 | 0.02 | 1.52 | GSE7810 | liver | Nrf2 | DLX3 | 0.00 | 0.28 | GSE7810 | liver | Nrf2 |  |
| PLCG1 | 0.01 | 1.52 | GSE7810 | liver | Nrf2 | HOMER2 | 0.00 | 0.22 | GSE7810 | liver | Nrf2 |  |
| STRN3 | 0.01 | 1.52 | GSE7810 | liver | Nrf2 | CD3E | 0.00 | 1.68 | GSE36446 | cortex | NOX2 |  |
| RASSF1 | 0.02 | 1.46 | GSE7810 | liver | Nrf2 | ARHGEF1 | 0.02 | 1.23 | GSE36446 | cortex | NOX2 |  |
| CCK | 0.03 | 1.36 | GSE7810 | liver | Nrf2 | CKB | 0.04 | 1.22 | GSE36446 | cortex | NOX2 |  |
| FYN | 0.05 | 1.34 | GSE7810 | liver | Nrf2 | ENC1 | 0.02 | 1.19 | GSE36446 | cortex | NOX2 |  |
| GAS2 | 0.03 | 1.34 | GSE7810 | liver | Nrf2 | THBS4 | 0.04 | 1.12 | GSE36446 | cortex | NOX2 |  |
| ACSL1 | 0.04 | 1.30 | GSE7810 | liver | Nrf2 | NFAT5 | 0.01 | 1.10 | GSE36446 | cortex | NOX2 |  |
| PLK2 | 0.02 | 0.92 | GSE36446 | cortex | NOX2 | CCK | 0.05 | 1.09 | GSE36446 | cortex | NOX2 |  |
| SOS1 | 0.04 | 0.92 | GSE36446 | cortex | NOX2 | TIMP1 | 0.02 | 2.93 | GSE36446 | medulla | NOX2 |  |
| PTPN4 | 0.03 | 0.90 | GSE36446 | cortex | NOX2 | MFGE8 | 0.00 | 2.20 | GSE36446 | medulla | NOX2 |  |
| LMAN1 | 0.01 | 0.89 | GSE36446 | cortex | NOX2 | GJA1 | 0.01 | 1.73 | GSE36446 | medulla | NOX2 |  |
| SLC4A7 | 0.00 | 0.88 | GSE36446 | cortex | NOX2 | ICAM1 | 0.01 | 1.70 | GSE36446 | medulla | NOX2 |  |
| FUBP1 | 0.04 | 0.88 | GSE36446 | cortex | NOX2 | Tpm1 | 0.04 | 1.50 | GSE36446 | medulla | NOX2 |  |
| POLR3F | 0.00 | 0.86 | GSE36446 | cortex | NOX2 | GSPT1 | 0.00 | 1.32 | GSE36446 | medulla | NOX2 |  |
| IRGQ | 0.00 | 0.86 | GSE36446 | cortex | NOX2 | ILK | 0.00 | 1.30 | GSE36446 | medulla | NOX2 |  |
| COPZ2 | 0.01 | 0.85 | GSE36446 | cortex | NOX2 | PDPK1 | 0.01 | 1.28 | GSE36446 | medulla | NOX2 |  |
| CCNG2 | 0.00 | 0.84 | GSE36446 | cortex | NOX2 | TJP1 | 0.02 | 1.26 | GSE36446 | medulla | NOX2 |  |
| GMCL1 | 0.00 | 0.84 | GSE36446 | cortex | NOX2 | PLCG1 | 0.00 | 1.25 | GSE36446 | medulla | NOX2 |  |
| PARVA | 0.00 | 0.84 | GSE36446 | cortex | NOX2 | CHD8 | 0.02 | 1.22 | GSE36446 | medulla | NOX2 |  |
| MMP13 | 0.01 | 0.83 | GSE36446 | cortex | NOX2 | DYRK2 | 0.02 | 1.13 | GSE36446 | medulla | NOX2 |  |
| PFKFB3 | 0.00 | 0.82 | GSE36446 | cortex | NOX2 |  |  |  |  |  |  |  |
| ACSL1 | 0.04 | 0.82 | GSE36446 | cortex | NOX2 |  |  |  |  |  |  |  |
| EXOC3L4 | 0.00 | 0.82 | GSE36446 | cortex | NOX2 |  |  |  |  |  |  |  |
| STRN3 | 0.02 | 0.81 | GSE36446 | cortex | NOX2 |  |  |  |  |  |  |  |
| COQ10B | 0.02 | 0.79 | GSE36446 | cortex | NOX2 |  |  |  |  |  |  |  |
| RNF19A | 0.00 | 0.78 | GSE36446 | cortex | NOX2 |  |  |  |  |  |  |  |
| TBPL1 | 0.00 | 0.76 | GSE36446 | cortex | NOX2 |  |  |  |  |  |  |  |
| ELL2 | 0.00 | 0.74 | GSE36446 | cortex | NOX2 |  |  |  |  |  |  |  |
| PTPN4 | 0.03 | 0.93 | GSE36446 | medulla | NOX2 |  |  |  |  |  |  |  |
| TBPL1 | 0.01 | 0.92 | GSE36446 | medulla | NOX2 |  |  |  |  |  |  |  |
| SSR1 | 0.01 | 0.90 | GSE36446 | medulla | NOX2 |  |  |  |  |  |  |  |
| IRGQ | 0.01 | 0.90 | GSE36446 | medulla | NOX2 |  |  |  |  |  |  |  |
| POLR3D | 0.01 | 0.89 | GSE36446 | medulla | NOX2 |  |  |  |  |  |  |  |
| PFKFB3 | 0.01 | 0.89 | GSE36446 | medulla | NOX2 |  |  |  |  |  |  |  |
| SOS1 | 0.01 | 0.88 | GSE36446 | medulla | NOX2 |  |  |  |  |  |  |  |
| PPFIBP1 | 0.05 | 0.88 | GSE36446 | medulla | NOX2 |  |  |  |  |  |  |  |
| RNF19A | 0.01 | 0.87 | GSE36446 | medulla | NOX2 |  |  |  |  |  |  |  |
| MXD1 | 0.02 | 0.85 | GSE36446 | medulla | NOX2 |  |  |  |  |  |  |  |
| CCNG2 | 0.03 | 0.85 | GSE36446 | medulla | NOX2 |  |  |  |  |  |  |  |
| PARVA | 0.00 | 0.84 | GSE36446 | medulla | NOX2 |  |  |  |  |  |  |  |
| MAP3K14 | 0.00 | 0.78 | GSE36446 | medulla | NOX2 |  |  |  |  |  |  |  |
| ARHGEF1 | 0.00 | 0.77 | GSE36446 | medulla | NOX2 |  |  |  |  |  |  |  |
| PPP1R10 | 0.00 | 0.76 | GSE36446 | medulla | NOX2 |  |  |  |  |  |  |  |
| CCK | 0.00 | 0.75 | GSE36446 | medulla | NOX2 |  |  |  |  |  |  |  |
| TRPS1 | 0.01 | 0.75 | GSE36446 | medulla | NOX2 |  |  |  |  |  |  |  |
| ACSL1 | 0.04 | 0.74 | GSE36446 | medulla | NOX2 |  |  |  |  |  |  |  |
| H1f0 | 0.02 | 0.71 | GSE36446 | medulla | NOX2 |  |  |  |  |  |  |  |

**Supplemental Table 17B. LIUS-downregulated genes in bone marrow cells are classified into four groups, reactive oxygen species (ROS)-dependent; ROS-suppressed; ROS-dependent/suppressed; and ROS-independent.** 110 out of total 182 (60.44%) downregulated genes in LIUS-treated bone marrow cells are identified as the ROS-dependent downregulations, which were upregulated in Nrf2-knocked out dataset (GSE7810) or/and downregulated in NOX2-knocked out dataset (GSE35446). Meanwhile, 46 of 182 genes (25.27%) are identified as the ROS-suppressed downregulations. 21 of 182 genes (11.54%) shared are shown in this table.

| Down regulated genes in LIUS-treated bone marrow cells | | | | | | | | | | | | |
| --- | --- | --- | --- | --- | --- | --- | --- | --- | --- | --- | --- | --- |
| ROS promoter genes | p value | Fold Change | dataset | tissue | KO/Inhibit Target | ROS inhibitor genes | p value | Fold Change | dataset | tissue | KO/Inhibit Target | Dual |
| CCL22 | 0.00 | 34.01 | GSE7810 | liver | Nrf2 | BID | 0.04 | 0.77 | GSE7810 | liver | Nrf2 | APOBEC1 |
| CCL11 | 0.00 | 18.55 | GSE7810 | liver | Nrf2 | HMOX1 | 0.05 | 0.76 | GSE7810 | liver | Nrf2 | CD48 |
| NOS2 | 0.00 | 11.65 | GSE7810 | liver | Nrf2 | TGIF1 | 0.04 | 0.74 | GSE7810 | liver | Nrf2 | FCER1G |
| Clec4a3 | 0.00 | 5.60 | GSE7810 | liver | Nrf2 | CDK6 | 0.01 | 0.65 | GSE7810 | liver | Nrf2 | SYK |
| MAP2K7 | 0.05 | 5.44 | GSE7810 | liver | Nrf2 | WFDC2 | 0.01 | 0.64 | GSE7810 | liver | Nrf2 | PTPN6 |
| IL10RA | 0.02 | 5.10 | GSE7810 | liver | Nrf2 | DCBLD2 | 0.02 | 0.58 | GSE7810 | liver | Nrf2 | ITGAM |
| C3AR1 | 0.00 | 4.34 | GSE7810 | liver | Nrf2 | THBD | 0.00 | 0.57 | GSE7810 | liver | Nrf2 | NEIL3 |
| CASP4 | 0.00 | 4.12 | GSE7810 | liver | Nrf2 | WARS | 0.03 | 0.57 | GSE7810 | liver | Nrf2 | CKLF |
| C5AR1 | 0.00 | 3.29 | GSE7810 | liver | Nrf2 | SLC30A1 | 0.01 | 0.54 | GSE7810 | liver | Nrf2 | LYL1 |
| HGF | 0.00 | 3.29 | GSE7810 | liver | Nrf2 | CREG1 | 0.00 | 0.50 | GSE7810 | liver | Nrf2 | MTSS1 |
| APOBEC1 | 0.00 | 3.21 | GSE7810 | liver | Nrf2 | DUSP4 | 0.00 | 0.44 | GSE7810 | liver | Nrf2 | TCF19 |
| SLC16A6 | 0.01 | 3.21 | GSE7810 | liver | Nrf2 | INHBA | 0.02 | 0.42 | GSE7810 | liver | Nrf2 | CCR1 |
| SAMSN1 | 0.00 | 3.20 | GSE7810 | liver | Nrf2 | CDH1 | 0.01 | 0.40 | GSE7810 | liver | Nrf2 | EPS15 |
| RGS1 | 0.00 | 2.97 | GSE7810 | liver | Nrf2 | GCA | 0.01 | 0.40 | GSE7810 | liver | Nrf2 | TLR6 |
| MAFB | 0.00 | 2.91 | GSE7810 | liver | Nrf2 | ITGA6 | 0.00 | 0.35 | GSE7810 | liver | Nrf2 | RCSD1 |
| Ifitm1 | 0.00 | 2.87 | GSE7810 | liver | Nrf2 | CENPA | 0.00 | 0.16 | GSE7810 | liver | Nrf2 | SLC30A1 |
| EBI3 | 0.00 | 2.73 | GSE7810 | liver | Nrf2 | LIPA | 0.00 | 0.15 | GSE7810 | liver | Nrf2 | CENPA |
| RSAD2 | 0.00 | 2.71 | GSE7810 | liver | Nrf2 | LYN | 0.00 | 1.15 | GSE36446 | cortex | NOX2 | CDK6 |
| MSR1 | 0.00 | 2.59 | GSE7810 | liver | Nrf2 | MTSS1 | 0.01 | 1.20 | GSE36446 | cortex | NOX2 | CREG1 |
| CASP1 | 0.00 | 2.46 | GSE7810 | liver | Nrf2 | CCDC39 | 0.00 | 1.20 | GSE36446 | cortex | NOX2 | CDH1 |
| LMO2 | 0.00 | 2.46 | GSE7810 | liver | Nrf2 | VAT1 | 0.04 | 1.21 | GSE36446 | cortex | NOX2 |  |
| PTPRJ | 0.04 | 2.46 | GSE7810 | liver | Nrf2 | SYK | 0.00 | 1.25 | GSE36446 | cortex | NOX2 |  |
| IL10 | 0.00 | 2.43 | GSE7810 | liver | Nrf2 | DCBLD2 | 0.00 | 1.33 | GSE36446 | cortex | NOX2 |  |
| Samd9l | 0.00 | 2.36 | GSE7810 | liver | Nrf2 | SEC14L1 | 0.00 | 1.36 | GSE36446 | cortex | NOX2 |  |
| FOS | 0.00 | 2.34 | GSE7810 | liver | Nrf2 | CCR1 | 0.02 | 1.97 | GSE36446 | medulla | NOX2 |  |
| PSMB9 | 0.00 | 2.32 | GSE7810 | liver | Nrf2 | RRM2 | 0.00 | 1.77 | GSE36446 | medulla | NOX2 |  |
| ARHGAP25 | 0.00 | 2.29 | GSE7810 | liver | Nrf2 | CDK1 | 0.00 | 1.74 | GSE36446 | medulla | NOX2 |  |
| SFTPC | 0.04 | 2.25 | GSE7810 | liver | Nrf2 | LYL1 | 0.01 | 1.71 | GSE36446 | medulla | NOX2 |  |
| WAS | 0.00 | 2.21 | GSE7810 | liver | Nrf2 | CD48 | 0.00 | 1.57 | GSE36446 | medulla | NOX2 |  |
| CD83 | 0.00 | 2.18 | GSE7810 | liver | Nrf2 | IFI30 | 0.00 | 1.51 | GSE36446 | medulla | NOX2 |  |
| APOE | 0.01 | 2.18 | GSE7810 | liver | Nrf2 | TGIF1 | 0.04 | 1.49 | GSE36446 | medulla | NOX2 |  |
| UCP2 | 0.00 | 2.16 | GSE7810 | liver | Nrf2 | HMOX1 | 0.02 | 1.45 | GSE36446 | medulla | NOX2 |  |
| GBP2 | 0.00 | 2.14 | GSE7810 | liver | Nrf2 | WARS | 0.00 | 1.39 | GSE36446 | medulla | NOX2 |  |
| CD48 | 0.00 | 2.13 | GSE7810 | liver | Nrf2 | IFNGR1 | 0.00 | 1.36 | GSE36446 | medulla | NOX2 |  |
| FCER1G | 0.00 | 2.10 | GSE7810 | liver | Nrf2 | PTPN6 | 0.00 | 1.35 | GSE36446 | medulla | NOX2 |  |
| RNF213 | 0.00 | 2.05 | GSE7810 | liver | Nrf2 | IRF5 | 0.04 | 1.33 | GSE36446 | medulla | NOX2 |  |
| SYK | 0.00 | 2.04 | GSE7810 | liver | Nrf2 | CASP4 | 0.04 | 1.33 | GSE36446 | medulla | NOX2 |  |
| IL1B | 0.00 | 2.03 | GSE7810 | liver | Nrf2 | APOBEC1 | 0.04 | 1.32 | GSE36446 | medulla | NOX2 |  |
| Slfn2 | 0.00 | 2.01 | GSE7810 | liver | Nrf2 | CKLF | 0.00 | 1.32 | GSE36446 | medulla | NOX2 |  |
| SH3BP5 | 0.00 | 1.98 | GSE7810 | liver | Nrf2 | FCER1G | 0.05 | 1.29 | GSE36446 | medulla | NOX2 |  |
| PTPN6 | 0.00 | 1.95 | GSE7810 | liver | Nrf2 | SCAP | 0.00 | 1.29 | GSE36446 | medulla | NOX2 |  |
| EGR1 | 0.00 | 1.94 | GSE7810 | liver | Nrf2 | TLR6 | 0.00 | 1.28 | GSE36446 | medulla | NOX2 |  |
| ITGAM | 0.00 | 1.93 | GSE7810 | liver | Nrf2 | IL1RL1 | 0.02 | 1.25 | GSE36446 | medulla | NOX2 |  |
| TLR2 | 0.00 | 1.92 | GSE7810 | liver | Nrf2 | RCSD1 | 0.04 | 1.24 | GSE36446 | medulla | NOX2 |  |
| APOBR | 0.00 | 1.92 | GSE7810 | liver | Nrf2 | ITGAM | 0.03 | 1.24 | GSE36446 | medulla | NOX2 |  |
| CD14 | 0.00 | 1.92 | GSE7810 | liver | Nrf2 | CCL20 | 0.04 | 1.23 | GSE36446 | medulla | NOX2 |  |
| CD47 | 0.00 | 1.84 | GSE7810 | liver | Nrf2 | TCF19 | 0.00 | 1.20 | GSE36446 | medulla | NOX2 |  |
| NR1H3 | 0.03 | 1.84 | GSE7810 | liver | Nrf2 | LYN | 0.00 | 1.18 | GSE36446 | medulla | NOX2 |  |
| DOCK8 | 0.00 | 1.82 | GSE7810 | liver | Nrf2 | BID | 0.05 | 1.15 | GSE36446 | medulla | NOX2 |  |
| NEIL3 | 0.02 | 1.78 | GSE7810 | liver | Nrf2 | NEIL3 | 0.01 | 1.14 | GSE36446 | medulla | NOX2 |  |
| TLR7 | 0.00 | 1.77 | GSE7810 | liver | Nrf2 | EPS15 | 0.02 | 1.14 | GSE36446 | medulla | NOX2 |  |
| CKLF | 0.00 | 1.77 | GSE7810 | liver | Nrf2 | BCL10 | 0.04 | 1.13 | GSE36446 | medulla | NOX2 |  |
| ATF3 | 0.00 | 1.72 | GSE7810 | liver | Nrf2 | CCDC39 | 0.04 | 1.12 | GSE36446 | medulla | NOX2 |  |
| MAF | 0.01 | 1.72 | GSE7810 | liver | Nrf2 |  |  |  |  |  |  |  |
| NPR3 | 0.01 | 1.71 | GSE7810 | liver | Nrf2 |  |  |  |  |  |  |  |
| CD68 | 0.00 | 1.70 | GSE7810 | liver | Nrf2 |  |  |  |  |  |  |  |
| ALCAM | 0.05 | 1.70 | GSE7810 | liver | Nrf2 |  |  |  |  |  |  |  |
| LYL1 | 0.03 | 1.69 | GSE7810 | liver | Nrf2 |  |  |  |  |  |  |  |
| CCL2 | 0.00 | 1.69 | GSE7810 | liver | Nrf2 |  |  |  |  |  |  |  |
| SLAMF7 | 0.02 | 1.68 | GSE7810 | liver | Nrf2 |  |  |  |  |  |  |  |

(Continued…)

| ROS promoter genes | p value | Fold Change | dataset | tissue | KO/Inhibit Target | ROS inhibitor genes | p value | Fold Change | dataset | tissue | KO/Inhibit Target | Dual |
| --- | --- | --- | --- | --- | --- | --- | --- | --- | --- | --- | --- | --- |
| PELI2 | 0.02 | 1.66 | GSE7810 | liver | Nrf2 |  |  |  |  |  |  |  |
| MTSS1 | 0.01 | 1.64 | GSE7810 | liver | Nrf2 |  |  |  |  |  |  |  |
| AMPD3 | 0.01 | 1.63 | GSE7810 | liver | Nrf2 |  |  |  |  |  |  |  |
| ICAM2 | 0.02 | 1.62 | GSE7810 | liver | Nrf2 |  |  |  |  |  |  |  |
| TCF19 | 0.00 | 1.62 | GSE7810 | liver | Nrf2 |  |  |  |  |  |  |  |
| CCR1 | 0.00 | 1.61 | GSE7810 | liver | Nrf2 |  |  |  |  |  |  |  |
| Tnfsf9 | 0.01 | 1.61 | GSE7810 | liver | Nrf2 |  |  |  |  |  |  |  |
| C3 | 0.00 | 1.61 | GSE7810 | liver | Nrf2 |  |  |  |  |  |  |  |
| IL7R | 0.01 | 1.59 | GSE7810 | liver | Nrf2 |  |  |  |  |  |  |  |
| IFITM2 | 0.00 | 1.59 | GSE7810 | liver | Nrf2 |  |  |  |  |  |  |  |
| CSF1R | 0.00 | 1.58 | GSE7810 | liver | Nrf2 |  |  |  |  |  |  |  |
| EPS15 | 0.01 | 1.58 | GSE7810 | liver | Nrf2 |  |  |  |  |  |  |  |
| ADCY3 | 0.03 | 1.58 | GSE7810 | liver | Nrf2 |  |  |  |  |  |  |  |
| EIF2AK2 | 0.01 | 1.53 | GSE7810 | liver | Nrf2 |  |  |  |  |  |  |  |
| TLR4 | 0.01 | 1.53 | GSE7810 | liver | Nrf2 |  |  |  |  |  |  |  |
| SPRY2 | 0.01 | 1.53 | GSE7810 | liver | Nrf2 |  |  |  |  |  |  |  |
| TLR6 | 0.01 | 1.52 | GSE7810 | liver | Nrf2 |  |  |  |  |  |  |  |
| NMI | 0.01 | 1.50 | GSE7810 | liver | Nrf2 |  |  |  |  |  |  |  |
| HCK | 0.01 | 1.50 | GSE7810 | liver | Nrf2 |  |  |  |  |  |  |  |
| Ccl9 | 0.01 | 1.42 | GSE7810 | liver | Nrf2 |  |  |  |  |  |  |  |
| CH25H | 0.02 | 1.40 | GSE7810 | liver | Nrf2 |  |  |  |  |  |  |  |
| ITPKB | 0.03 | 1.37 | GSE7810 | liver | Nrf2 |  |  |  |  |  |  |  |
| VCAM1 | 0.03 | 1.33 | GSE7810 | liver | Nrf2 |  |  |  |  |  |  |  |
| RCSD1 | 0.03 | 1.32 | GSE7810 | liver | Nrf2 |  |  |  |  |  |  |  |
| IGFBP4 | 0.03 | 1.30 | GSE7810 | liver | Nrf2 |  |  |  |  |  |  |  |
| FOS | 0.02 | 0.60 | GSE36446 | cortex | NOX2 |  |  |  |  |  |  |  |
| SLC30A1 | 0.00 | 0.70 | GSE36446 | cortex | NOX2 |  |  |  |  |  |  |  |
| TNFSF13 | 0.00 | 0.76 | GSE36446 | cortex | NOX2 |  |  |  |  |  |  |  |
| AKAP10 | 0.00 | 0.78 | GSE36446 | cortex | NOX2 |  |  |  |  |  |  |  |
| NEURL3 | 0.01 | 0.78 | GSE36446 | cortex | NOX2 |  |  |  |  |  |  |  |
| EIF2AK2 | 0.02 | 0.79 | GSE36446 | cortex | NOX2 |  |  |  |  |  |  |  |
| SFTPC | 0.00 | 0.80 | GSE36446 | cortex | NOX2 |  |  |  |  |  |  |  |
| IRF4 | 0.00 | 0.80 | GSE36446 | cortex | NOX2 |  |  |  |  |  |  |  |
| ELOVL3 | 0.00 | 0.81 | GSE36446 | cortex | NOX2 |  |  |  |  |  |  |  |
| TRADD | 0.00 | 0.81 | GSE36446 | cortex | NOX2 |  |  |  |  |  |  |  |
| RFFL | 0.00 | 0.82 | GSE36446 | cortex | NOX2 |  |  |  |  |  |  |  |
| PIM1 | 0.02 | 0.82 | GSE36446 | cortex | NOX2 |  |  |  |  |  |  |  |
| NHLRC1 | 0.00 | 0.85 | GSE36446 | cortex | NOX2 |  |  |  |  |  |  |  |
| CD163 | 0.01 | 0.85 | GSE36446 | cortex | NOX2 |  |  |  |  |  |  |  |
| NPR3 | 0.00 | 0.85 | GSE36446 | cortex | NOX2 |  |  |  |  |  |  |  |
| CENPA | 0.00 | 0.86 | GSE36446 | cortex | NOX2 |  |  |  |  |  |  |  |
| ICAM2 | 0.00 | 0.86 | GSE36446 | cortex | NOX2 |  |  |  |  |  |  |  |
| NEIL3 | 0.00 | 0.87 | GSE36446 | cortex | NOX2 |  |  |  |  |  |  |  |
| C3AR1 | 0.01 | 0.87 | GSE36446 | cortex | NOX2 |  |  |  |  |  |  |  |
| IGFBP4 | 0.01 | 0.87 | GSE36446 | cortex | NOX2 |  |  |  |  |  |  |  |
| IL10RA | 0.01 | 0.88 | GSE36446 | cortex | NOX2 |  |  |  |  |  |  |  |
| IL10 | 0.03 | 0.89 | GSE36446 | cortex | NOX2 |  |  |  |  |  |  |  |
| CAMP | 0.04 | 0.90 | GSE36446 | cortex | NOX2 |  |  |  |  |  |  |  |
| PPP1R3C | 0.02 | 0.92 | GSE36446 | cortex | NOX2 |  |  |  |  |  |  |  |
| IGFBP4 | 0.01 | 0.90 | GSE36446 | medulla | NOX2 |  |  |  |  |  |  |  |
| AKAP10 | 0.02 | 0.89 | GSE36446 | medulla | NOX2 |  |  |  |  |  |  |  |
| LAMC3 | 0.01 | 0.87 | GSE36446 | medulla | NOX2 |  |  |  |  |  |  |  |
| MAP3K3 | 0.04 | 0.87 | GSE36446 | medulla | NOX2 |  |  |  |  |  |  |  |
| CDK6 | 0.02 | 0.87 | GSE36446 | medulla | NOX2 |  |  |  |  |  |  |  |
| ZMYND8 | 0.04 | 0.86 | GSE36446 | medulla | NOX2 |  |  |  |  |  |  |  |
| EIF2AK2 | 0.04 | 0.83 | GSE36446 | medulla | NOX2 |  |  |  |  |  |  |  |
| ITPKB | 0.00 | 0.82 | GSE36446 | medulla | NOX2 |  |  |  |  |  |  |  |
| RFFL | 0.00 | 0.80 | GSE36446 | medulla | NOX2 |  |  |  |  |  |  |  |
| MOSPD2 | 0.00 | 0.79 | GSE36446 | medulla | NOX2 |  |  |  |  |  |  |  |
| SET | 0.00 | 0.75 | GSE36446 | medulla | NOX2 |  |  |  |  |  |  |  |
| CREG1 | 0.04 | 0.73 | GSE36446 | medulla | NOX2 |  |  |  |  |  |  |  |
| ARC | 0.01 | 0.73 | GSE36446 | medulla | NOX2 |  |  |  |  |  |  |  |
| PTK2B | 0.00 | 0.71 | GSE36446 | medulla | NOX2 |  |  |  |  |  |  |  |
| CDH1 | 0.00 | 0.61 | GSE36446 | medulla | NOX2 |  |  |  |  |  |  |  |
| SLC30A1 | 0.00 | 0.56 | GSE36446 | medulla | NOX2 |  |  |  |  |  |  |  |

**Supplemental Table 17C. LIUS-upregulated genes in lymphoma cells are classified into four groups, reactive oxygen species (ROS)-dependent; ROS-suppressed; ROS-dependent/suppressed; and ROS-independent.** 34 out of total 77 (44.16%) upregulated genes in LIUS-treated lymphoma cell are identified as the ROS-dependent upregulations, which were upregulated in Nrf2-knocked out dataset (GSE7810) or/and downregulated in NOX2-knocked out dataset (GSE35446). Meanwhile, 21 of 77 genes (27.27%) are identified as the ROS-suppressed upregulations. 5 out of 77 genes (6.49%) are shared.

| Up-regulated in lymphoma | | | | | | | | | | | | |
| --- | --- | --- | --- | --- | --- | --- | --- | --- | --- | --- | --- | --- |
| ROS promoter genes | p value | Fold Change | dataset | tissue | KO/Inhibit Target | ROS inhibitor genes | p value | Fold Change | dataset | tissue | KO/Inhibit Target | Dual |
| XIST | 0.00 | 19.67 | GSE7810 | liver | Nrf2 | HMOX1 | 0.05 | 0.76 | GSE7810 | liver | Nrf2 | CCR1 |
| NOS2 | 0.00 | 11.65 | GSE7810 | liver | Nrf2 | CD40 | 0.01 | 0.71 | GSE7810 | liver | Nrf2 | Tsc22d3 |
| TRAF1 | 0.00 | 4.06 | GSE7810 | liver | Nrf2 | MAFF | 0.05 | 0.71 | GSE7810 | liver | Nrf2 | KLF6 |
| ADM | 0.00 | 3.73 | GSE7810 | liver | Nrf2 | CAPN2 | 0.01 | 0.68 | GSE7810 | liver | Nrf2 | CD40 |
| Sp100 | 0.00 | 3.20 | GSE7810 | liver | Nrf2 | ICAM1 | 0.00 | 0.60 | GSE7810 | liver | Nrf2 | MAFF |
| RGS1 | 0.00 | 2.97 | GSE7810 | liver | Nrf2 | EZR | 0.00 | 0.57 | GSE7810 | liver | Nrf2 |  |
| MAFB | 0.00 | 2.91 | GSE7810 | liver | Nrf2 | DUSP4 | 0.00 | 0.44 | GSE7810 | liver | Nrf2 |  |
| CXCL10 | 0.00 | 2.60 | GSE7810 | liver | Nrf2 | F3 | 0.00 | 0.44 | GSE7810 | liver | Nrf2 |  |
| ELL2 | 0.05 | 2.51 | GSE7810 | liver | Nrf2 | NR4A3 | 0.03 | 0.29 | GSE7810 | liver | Nrf2 |  |
| IL10 | 0.00 | 2.43 | GSE7810 | liver | Nrf2 | KLF6 | 0.02 | 1.34 | GSE36446 | cortex | NOX2 |  |
| PTX3 | 0.00 | 2.11 | GSE7810 | liver | Nrf2 | IER3 | 0.03 | 1.25 | GSE36446 | cortex | NOX2 |  |
| Ccl8 | 0.00 | 1.91 | GSE7810 | liver | Nrf2 | MFGE8 | 0.00 | 2.20 | GSE36446 | medulla | NOX2 |  |
| RGS2 | 0.00 | 1.90 | GSE7810 | liver | Nrf2 | CCR1 | 0.02 | 1.97 | GSE36446 | medulla | NOX2 |  |
| GPX3 | 0.00 | 1.87 | GSE7810 | liver | Nrf2 | CD44 | 0.04 | 1.84 | GSE36446 | medulla | NOX2 |  |
| ADCY2 | 0.01 | 1.85 | GSE7810 | liver | Nrf2 | F3 | 0.01 | 1.78 | GSE36446 | medulla | NOX2 |  |
| CCL2 | 0.00 | 1.69 | GSE7810 | liver | Nrf2 | ICAM1 | 0.01 | 1.70 | GSE36446 | medulla | NOX2 |  |
| SLAMF7 | 0.02 | 1.68 | GSE7810 | liver | Nrf2 | SGK1 | 0.01 | 1.69 | GSE36446 | medulla | NOX2 |  |
| BMP2 | 0.05 | 1.63 | GSE7810 | liver | Nrf2 | HMOX1 | 0.02 | 1.45 | GSE36446 | medulla | NOX2 |  |
| CCR1 | 0.00 | 1.61 | GSE7810 | liver | Nrf2 | SERPINF1 | 0.03 | 1.39 | GSE36446 | medulla | NOX2 |  |
| C3 | 0.00 | 1.61 | GSE7810 | liver | Nrf2 | Tsc22d3 | 0.02 | 1.33 | GSE36446 | medulla | NOX2 |  |
| IL7R | 0.01 | 1.59 | GSE7810 | liver | Nrf2 | GSTM5 | 0.02 | 1.31 | GSE36446 | medulla | NOX2 |  |
| ZFP36L1 | 0.01 | 1.50 | GSE7810 | liver | Nrf2 | SCARB1 | 0.03 | 1.27 | GSE36446 | medulla | NOX2 |  |
| Tsc22d3 | 0.01 | 1.48 | GSE7810 | liver | Nrf2 | IL1RL1 | 0.02 | 1.25 | GSE36446 | medulla | NOX2 |  |
| KLF6 | 0.03 | 1.41 | GSE7810 | liver | Nrf2 | CCL20 | 0.04 | 1.23 | GSE36446 | medulla | NOX2 |  |
| FYN | 0.05 | 1.34 | GSE7810 | liver | Nrf2 | NR4A3 | 0.05 | 1.10 | GSE36446 | medulla | NOX2 |  |
| FTH1 | 0.04 | 1.32 | GSE7810 | liver | Nrf2 |  |  |  |  |  |  |  |
| MERTK | 0.05 | 1.27 | GSE7810 | liver | Nrf2 |  |  |  |  |  |  |  |
| IL10 | 0.03 | 0.89 | GSE36446 | cortex | NOX2 |  |  |  |  |  |  |  |
| CD40 | 0.01 | 0.85 | GSE36446 | cortex | NOX2 |  |  |  |  |  |  |  |
| Rasal2 | 0.00 | 0.82 | GSE36446 | cortex | NOX2 |  |  |  |  |  |  |  |
| PIM1 | 0.02 | 0.82 | GSE36446 | cortex | NOX2 |  |  |  |  |  |  |  |
| FABP7 | 0.00 | 0.80 | GSE36446 | cortex | NOX2 |  |  |  |  |  |  |  |
| ELL2 | 0.00 | 0.74 | GSE36446 | cortex | NOX2 |  |  |  |  |  |  |  |
| LAMC3 | 0.01 | 0.87 | GSE36446 | medulla | NOX2 |  |  |  |  |  |  |  |
| MAFF | 0.00 | 0.71 | GSE36446 | medulla | NOX2 |  |  |  |  |  |  |  |
| MAFK | 0.00 | 0.70 | GSE36446 | medulla | NOX2 |  |  |  |  |  |  |  |

**Supplemental 17D.** **LIUS-downregulated genes in lymphoma cells are classified into four groups, reactive oxygen species (ROS)-dependent; ROS-suppressed; ROS-dependent/suppressed; and ROS-independent.** 17 out of total 39 (43.59%) down-regulated genes in LIUS-treated Lymphoma cells are identified as ROS-dependent downregulations which can be upregulated in Nrf2-knocked out dataset (GSE7810) or/and downregulated in NOX2-knocked out dataset (GSE35446). Meanwhile, 9 out of 39 genes (23.08%) are identified as ROS-suppressed downregulations. One gene (2.56%) shared are shown in this table.

| Downregulated gene in Lymphoma | | | | | | | | | | | | |
| --- | --- | --- | --- | --- | --- | --- | --- | --- | --- | --- | --- | --- |
| ROS promoter genes | p value | Fold Change | dataset | tissue | KO/Inhibit Targe | ROS inhibitor genes | p value | Fold Change | dataset | tissue | KO/Inhibit Targe | Dual |
| LRP2BP | 0.00 | 8.31 | GSE7810 | liver | Nrf2 | CHAC1 | 0.0344 | 0.72 | GSE7810 | liver | Nrf2 | MTSS1 |
| EZH1 | 0.04 | 2.88 | GSE7810 | liver | Nrf2 | CSF3 | 0.047 | 0.71 | GSE7810 | liver | Nrf2 |  |
| TLR1 | 0.00 | 2.73 | GSE7810 | liver | Nrf2 | Tnik | 0.00643 | 0.66 | GSE7810 | liver | Nrf2 |  |
| SFTPC | 0.04 | 2.25 | GSE7810 | liver | Nrf2 | GJA1 | 0.0238 | 0.58 | GSE7810 | liver | Nrf2 |  |
| LPAR1 | 0.01 | 1.90 | GSE7810 | liver | Nrf2 | Pvr | 0.00052 | 0.44 | GSE7810 | liver | Nrf2 |  |
| PML | 0.00 | 1.90 | GSE7810 | liver | Nrf2 | HBEGF | 0.000151 | 0.33 | GSE7810 | liver | Nrf2 |  |
| CD47 | 0.00 | 1.84 | GSE7810 | liver | Nrf2 | ABTB2 | 0.035 | 0.33 | GSE7810 | liver | Nrf2 |  |
| TLR7 | 0.00 | 1.77 | GSE7810 | liver | Nrf2 | MTSS1 | 0.0148 | 1.20 | GSE36446 | cortex | NOX2 |  |
| MTSS1 | 0.01 | 1.64 | GSE7810 | liver | Nrf2 | GJA1 | 0.00781 | 1.73 | GSE36446 | medulla | NOX2 |  |
| AFF1 | 0.04 | 0.89 | GSE36446 | cortex | NOX2 | Pvr | 0.0428 | 1.68 | GSE36446 | medulla | NOX2 |  |
| DUSP2 | 0.00 | 0.87 | GSE36446 | cortex | NOX2 | Tnik | 0.012 | 1.59 | GSE36446 | medulla | NOX2 |  |
| CCNG2 | 0.00 | 0.84 | GSE36446 | cortex | NOX2 | ITGB1 | 0.0246 | 1.23 | GSE36446 | medulla | NOX2 |  |
| EZH1 | 0.03 | 0.82 | GSE36446 | cortex | NOX2 |  |  |  |  |  |  |  |
| SFTPC | 0.00 | 0.80 | GSE36446 | cortex | NOX2 |  |  |  |  |  |  |  |
| BBX | 0.02 | 0.86 | GSE36446 | medulla | NOX2 |  |  |  |  |  |  |  |
| PTPN11 | 0.00 | 0.86 | GSE36446 | medulla | NOX2 |  |  |  |  |  |  |  |
| CCNG2 | 0.03 | 0.85 | GSE36446 | medulla | NOX2 |  |  |  |  |  |  |  |
| EZH1 | 0.00 | 0.79 | GSE36446 | medulla | NOX2 |  |  |  |  |  |  |  |
| ARHGDIA | 0.00 | 0.79 | GSE36446 | medulla | NOX2 |  |  |  |  |  |  |  |
| MAPK8IP1 | 0.05 | 0.76 | GSE36446 | medulla | NOX2 |  |  |  |  |  |  |  |
| ZFP36L2 | 0.00 | 0.56 | GSE36446 | medulla | NOX2 |  |  |  |  |  |  |  |

**Supplemental Table 17E. LIUS-upregulated genes in pre-osteoblast cells are classified into four groups, reactive oxygen species (ROS)-dependent; ROS-suppressed; ROS-dependent/suppressed; and ROS-independent.** Nine out of total 21 (42.86%) upregulated genes in LIUS-treated pre-osteoblast are identified as the ROS-dependent upregulations, which were upregulated in Nrf2-knocked out dataset (GSE7810) or/and downregulated in NOX2-knocked out dataset (GSE35446). Meanwhile, 6 out of 21 (28.57%) genes are identified as the ROS-suppressed upregulations. Two genes (9.52%) are shared.

| Upregulate in Pre-osteoblast | | | | | | | | | | | | |
| --- | --- | --- | --- | --- | --- | --- | --- | --- | --- | --- | --- | --- |
| ROS promoter genes | p value | Fold Change | dataset | tissue | KO/Inhibit Target | ROS inhibitor genes | p value | Fold Change | dataset | tissue | KO/Inhibit Target | Dual |
| CDK5R1 | 0.02 | 2.36 | GSE7810 | liver | Nrf2 | TFPI | 0.0349 | 0.78 | GSE7810 | liver | Nrf2 | ADAMTS1 |
| RGS2 | 0.00 | 1.90 | GSE7810 | liver | Nrf2 | EGR3 | 0.00649 | 0.35 | GSE7810 | liver | Nrf2 | TFPI |
| DBP | 0.00 | 1.68 | GSE7810 | liver | Nrf2 | MYC | 0.0108 | 1.63 | GSE36446 | cortex | NOX2 |  |
| MMP13 | 0.02 | 1.52 | GSE7810 | liver | Nrf2 | ADAMTS1 | 0.0061 | 2.30 | GSE36446 | medulla | NOX2 |  |
| CH25H | 0.02 | 1.40 | GSE7810 | liver | Nrf2 | MYC | 0.00234 | 2.17 | GSE36446 | medulla | NOX2 |  |
| MMP9 | 0.03 | 1.39 | GSE7810 | liver | Nrf2 | Tpm1 | 0.0423 | 1.50 | GSE36446 | medulla | NOX2 |  |
| IGFBP4 | 0.03 | 1.30 | GSE7810 | liver | Nrf2 | SRF | 0.00988 | 1.14 | GSE36446 | medulla | NOX2 |  |
| ADAMTS1 | 0.05 | 1.30 | GSE7810 | liver | Nrf2 |  |  |  |  |  |  |  |
| IGFBP4 | 0.01 | 0.87 | GSE36446 | cortex | NOX2 |  |  |  |  |  |  |  |
| MMP13 | 0.01 | 0.83 | GSE36446 | cortex | NOX2 |  |  |  |  |  |  |  |
| TFPI | 0.00 | 0.76 | GSE36446 | cortex | NOX2 |  |  |  |  |  |  |  |
| MMP9 | 0.00 | 0.75 | GSE36446 | cortex | NOX2 |  |  |  |  |  |  |  |
| IGFBP4 | 0.01 | 0.90 | GSE36446 | medulla | NOX2 |  |  |  |  |  |  |  |

**Supplemental Table 17F. LIUS-downregulated genes in pre-steoblast cells are classified into four groups, reactive oxygen species (ROS)-dependent; ROS-suppressed; ROS-dependent/suppressed; and ROS-independent.** Seven out of total 17 (41.18%) downregulated genes in LIUS-treated pre-osteoblast are identified as the ROS-dependent downregulations, which were upregulated in Nrf2-knocked out dataset (GSE7810) or/and downregulated in NOX2-knocked out dataset (GSE35446). Meanwhile, 6 out of 17 (41.18%) genes are identified as the ROS-suppressed downregulations. Four genes 4 (23.53%) are shared.

| downregulated in Pre-osteoblast | | | | | | | | | | | | |
| --- | --- | --- | --- | --- | --- | --- | --- | --- | --- | --- | --- | --- |
| ROS promoter genes | p value | Fold Change | dataset | tissue | KO/Inhibit Target | ROS inhibitor genes | p value | Fold Change | dataset | tissue | KO/Inhibit Target | Dual |
| BLNK | 0.00 | 2.52 | GSE7810 | liver | Nrf2 | Tmeff2 | 0.02 | 0.68 | GSE7810 | liver | Nrf2 | HMMR |
| FABP4 | 0.00 | 2.44 | GSE7810 | liver | Nrf2 | EDN1 | 0.01 | 0.47 | GSE7810 | liver | Nrf2 | Pmaip1 |
| IL1RN | 0.00 | 1.96 | GSE7810 | liver | Nrf2 | CDH1 | 0.01 | 0.40 | GSE7810 | liver | Nrf2 | Tmeff2 |
| HMMR | 0.01 | 1.43 | GSE7810 | liver | Nrf2 | Pmaip1 | 0.00 | 0.23 | GSE7810 | liver | Nrf2 | CDH1 |
| Pmaip1 | 0.01 | 0.89 | GSE36446 | cortex | NOX2 | HOMER2 | 0.00 | 0.22 | GSE7810 | liver | Nrf2 |  |
| Tmeff2 | 0.04 | 0.86 | GSE36446 | cortex | NOX2 | KRT14 | 0.00 | 0.16 | GSE7810 | liver | Nrf2 |  |
| CDH1 | 0.00 | 0.61 | GSE36446 | medulla | NOX2 | KRT14 | 0.00 | 1.25 | GSE36446 | cortex | NOX2 |  |
|  |  |  |  |  |  | Tmeff2 | 0.03 | 1.33 | GSE36446 | medulla | NOX2 |  |
|  |  |  |  |  |  | HMMR | 0.00 | 1.28 | GSE36446 | medulla | NOX2 |  |

**Supplemental Figure 1. The Ingenuity Pathway Analysis (IPA) profiles general innatomic genes**, which can be categorized by diseases or functions via number of genes. The five groups with the smallest P value are highlighted in green boxes by IPA, which include: 1) quantity of leukocytes; 2) quantity of blood cells; 3) necrosis; 4) proliferation of blood cells; and 5) apoptosis.
